# Brain Volumes After Hypertensive Pregnancy and Postpartum Blood Pressure Management: A POP-HT Randomized Clinical Trial Imaging Substudy

**DOI:** 10.1001/jamaneurol.2025.5145

**Published:** 2026-01-05

**Authors:** Winok Lapidaire, Jamie Kitt, Samuel Krasner, Paul A. Bateman, Hannah R. Cutler, Logan Barr, Annabelle Frost, Katherine Tucker, Katie Suriano, Yvonne Kenworthy, George Milner, Miriam Lacharie, Rebecca Mills, Cristian Roman, Lucy Mackillop, Christina Aye, Alexandra Cairns, Basky Thilaganathan, Lucy C. Chappell, Adam J. Lewandowski, Richard J. McManus, Paul Leeson

**Affiliations:** 1 Division of Cardiovascular Medicine, Radcliffe Department of Medicine, University of Oxford, Oxford, United Kingdom; 2 NIHR Oxford Biomedical Research Centre, Oxford, United Kingdom; 3 Nuffield Department of Primary Care Health Sciences, University of Oxford, Oxford, United Kingdom; 4 Queen’s University School of Medicine, Kingston, Ontario, Canada; 5 Oxford Centre for Clinical Magnetic Resonance Research, University of Oxford, Oxford, United Kingdom; 6 Institute of Biomedical Engineering, Department of Engineering Science, University of Oxford, Oxford, United Kingdom; 7 Nuffield Department of Women’s and Reproductive Health, University of Oxford, Oxford, United Kingdom; 8 Fetal Medicine Unit, Oxford University Hospitals NHS Foundation Trust, Oxford, United Kingdom; 9 Fetal Medicine Unit, St George’s University Hospitals NHS Foundation Trust, United Kingdom and Molecular and Clinical Sciences Research Institute, St George’s University of London, United Kingdom; 10 King’s College London and Honorary Consultant Obstetrician at Guy’s and St Thomas’ NHS Foundation Trust, London, United Kingdom; 11 Nuffield Department of Population Health, University of Oxford, Oxford, United Kingdom; 12 Brighton and Sussex Medical School, University of Brighton and University of Sussex, Brighton, United Kingdom

## Abstract

**Importance:**

Hypertensive pregnancy increases risk of cognitive decline, stroke, and dementia, especially after preeclampsia. Women with prior hypertensive pregnancy show lower brain volumes, but it was unknown whether early postpartum blood pressure optimization could alter these outcomes.

**Objective:**

To evaluate whether an intervention designed to achieve better postpartum blood pressure control after a hypertensive pregnancy is associated with differences in brain volumes around 9 months post partum compared with usual care.

**Design, Setting, and Participants:**

This randomized clinical trial, the Physician Optimized Postpartum blood pressure self-management trial (POP-HT), was a prospective, open-label, blinded end-point study conducted at a single tertiary center in the UK. Participants were older than 18 years and had preeclampsia or gestational hypertension requiring antihypertensive treatment at hospital discharge. Enrollment began February 21, 2020; the last follow-up was on November 2, 2021; and the mean follow-up time was approximately 9 months. Secondary outcome analyses (primary results published 2022) were conducted May 2025.

**Interventions:**

Telemonitored self-management with research physician-guided titration or usual postnatal care.

**Main Outcomes and Measures:**

This substudy analyzed the secondary outcomes T1-weighted magnetic resonance imaging (MRI) brain volumes (gray matter, white matter, cerebrospinal fluid, subcortical structures) acquired approximately 9 months post partum. Analyses used linear regression models adjusted for total intracranial volume.

**Results:**

Of 252 eligible participants, 32 declined, and 220 were randomized (mean [SD] age, 33.4 [5.1] years). The T1 brain MRI of 157 participants was available for analysis; 96 participants (63%) had preeclampsia, and 56 (37%) had gestational hypertension. The intervention group (n = 81) had larger total white matter volumes (adjusted mean difference, 11.50 cm^3^; 95% CI, 2.04 to 20.96; *P* = .02) compared with the usual care group (n = 71). In usual care, participants with preeclampsia had smaller putamen (adjusted mean difference, −0.83 cm^3^; 95% CI, −1.20 to −0.46; *P* < .001), accumbens (adjusted mean difference, −0.15 cm^3^; 95% CI, −0.24 to −0.05; *P* = .003), and pallidum (adjusted mean difference, −0.13 cm^3^; 95% CI, −0.26 to −0.01; *P* = .04) volumes compared with those with gestational hypertension. These differences were not observed in the intervention group.

**Conclusions and Relevance:**

This study found that short-term postpartum optimization of blood pressure control after hypertensive pregnancy was associated with larger brain volumes during the first year post partum. Because brain volume is a surrogate of brain health linked to tissue preservation and cognitive outcomes, these findings suggest potential neurovascular benefits that were most pronounced among women with preeclampsia.

**Trial Registration:**

ClinicalTrials.gov Identifier: NCT04273854

## Key Points

**Question:**

Can physician-optimized self-management of blood pressure during the early postpartum period after hypertensive pregnancy alter brain volumes?

**Findings:**

This randomized clinical trial found that physician-optimized blood pressure self-management was associated with higher white matter volumes compared with usual care. For participants receiving usual care, preeclampsia was associated with lower putamen, accumbens, and pallidum volumes compared with gestational hypertension, which was ameliorated by physician-optimized blood pressure self-management.

**Meaning:**

The early postpartum period may present an opportunity to improve long-term cognitive outcomes in women who have had a hypertensive pregnancy, and the negative impact of preeclampsia on the brain may be mitigated by improved postpartum blood pressure control.

## Teaser

This randomized clinical trial examines brain volumes in a secondary analysis of data for participants who had a hypertensive pregnancy to evaluate an intervention designed to achieve better postpartum blood pressure control.

## Introduction

Hypertensive disorders of pregnancy (HDPs) complicate up to 15% of pregnancies.^1^ Severity ranges from temporary mild hypertension to multiorgan systemic organ involvement, occasionally with cerebral edema and seizures. HDPs are also a key female-specific risk factor for later neurological conditions: cognitive problems,^2,3,4^ a 70% increased risk of stroke,^5^ cerebral small vessel disease,^6^ and a 26% increased risk of dementia,^7,8^ Furthermore, the risk of vascular dementia^9,10^ and stroke^5^ is at least twice as high after preeclampsia with a normotensive pregnancy.^5^

Lower brain volumes have also been seen in women with preeclampsia, during and shortly after pregnancy,^11^ in midlife,^12^ and in later life.^13^ Such reductions can be an early feature of developing neurological conditions.^14^ Some changes may reflect hypertensive exposure: acute hypertension at any point in life is linked to lower volumes in subcortical structures (caudate, putamen, nucleus accumbens, pallidum and thalamus,^15^ and hippocampus).^16,17^ Subcortical and periventricular brain injury also occurs with systemic microvascular dysfunction, seen in HDPs.^18^ Brain volume typically decreases during pregnancy and partially recovers post partum,^19^ but recovery may be limited by suboptimal vascular and cardiac health after HDPs. Together, these observations suggest that enhancing postpartum recovery could help preserve long-term brain health, yet whether postpartum interventions can modify brain volumes remains unknown.

We previously showed in a randomized trial that optimizing antihypertensive medication in the weeks after delivery improved blood pressure control^20^ and reversed adverse cardiovascular remodeling^20,21^ by 9 months post partum. Such vascular and cardiac improvements may directly benefit, or mirror, cerebral microvascular health. Recovery of brain volume may be particularly important in women after HDPs, given their potentially adversely affected brain structure. In this study, we therefore examined the intervention’s association with brain volumes. Because some evidence suggests that pathophysiological changes to the blood-brain barrier and cerebral vasculature may be unique to preeclampsia and are not seen in gestational hypertension,^7^ we tested whether effects differed between these groups.

## Methods

### Study Design and Participants

The Physician Optimized Postpartum blood pressure self-management trial (POP-HT) was a single-center, 2-group parallel, prospectively randomized, open, blinded end-point study (see trial protocol in Supplement 1). The full methods are reported in the protocol article.^22^ Briefly, eligible participants were women 18 years or older with a clinician-confirmed diagnosis of gestational hypertension or preeclampsia during their pregnancy who required antihypertensive medication at the time of hospital discharge from the Women’s Centre of Oxford University Hospitals NHS Foundation Trust in the UK. Exclusion criteria were chronic hypertension before pregnancy, antihypertensive use before pregnancy, hypertension at the routine antenatal 12-week check, conditions making self-monitoring impractical or unsafe, inability to use the English app, and inability to provide written consent. Race and ethnicity were self-reported by patients using National Institute for Health Research categories.

The trial was registered (ClinicalTrials.gov NCT04273854), approved by the London-Surrey Research Ethics Committee (reference 19/L0/1901), and overseen by a trial steering and data and safety monitoring committee. All participants provided written informed consent.

### Intervention and Randomization

Participants in the intervention arm uploaded daily home blood pressure measurements using a Bluetooth-enabled blood pressure monitor (EVOLV; Omron) via a bespoke trial app. Monitoring increased to twice daily when readings were outside predefined targets.^22^ As detailed in the protocol article,^22^ these readings were used to guide remote antihypertensive medication dose titration, in line with the 2019 NICE guideline.^23^ Initial discharge medications were prescribed by the NHS clinical team, and subsequent titration changes were initiated by study physicians.

Usual care followed NICE guidance,^23^ typically including community midwife blood pressure review at 3 to 5 days and 7 to 14 days post partum and a 6- to 8-week review with a family physician or specialist. Randomization was 1:1 via secure web-based software (Castor EDC) with minimization by gestational age at delivery, hypertensive pregnancy subtype (preeclampsia or gestational hypertension), and angiotensin converting enzyme inhibitor prescription at randomization. The trial was open label, but brain magnetic resonance imaging (MRI) processing was performed masked to group allocation.

### Procedures

The full study protocol comprised 4 study visits, but only data from the baseline study visit at 1 to 6 days post partum and the final visit at approximately 9 months post partum were used for analyses presented here. At the baseline visit, medical history, demographic information, and blood pressure measurements were collected. At the final visit, brain MRI data were acquired using a 3-T scanner (PRISMA; Siemens Healthineers). A T1-weighted sequence (TR/TE = 2040/4.7 milliseconds, flip angle 8°, FOV 200 mm, voxel size 1.0 mm isotropic) was acquired for structural volumetric measurements. MRI measurements were added as a protocol amendment to assess brain structure.

### MRI Processing

T1-weighted images were processed using the FSLanat pipeline^24^ to create gray matter segmented images and a T1 to the MNI registration matrix. This pipeline uses BET^25^ to extract the brain from the skull and surrounding area; FSL FAST^26^ to segment the brain into gray matter, white matter, and cerebrospinal fluid; and FSL FIRST^27^ to segment subcortical structures. Volumes were calculated by the number of voxels within the segmentation masks. All images were checked for quality by a single investigator (W.L.).

### Outcomes

The main brain outcomes were differences in total gray matter, white matter, and cerebrospinal fluid volumes. Volumes of subcortical structures associated with hypertension were also selected as outcome measures for additional analyses, including the caudate, putamen, nucleus accumbens, pallidum, thalamus,^15^ and hippocampus.^16,17^ These volumetric brain outcomes were not prespecified. Brain volumes are reported in cubic centimeters (cm^3^; equivalent to milliliters).

### Statistical Methods

Because preintervention brain MRI data were not collected, change could not be measured directly, but randomization supports comparable baseline brain volumes between groups. Primary analyses used modified intention to treat, preserving randomization irrespective of adherence. Linear regressions adjusted for total intracranial volume compared brain volume by trial arm and pregnancy history (preeclampsia vs gestational hypertension). To test whether the intervention effect differed by pregnancy history, a full cohort regression model was used, including trial arm, pregnancy history, and their interaction. We report adjusted mean differences with 95% CIs and 2-sided *P* values (α = .05). Given the exploratory nature, unadjusted *P* values are presented. As a secondary analysis, we applied the Benjamini-Hochberg false discovery rate procedure to correct for multiple comparisons across gray and white matter and across subcortical volumes. Sensitivity analyses were performed adjusting for baseline mean diastolic blood pressure and number of previous pregnancies.

We first (1) estimated intervention effects in all randomized participants with brain MRI data, and then (2) compared volumes by pregnancy history in the usual care arm, and finally, (3) for volumes differing by history, we assessed intervention associations. Analyses were performed using R version 4.3.1 (R Foundation).

## Results

### Study Population

**Figure 1.**
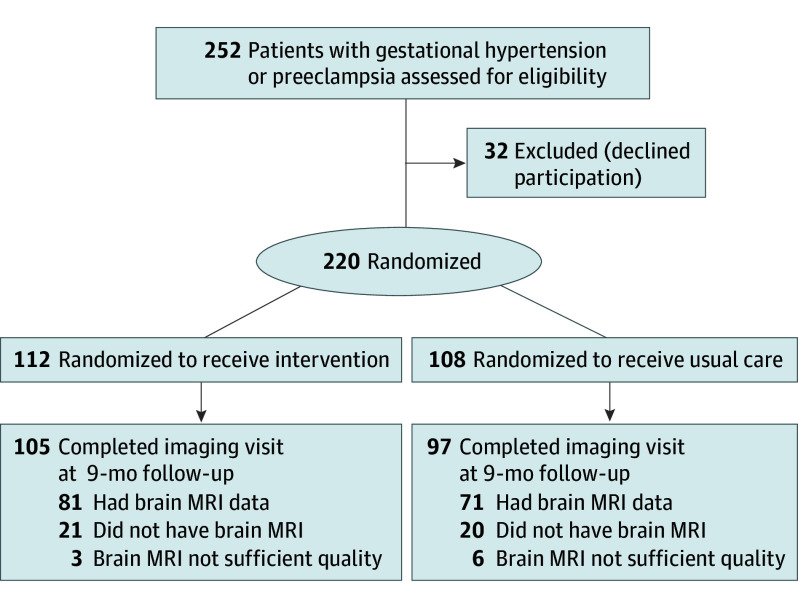
Flowchart for the POP-HT Trial Brain Magnetic Resonance Imaging (MRI) Substudy

Of the 220 participants enrolled in the POP-HT trial between February 2020 and November 2021, 112 were assigned to the intervention arm and 108 to the usual care arm. Of these, 202 (92%) completed the final study visit and 157 (71%) underwent brain MRI. The T1 brain scans of 152 (69%) participants (81 assigned to the intervention arm and 71 to the usual care arm) were of sufficient quality for analysis (Figure 1).

The proportions of preeclampsia (96 participants, 63%) and gestational hypertension (56 participants, 37%) were similar in both intervention and usual care arms, allowing for a prespecified subgroup analysis by type of pregnancy hypertension. The participant characteristics organized by pregnancy history are described in the Table. Demographic data for all randomized participants and those who had brain MR were similar (eTable 1 in Supplement 2). Participants in the intervention groups had a higher proportion with a prior hypertensive pregnancy than in the usual care group, and participants with preeclampsia were more likely to have a history of a previous hypertensive pregnancy compared with the gestational hypertension group. All participants in the intervention group who underwent MRI scans had received the intervention.

**Table.** Participant Characteristics by Diagnosis of Gestational Hypertension or Preeclampsia and Intervention Allocation

Characteristic	Intervention		Usual care	
	Preeclampsia (n = 51)	Gestational hypertension (n = 30)	Preeclampsia (n = 45)	Gestational hypertension (n = 26)
**Patient characteristics**				
Age, mean (SD), y	34.3 (5.3)	33.4 (5.3)	33.1 (5.2)	32.5 (4.6)
Booking BMI, mean (SD)^a^	28.0 (5.1)	29.4 (6.2)	30.2 (8.9)	27.5 (6.7)
Baseline BP, mean (SD), mm Hg	129.8 (12.5)/84.3 (10.4)	124.9 (13.1)/79.2 (10.7)	126.8 (15.6)/81.1 (12.1)	125.7 (11.2)/79.1 (10.2)
Imaging visit BP, mean (SD)	125.7 (11.9)/81.4 (7.7)	128.1 (8.4)/83.9 (8.9)	128.4 (12.3)/84.7 (9.1)	130.9 (11.2)/85.7 (8.1)
Race and ethnicity, No. (%)^b^				
Asian	6 (12)	1 (3)	2 (4)	3 (12)
Black	4 (8)	2 (6)	1 (2)	0
Hispanic	1 (2)	1 (3)	2 (4)	0
White	40 (78)	26 (87)	40 (89)	23 (88)
**Pregnancy characteristics**				
Gestation at delivery, median (IQR), wk	38 (36.6-39.4)	40 (39.3-40.6)	37.4 (35.4-39.6)	39.7 (39.1-40.7)
Previous hypertensive pregnancy, No. (%)	13 (25)	11 (37)	6 (13)	2 (8)
Birth weight, mean (SD), kg	2.9 (0.9)	3.3 (0.6)	2.9 (1.0)	3.3 (0.5)
Neonatal unit admissions, No. (%)	15 (29)	4 (13)	20 (44)	4 (15)

Abbreviations: BMI, body mass index; BP, blood pressure.

^a^ Calculated as weight in kilograms divided by height in meters squared.

^b^ Race and ethnicity were self-reported by patients using National Institute for Health Research categories.

### Intervention Effect in the Full Cohort

**Figure 2.**
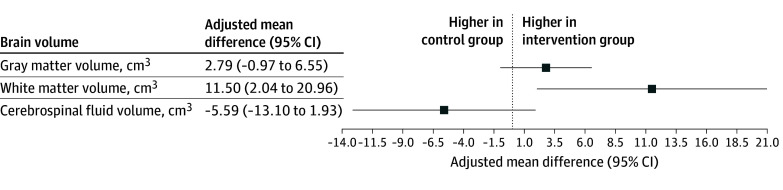
Forest Plot of Brain Volumes in the Intervention Arm Relative to the Usual Care Arm Brain volumes at 6 to 12 months post partum adjusted for total intracranial volume using linear regression models.

The intervention was associated with significantly larger white matter volumes (adjusted mean difference, 11.50 cm^3^; 95% CI, 2.04 to 20.96; *P* = .02). There were no significant intervention effects on the gray matter, cerebrospinal fluid, and individual subcortical volumes (Figure 2 and eTable 2 in Supplement 2). The observed intervention effect on white matter volume remained statistically significant after correcting for multiple comparisons. Findings were materially unchanged after additional adjustment for baseline mean diastolic blood pressure (gray matter adjusted mean difference, 3.00 cm^3^; 95% CI, −1.00 to 6.57; white matter adjusted mean difference, 11.00 cm^3^; 95% CI, 1.16 to 19.88; cerebrospinal fluid adjusted mean difference, −6.00 cm^3^; 95% CI, −13.14 to 2.01). When adjusting for the number of previous pregnancies, both the total white matter volume (adjusted mean difference, 11.31 cm^3^; 95% CI, 1.71 to 20.92) and putamen volume (adjusted mean difference, 0.28 cm^3^; 95% CI, 0.02 to 0.53) were significantly higher in the intervention group.

### Brain Volume Differences Between Preeclampsia vs Gestational Hypertension in the Usual Care Arm

In the usual care arm, participants with preeclampsia showed significantly lower putamen (adjusted mean difference, −0.83 cm^3^; 95% CI, −1.20 to −0.46; *P* < .001), accumbens (adjusted mean difference, −0.15 cm^3^; 95% CI, −0.24 to −0.05; *P* = .003), and pallidum (adjusted mean difference, −0.13 cm^3^; 95% CI, −0.26 to −0.01; *P* = .04) volumes, but no significant differences in gray matter and cerebrospinal volume or other subcortical volumes (eTable 3 in Supplement 2).

After correction for multiple comparisons, the associations for putamen and accumbens volumes remained statistically significant, whereas the association for pallidum volume did not. When adjusting for baseline mean diastolic blood pressure, putamen (adjusted mean difference, −0.81 cm^3^, 95% CI, −1.19 to −0.44), accumbens (adjusted mean difference, −0.15 cm^3^, 95% CI, −0.25 to −0.05), and pallidum (adjusted mean difference, −0.14 cm^3^, 95% CI, −0.26 to −0.01) volumes remained smaller in the preeclampsia group compared with the gestational hypertension group. Additionally, the white matter volume was significantly lower in the preeclampsia group (adjusted mean difference, −14.76 cm^3^; 95% CI, −29.30 to −0.23). When adjusting for the number of previous pregnancies, the putamen (adjusted mean difference, −0.84 cm^3^; 95% CI, −1.21 to −0.46), accumbens (adjusted mean difference, −0.15 cm^3^; 95% CI, −0.24 to −0.05), and pallidum (adjusted mean difference, −0.13 cm^3^; 95% CI, −0.26 to −0.01) volumes also remained smaller in the preeclampsia group compared with the gestational hypertension group.

### Intervention Effect in the Preeclampsia Group and Gestational Hypertension Group Full Cohort

**Figure 3.**
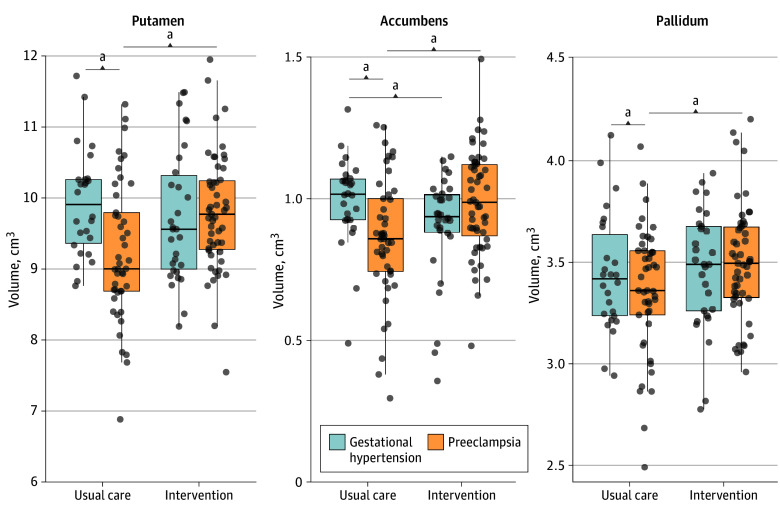
Subcortical Volumes for the Gestational Hypertension and Preeclampsia Groups in the Intervention and Usual Care Arms ^a^*P* < .05.

**Figure 4.**
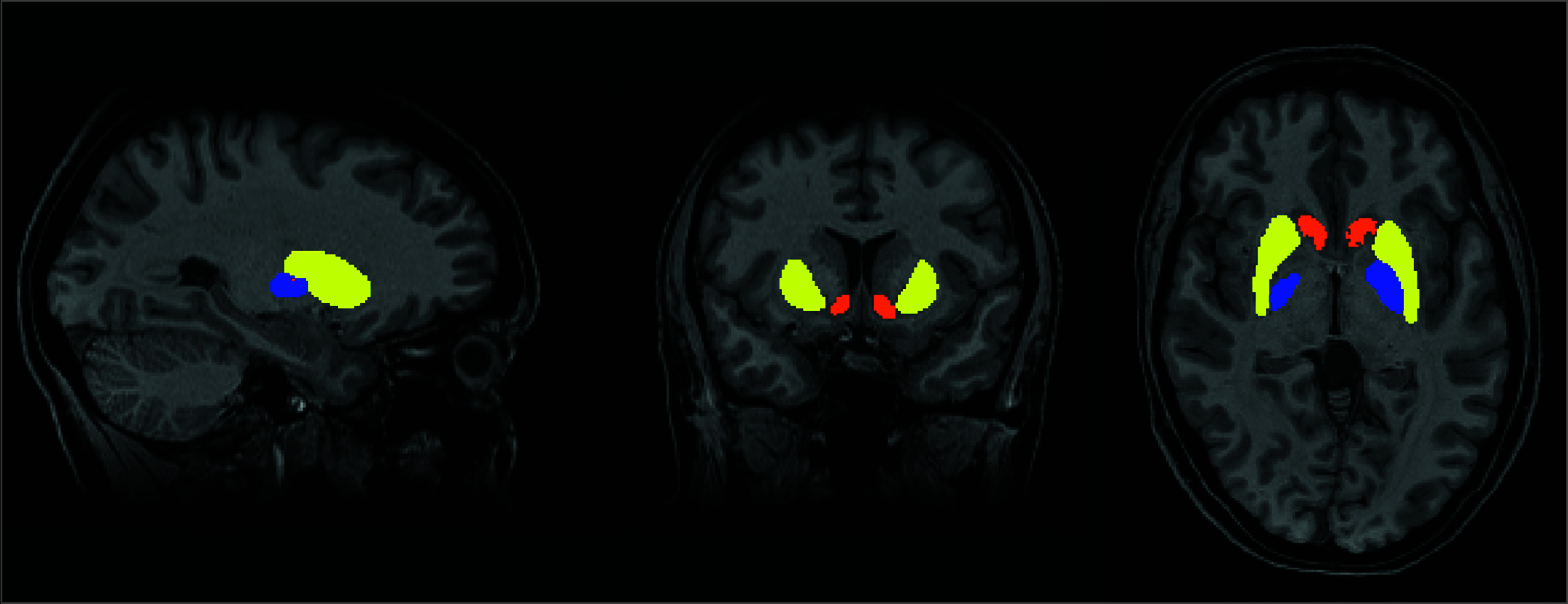
Magnetic Resonance Images of the Segmented Putamen, Accumbens, and Pallidum in a Trial Participant Yellow indicates the putamen; orange, accumbens; and blue, pallidum.

In the subcortical structures that were significantly different between preeclampsia and gestational hypertension groups in the usual care arm, when stratified by pregnancy history, the intervention was associated with significantly larger volumes in the preeclampsia group (putamen adjusted mean difference, 0.59 cm^3^; 95% CI, 0.29 to 0.89; *P* < .001; accumbens adjusted mean difference, 0.13 cm^3^; 95% CI, 0.06 to 0.20; *P* < .001; pallidum adjusted mean difference, 0.14 cm^3^; 95% CI, 0.04 to 0.25; *P* = .001), and these effects remained statistically significant after correction for multiple comparisons. In contrast, no significant intervention effects were seen in the gestational hypertension group for the putamen or pallidum (putamen adjusted mean difference, −0.37 cm^3^; 95% CI, −0.77 to 0.03; *P* = .07; pallidum adjusted mean difference, −0.03 cm^3^; 95% CI, −0.17 to 0.10; *P* = .66). A modest reduction in accumbens volume (accumbens adjusted mean difference, −0.11 cm^3^; 95% CI, −0.21 to −0.01; *P* = .03) was robust after correction for multiple comparisons (Figure 3). The formal test of interaction confirmed that the intervention effects differed significantly between pregnancy history groups (interaction *P* < .001 for putamen and accumbens, *P* = .04 for pallidum). Differences in these subcortical volumes between participants with preeclampsia and gestational hypertension in the intervention group are shown in eTable 4 in Supplement 2 and Figure 3, and images of a segmented putamen, accumbens, and pallidum in a trial participant are shown in Figure 4.

Adjusting for baseline diastolic blood pressure did not materially change the results in the preeclampsia group (putamen adjusted mean difference, 0.60 cm^3^; 95% CI, 0.29 to 0.90; accumbens adjusted mean difference, 0.12 cm^3^; 95% CI, 0.05 to 0.20; pallidum adjusted mean difference, 0.14 cm^3^; 95% CI, 0.04 to 0.24) or in the gestational hypertension group (putamen adjusted mean difference, −0.37 cm^3^; 95% CI, −0.77 to 0.04; pallidum adjusted mean difference, −0.03 cm^3^; 95% CI, −0.17 to 0.10; accumbens adjusted mean difference, −0.11 cm^3^; 95% CI, −0.21 to −0.01).

Adjusting for number of previous pregnancies did also not materially change the results in the preeclampsia group (putamen adjusted mean difference, 0.62 cm^3^; 95% CI, 0.32 to 0.92; accumbens adjusted mean difference, 0.14 cm^3^; 95% CI, 0.06 to 0.21; pallidum adjusted mean difference, 0.014 cm^3^; 95% CI, 0.04 to 0.24) or in the gestational hypertension group (putamen adjusted mean difference, −0.32 cm^3^; 95% CI, −0.72 to 0.08; pallidum adjusted mean difference, −0.04 cm^3^; 95% CI, −0.18 to 0.09; accumbens adjusted mean difference, −0.10 cm^3^; 95% CI, −00.20 to 0.00).

## Discussion

This randomized clinical trial indicates that a postpartum blood pressure management intervention after hypertensive disorders of pregnancy may be associated with favorable brain structure during the first year post partum. The intervention was linked to larger white matter volumes across women with hypertensive pregnancy (gestational hypertension and preeclampsia). In addition, women with a history of preeclampsia in the usual care arm showed smaller subcortical brain volumes at 6 to 9 months post partum than those with gestational hypertension; these differences were not evident among women in the intervention arm.

Both women with preeclampsia and gestational hypertension experience high blood pressure during pregnancy that frequently persists post partum.^28^ Lower white matter integrity has been reported from the peripartum period into later life.^3,12,29^ Hypertension-related white matter injury^30,31^ is associated with slower processing speed, executive dysfunction, and memory impairment.^31^ Although cognitive impact may not be obvious in the early postpartum period, white matter changes predict later cognitive decline and dementia,^32^ and converging longitudinal evidence suggests that reductions in white matter volume and integrity track cognitive decline, supporting the interpretation that better-preserved white matter is beneficial.^33^

Whether postpartum white matter changes are preventable or reversible had not been investigated. In this randomized clinical trial, a short-term blood pressure control intervention was associated with larger brain volumes several months later, when most participants were no longer taking antihypertensive medication. This is consistent with the postpartum period as a critical window for pregnancy-associated brain volume and blood pressure changes. Because baseline brain MRIs were not acquired, we cannot distinguish recovery of pregnancy-related changes from a slower postpregnancy decline relative to usual care.

We observed that women who had preeclampsia had smaller putamen, pallidum, and accumbens subcortical structures compared with those who had gestational hypertension in the usual care arm, and the intervention was associated with increases in these subcortical structures. The pathophysiology of preeclampsia, placental ischemia,^34^ and systemic endothelial dysfunction can disrupt the blood-brain barrier^35,36^ and cerebral blood circulation,^36,37,38^ potentially placing subcortical nuclei at particular risk because they depend on terminal arteriolar supply.^39^ It is also possible that the intervention’s effect appeared stronger in the preeclampsia group because they were more often taking multiple antihypertensive agents.

We observed no intervention effect on total gray matter or cerebrospinal fluid volume. Prior studies comparing women who previously had preeclampsia with those after normotensive pregnancies report inconsistent cortical gray matter differences.^12,40^ Cortical gray matter is more sensitive to chronic hypertension and cardiovascular health than acute hypertensive episodes,^41^ suggesting that cortical gray matter benefits of the postpartum blood pressure intervention may emerge over longer time periods as lower blood pressure persists. Continued follow-up is warranted.

Although human data on subcortical plasticity are limited, some recovery after injury is possible.^42^ Furthermore, exercise interventions can increase brain vessel lumen size and blood flow in young adults with elevated blood pressure,^43^ and intensive blood-pressure lowering slows progression of MRI white matter hyperintensities,^44^ though effects on subcortical gray matter volumes have been little studied in randomized clinical trials. Improved postpartum blood pressure control could permit microvascular recovery, improving perfusion of subcortical gray matter, aiding recovery or preventing ongoing injury in the months after delivery.^34^ Because the accumbens, putamen, and pallidum are part of the basal ganglia, involved in motor skills, habit formation, mood regulation, and cognition,^40^ structural preservation could have functional relevance, but we did not assess cognition in this study. In the absence of longer-term follow-up, the relationship between these volumetric findings and cognitive outcomes remains uncertain. Future work should test whether postpartum blood pressure optimization confers durable neurocognitive benefits, particularly in women with preeclampsia.

### Limitations

This study has limitations. First, the intervention effect may have been diluted because some participants in the usual care arm self-monitored during the COVID-19 pandemic.^20^ By chance, more women with a prior hypertensive pregnancy were randomized to the intervention arm, which could attenuate detectable differences if earlier hypertensive exposure had already influenced brain volumes. Third, the intervention was unblinded, but image processing and outcome analysis were conducted by staff blinded to allocation. Fourth, the brain imaging outcomes were secondary and analyses exploratory; results should be viewed as hypothesis generating and require replication. Because there was no normotensive pregnancy control group, we cannot determine how close the intervention group’s subcortical volumes came to a normotensive reference. Follow-up beyond 9 months is needed to establish durability. The overall sample size, and particularly the preeclampsia and gestational hypertension subgroups, was modest, limiting precision. Finally, this was a single-center study in a relatively homogeneous population managed by a small team of secondary care research clinicians; referral patterns and local practice may limit generalizability.

Future studies should investigate whether the intervention reduces the risk of developing chronic hypertension and thereby indirectly also protects the brain against hypertension-induced damage in the long term. Optimal implementation routes in routine care remain to be defined. A larger, multicenter trial with a normotensive control group, longer follow-up, and cognitive and clinical end points will be important to confirm these associations, clarify mechanisms, and test whether early postpartum blood-pressure optimization yields sustained neurological benefit, especially for women with preeclampsia.

## Conclusions

This study adds to the evidence that tighter blood pressure control in the immediate postpartum period benefits multiple organs. A postpartum intervention in the puerperium may benefit brain remodeling during a critical phase in women who have had hypertensive disorders of pregnancy.

## Notes

Supplement 1. Trial protocol

Supplement 2. **eTable 1.** Characteristics of all 220 participants enrolled in POP-HT and participants in the brain MRI sub-study **eTable 2.** Brain volumes in the intervention and usual care arm **eTable 3.** Brain volumes of the pre-eclampsia and gestational hypertension groups in the usual care arm **eTable 4.** Putamen, accumbens, and pallidum volumes of the pre-eclampsia and gestational hypertension groups in the intervention arm

Supplement 3. Data sharing statement
