# Supplementary material for: Brain Volumes After Hypertensive Pregnancy and Postpartum Blood Pressure Management: A POP-HT Randomized Clinical Trial Imaging Substudy
Source: JAMA Neurol. 2026 Jan 5;83(2):137–44. doi: 10.1001/jamaneurol.2025.5145 (PMC12771390; doi:10.1001/jamaneurol.2025.5145)
Supplement: Supplement 1. — Trial protocol [file jamaneurol-e255145-s001.pdf]

Protocol V6.0 23/03/2021 p.2

1. Key trial contacts
2. Lay summary
3. Synopsis
4. Abbreviations
5. Background and rationale
6. Objectives and outcome measures
7. Study design
8. Participant identification
9. Protocol procedures
10. Study intervention
11. Safety reporting
12. Statistics
13. Data management
14. Quality assurance projects
15. Protocol deviations
16. Serious breaches
17. Ethical and regulatory considerations
18. Finance and insurance
19. Publication policy
20. Development of a new product/process or the generation of intellectual property
21. Archiving
22. Appendices
23. References

Statistical analysis plan p.48

1. Abbreviations
2. Introduction
3. Trial design
4. Definition and derivation of outcome measures
5. Analysis: general considerations
6. Primary analysis
7. Secondary analysis
8. Sensitivity analysis
9. Additional exploratory analysis
10. Subgroup analysis and safety analysis
11. Validation
12. Changes previous versions of SAP
13. References

Protocol: Physician Optimised Post-partum Hypertension Treatment (POP-HT) trial

Date and Version No: V6.0 23rd March 2021

**Chief Investigator:**	Professor Paul Leeson, Director of Cardiovascular Clinical Research Facility (CCRF), Division of Cardiovascular Medicine, University of Oxford
**Investigators:**	Dr Jamie Kitt (DPhil student, Cardiovascular Clinical Research Facility (CCRF), Division of Cardiovascular Medicine, University of Oxford<br>Professor Richard McManus (Nuffield Department of Primary Care Health Sciences, University of Oxford)<br>Dr Lucy Mackillop (Consultant Obstetric Physician, Oxford University Hospitals NHS Trust, John Radcliffe Hospital)<br>Dr Adam Lewandowski (University Research Lecturer and British Heart Foundation Intermediate Research Fellow, Division of Cardiovascular Medicine, Radcliffe Department of Medicine, University of Oxford)<br>Professor Basky Thilaganathan, Director, Maternal-fetal Medicine Unit, St George's University Hospitals NHS Foundation Trust<br>Professor Lucy Chappell, NIHR Research Professor in Obstetrics<br>Dr Christina Aye, NIHR Academic Clinical Lecturer and Specialty trainee in Maternal Fetal Medicine<br>Dr Annabelle Frost, Obstetric Clinical Research Fellow, Cardiovascular Clinical Research<br>Facility (CCRF), Division of Cardiovascular Medicine, University of Oxford<br>Dr Katie Suriano, Research assistant, Cardiovascular Clinical Research Facility (CCRF), Division of Cardiovascular Medicine, University of Oxford<br>Mrs Yvonne Kenwothy , Senior research mid-wife Cardiovascular Clinical Research Facility (CCRF), Division of Cardiovascular Medicine, University of Oxford<br>Mrs Annabelle McCourt, Research assistant, Cardiovascular Clinical Research Facility (CCRF), Division of Cardiovascular Medicine, University of Oxford<br>Mr Logan Barr, B.Sc.(Honours), Queen's University, Canada
**Sponsor:**	University of Oxford Clinical Trials and Research Governance, Joint Research Office, 1st floor, Boundary Brook House, Churchill Drive, Headington. Oxford OX3 7GB Tel: 01865 61648<br>E-mail : ctrg@admin.ox.ac.uk
**Funder:**	The research is being financed by a British Heart Foundation Clinical Research Training Fellowship (BHF Grant number FS/19/7/34148) and National Institute for Health Research NIHR Collaboration for Leadership in Applied Health Research Oxford
**Chief Investigator Signature:**<br><br>**Statistician Signature:**	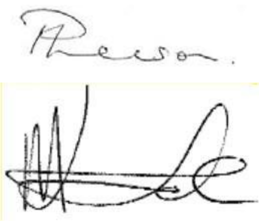 Date: 23/03/2021

1. Key trial contacts

**Chief Investigator**	Professor Paul Leeson, PhD, FRCP<br>Professor of Cardiovascular Medicine and Consultant Cardiologist<br>Oxford Cardiovascular Clinical Research Facility<br>Level 1, John Radcliffe Hospital<br>Oxford, England. OX3 9DU<br>Email : paul.leeson@cardiov.ox.ac.uk<br>Tel : 01865572846 Fax : 01865572840
**Sponsor**	University of Oxford<br>Clinical Trials and Research Governance<br>Joint Research Office<br>1st Floor, Boundary Brook House,<br>Churchill Drive<br>Headington, Oxford OX3 7GB<br>Email: ctrg@admin.ox.ac.uk
**Funder(s)**	British Heart Foundation Clinical Research Training Fellowship (BHF Grant number FS/19/7/34148)
**Statistician**	Name: Dr Jill Mollison<br><br>Organisation: Primary Health Care Sciences Clinical Trials Support Unit
**Committees**	**Joint Trial Steering Committee (TSC) and Data and Safety Monitoring Committee (DSMC)**<br>Internal members (1 vote each)<br>Professor Paul Leeson, Director of Cardiovascular Clinical Research Facility (CCRF), Division of Cardiovascular Medicine, University of Oxford<br>Dr Jill Mollison, Internal statistician for trial. Senior Trial Statistician, PHC CTU<br>Dr Jamie Kitt, BHF Research Fellow for the project<br><br>Four external members (1 vote each with net result that external members have the majority):<br>Dr Kate Bramham: Chief Investigator and Obstetric nephrologist, King's College London<br>Professor Christian Delles, Professor of Cardiovascular Prevention and Deputy Director of the BHF Centre of Excellence.<br>External Statistician: Rafael Perera-Salazar, PHC Statistics department<br>Lay representative/PPI representative: Emily Goodwin<br><br>**Trial Management Group**<br>Dr Jamie Kitt, BHF CRT-F and Honorary Cardiology Specialty trainee, Division Cardiovascular Medicine, University of Oxford<br>Mrs Yvonne Kenworthy, Division of Cardiovascular Medicine, University of Oxford<br>Dr Adam Lewandowski, BHF intermediate fellow and University lecturer, University of Oxford<br>Dr Katherine Tucker, Post-Doctoral Research Fellow, PHC<br>Dr Christina Aye, NIHR Academic Clinical Lecturer and Specialty trainee in Maternal Fetal Medicine<br>Dr Elena Benedetto, CCRF trial manager<br>Prof Richard McManus, Supervisor and Professor of Primary Care<br>Professor Paul Leeson, Director of Cardiovascular Clinical Research Facility (CCRF), Division of Cardiovascular Medicine, University of Oxford<br>Dr Jill Mollison, Internal statistician for trial. Senior Trial Statistician, PHC

	CTU<br>Dr Alexandra Cairns, Obstetric Specialty Registrar

2. Lay summary

We have shown in a pilot randomised controlled study [SNAP-HT [4]; REC 14/SC/1316] that blood pressure self-management during the post-partum period after hypertensive pregnancies, results in lower blood pressure after six months; even when medication has been stopped. We now want to assess whether this blood pressure reduction can be reproduced in a larger, randomised, single-blinded study and whether the blood pressure lowering has additional benefits in terms of other cardiovascular and cerebrovascular changes known to occur in women who have had a hypertensive pregnancy. We therefore plan to run a trial of self-management in the post-partum period, using updated Blue-tooth® enabled blood pressure monitoring coupled to physician-assisted dose titration to further advance the self-management aspect of the intervention. The physicians will be specialist clinicians who form part of the research team. We will measure additional structural and functional end organ differences, using magnetic resonance imaging of the brain and heart as well as echocardiography and retinal imaging. This will provide insight into the impact of post-partum blood pressure control on the maternal cardiovascular system and how this associates with blood pressure changes. Together, these studies will help refine future intervention strategies in this cohort of patients.

3. Synopsis

Trial Title	Physician Optimised Post-partum Hypertension Treatment trial
Internal ref. no. (or short title)	POP-HT
Trial registration	Registered on clinicaltrials.gov (NCT04273854)
Sponsor	University of Oxford<br>Clinical Trials and Research Governance<br>Clinical Trials and Research Governance<br>Joint Research Office<br>1st Floor, Boundary Brook House,<br>Churchill Drive<br>Headington, Oxford,<br>OX3 7GB Email: ctrg@admin.ox.ac.uk
Funder	British Heart Foundation Clinical Research Training Fellowship (BHF Grant number FS/19/7/34148)
Trial Design	Prospective Randomised Open Blinded End-point (PROBE)
Trial Participants	Women of child-bearing age 18years or over with a diagnosis of pre-eclampsia or gestational hypertension as defined by NICE guideline NG 133 (2019 update of CG107), and requiring anti-hypertensive medication at the point of hospital discharge
Sample Size	220 enrolled participants with 1:1 randomisation<br><br>Sub-study: 20 normotensive postnatal women
Planned Trial Period	31/12/19-01/12/30
Planned Recruitment period	31/12/19- 31/08/21

	Objectives	Outcome Measures	Timepoint(s)

Primary	To compare postpartum diastolic BP in the intervention arm to the control arm	24 hour average diastolic BP measured by assessed by SPACELAB 90217 24hr Ambulatory blood pressure monitoring (ABPM)	6-9 months postpartum
Secondary	To compare the effect of the intervention on cardiovascular, cerebrovascular and vascular phenotypes	BP based<br>a) 24 hr average systolic blood pressure assessed by SPACELAB 90217 24hr ABPM<br>b) Mean diurnal diastolic blood pressure assessed by SPACELAB 90217 ABPM<br>c) Mean diurnal systolic blood pressure assessed by SPACELAB 90217 ABPM<br>d) Mean nocturnal diastolic blood pressure assessed by SPACELAB 90217 24hr ABPM<br>e) Mean nocturnal systolic blood pressure assessed by SPACELAB 90217 24hr ABPM<br>f) Mean bedside diastolic blood pressure measured during study visit (mean of 2+3)<br>g) Mean bedside systolic blood pressure measured during study visit (mean of 2+3)<br>Cardiac MRI<br>h) Left ventricular (LV) mass indexed to end-diastolic volume and body surface area (BSA)<br>i) LV EDV indexed to BSA<br>j) LV wall thickness – septum, posterior and RWT<br>k) LA volume indexed to BSA<br>l) Right ventricular (RV) mass indexed to end-diastolic volume and body surface area (MRI)<br>m) RV EDV indexed to BSA<br>n) RA volume indexed to BSA<br>o) LV ejection fraction (EF) & RV EF<br>p) LV and RV stroke volumes indexed to BSA<br>q) Myocardial fibrosis<br>r) ECV (extra-cellular volume)<br>Echo<br>s) LV Diastolic function:<br>E/E' average, E/A ratio, E deceleration time<br>t) Global longitudinal strain (GLS)<br>u) LV systolic function (EF by Biplane Simpson's)<br>v) LA volume by Biplanar assessment<br>Vascular:<br>w) Pulse wave velocity<br>x) Augmentation index<br>y) Aortic BP<br>z) Aortic distensibility (MRI)	Week 6 and 6-9 months for the 24 hr ABPM<br><br>Baseline, week 1, week 6 and 6-9 months for the bedside blood pressures<br><br>For Cardiac MRI at 6-12 months post-partum<br>At baseline and at 6-12 months post-partum for Echo outcome measures<br><br>PWV, Aortic BP and AI at baseline and at 6-12 months<br>Aortic distensibility at 6-12 months

70

71

		Cerebrovascular<br>aa) Total white matter hyperintensity volume<br>bb) Cerebral blood flow<br>cc) Mean vessel thickness of the middle and posterior cerebral arteries and internal carotid artery<br><br>Retinal<br>dd) the corrected central retinal arteriolar equivalent<br>ee) the corrected central retinal venular equivalent<br>ff) corrected central retinal arteriolar equivalent/corrected central retinal venular equivalent ratio.	6 -12 months post-partum for all Brain MRI measures.<br><br>6-12 months postpartum for all retinal measures
Tertiary:	 <br>To explore in-vitro vascular function in a sub-study of 20 women<br><br>To explore presence/absence of kidney injury and fibro-inflammatory status<br><br>Quality of life assessment<br><br>Participant experience: assessment of individual experience following intervention	Exercise Echo<br>Exercise ejection fraction (echo) at 50% of peak workload during a bicycle cardio-pulmonary exercise test (CPET)<br>Exercise LA volume at 50% peak workload<br><br>CPET<br>VO2 at VT1<br><br>Assessment of endothelial cell function and circulating biomarker levels associated with vascular angiogenesis and inflammation in normotensive and hypertensive women to determine if BP improvement can affect vascular function<br><br>T1 mapping of the kidneys<br><br>EQ-5D-5L health questionnaire results<br><br>Qualitative semi-structured interviews in subset of individuals<br><br>Readmission number in each arm	6-12 months postpartum<br><br>6-12 months postpartum<br><br>From baseline to 6-12 months postpartum<br><br>6-12 months postpartum<br><br>Baseline, week 1, week 6 and 6-12 months postpartum<br><br>6-12 months postpartum<br><br>0-12 months postpartum

	Number of readmissions in intervention vs control arm	Number and frequency of side-effects reported (intervention via the app and control during follow up calls/SMS)	0-12 months postpartum
	Side-effect impact		
Intervention(s)	The intervention will consist of physician-optimised self-management of post-partum BP. Women will follow a ‘smartphone’ app-based algorithm for medication titration, which will provide individualised dose titration advice. This is overseen and any change is approved by physicians who review the uploaded readings and respond to tele-monitored abnormal readings in a timely fashion.		
Comparator	The control arm will be managed as per usual NHS-led care with assessment by their own health care professionals and adjustment of medications as required. The BP of this group will be monitored and recorded at the same time-points and in the same manner as the intervention arm as with all other secondary outcome measures.		

4. Abbreviations

AE	Adverse event
ABPM	Ambulatory Blood pressure monitoring
AHA	American Heart Association
AR	Adverse reaction
CI	Chief Investigator
CCRF	Cardiovascular Clinical Research Facility
CPET	Cardiopulmonary exercise test
CRF	Case Report Form
CT	Clinical Trials
CTRG	Clinical Trials and Research Governance
DMC/DMSC	Data Monitoring Committee / Data Monitoring and Safety Committee
ECV	Extra-cellular volume
ESC	European Society of Cardiology
GCP	Good Clinical Practice
GP	General Practitioner

HRA	Health Research Authority
ICF	Informed Consent Form
LV	Left ventricle
MHRA	Medicines and Healthcare products Regulatory Agency
NICE	National institute for clinical excellence
OCMR	Oxford Centre for Cardiovascular Magnetic Resonance Imaging
OIBME	Oxford Institute for Biomedical Engineering
NHS	National Health Service
NIHR	National Institute for Health Research
RES	Research Ethics Service
PI	Principal Investigator
PIS	Participant/Patient Information Sheet
PPE	Personal Protective Equipment
PW	Pulse Wave
R&D	NHS Trust R&D Department
REC	Research Ethics Committee
SAE	Serious Adverse Event
SOP	Standard Operating Procedure
TMF	Trial Master File

5. Background and rationale

Hypertensive disorders of pregnancy affect ~one in 10 pregnancies, which equates to >80,000 women per year in the UK [1]. Hypertension can persist post-partum but in many women has returned to the normal range within 6 months. As a result, the post-partum period has traditionally been considered a low risk period[2] but this time can be accompanied by rapid and often unpredictable changes in blood pressure.

One study showed that 50% of women with pre- eclampsia have persistent significant hypertension on day 5 following delivery [3], with blood pressure control remaining an issue for up to six weeks after childbirth, often requiring multiple medications and careful titration. At the same time, competing postnatal demands on mothers, not least from the new baby, can be associated with poor adherence and/or poor levels of clinical contact. Drug titration can therefore be sporadic exacerbating poorly controlled hypertension. Recently, a randomised controlled pilot study (SNAP-HT) run in Oxford [4] has suggested self-management of blood pressure in the post-partum period, through home blood pressure monitoring combined with a proprietary

smartphone app in which women entered the BP readings and receive tailored dose instructions, may offer a rational approach to improve blood pressure management in the post-partum period. This approach may be able to overcome some of the problems of poor adherence and drug titration.

High rates of compliance were achieved, and blood pressure control was better in the intervention group, with a mean systolic BP difference of 5.2mmHg and mean diastolic BP difference of 6mmHg, at 6 weeks. However, the most striking finding was that diastolic blood pressure remained a mean 4.5mmHg lower in the intervention group at six months' post-partum; even after the mother had ceased medication [see figure 1 below].

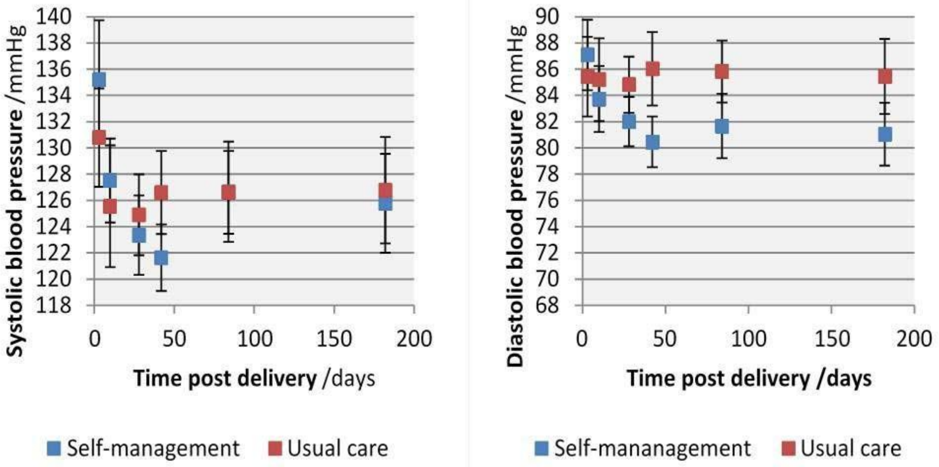

Figure 1: Comparison of systolic and diastolic BP readings at different time points post-delivery in the treatment vs usual care arms of SNAP-HT

These findings highlight the potential importance of post-partum blood pressure control in determining long-term cardiovascular risk for these women. Risk of higher blood pressure 5 to 10 years after a hypertensive pregnancy is related to blood pressures levels during the first few months post-partum [5] and a recent publication highlighted that a third of women after a hypertensive pregnancy present with chronic hypertension within the next 10 years [6]. Consistent with this, hypertensive disorders of pregnancy are associated with a two-fold increase in risk of subsequent cardiovascular disease [7, 8] and, recent ESC and AHA guidance have advised that such women should be informed of their increased risk [9, 10]. Moreover, levels of blood pressure in the general population are strongly related to long-term risk of cardiovascular disease and are the leading risk factor for loss of disability-adjusted life years (DALYs) in high and low-middle income countries. Every 10 systolic/5 diastolic mmHg of blood pressure reduction associates with a ~40% reduction in stroke risk and ~20% reduction in coronary heart disease lifetime risk [11]. Therefore, if the 4.5mmHg improvement in diastolic blood pressure seen in SNAP-HT were maintained long-term, it could correlate to a ~40% lifetime reduction in risk of stroke and ~20% lifetime reduction in risk of coronary heart disease risk.

If improving blood pressure control during the acute post-partum period, as achieved in the treatment arm of SNAP-HT, using a method that is both clinically and cost effective, results in a long term resetting of blood pressure, there is a compelling argument that post-partum blood pressure self-management should be a key part of clinical guidelines [12, 13]. However, several questions require answering to understand the potential clinical translational benefits of the findings in SNAP-HT.

Firstly, is the improvement in blood pressure, which was evident at 6 months' post- partum in SNAP-HT, still evident in the same cohort longer term or do other lifestyle factors known to be relevant to blood pressure potentially overwhelm or exaggerate differences in blood pressure in this cohort over the longer term? This was explored in the NIHR funded SNAP-HT Extension study.

Secondly, can similar blood pressure lowering to SNAP-HT be achieved making use of novel clinical tools? SNAP-HT used a proprietary self-monitoring smartphone app to guide dose titration, which required

significant patient involvement in blood pressure measurement, input and response to advised changes in medication. This technology proved promising but as well as testing the blood-pressure lowering reproducibility demonstrate in SNAP-HT through self-management [4], it is also important to start to gather pilot data on whether 'more-user-friendly' approaches can reproduce similar differences. For example, 'Blue-tooth enabled home blood pressure monitors will be used to automate the upload of blood pressure readings to a centralised server reducing the amount of 'time' and 'effort' women have to contributed themselves during this 'busy' time in life with a new-born baby/babies. Women will now have the option of adjusting their own medication based on the uploaded readings which will be reviewed and acted upon by specialist physicians who form part of the research team (medication changes will be based on the NICE NG133 2019 update [14]), Participants can still opt to see their own GP/midwife instead, or in addition, and all usual NHS care will be offered. This Blue-tooth method has been widely used in other studies and notably has been well validated by the OX-VASC group [15, 16].

Thirdly, does improved post-partum blood pressure control also result in reduced end organ damage in the cardiac, vascular and cerebrovascular systems? A significant finding in women who have had hypertensive pregnancies is early emergence of changes in cardiac, vascular and brain structure and function out of proportion to their cardiovascular risk profile [17-19]. This may explain why this group of women have a disproportionate risk of later cardio-vascular and cerebrovascular disease. It is possible these differences emerge during pregnancy and persist long term, independent of post-partum blood pressure variability but this is not really known and, although the significant cardiovascular adaptations that emerge during complicated pregnancies are known to reverse to some extent during the post-partum period [20-23], an alternative hypothesis it that the long term 'risk reflects a failure of the cardiac, vascular or cerebral systems to 'normalise' after pregnancy [24, 25]. Recent data from the C-MORE (Capturing MultiORgan Effects) of COVID-19 study, under peer review, has suggested inflammation in the kidneys plays a role in blood pressure control. Given that pre-eclampsia is known to cause cardio-renal dysfunction[26], exploration of the inflammatory state of the kidneys at 6-12 months post-partum has also been added as part of the study to explore this association.

If improved post-partum blood pressure control can influence cardio-vascular and vascular remodelling it may offer a new approach to modify these long-term end organ changes, in addition to any beneficial effects on blood pressure as demonstrated in SNAP-HT.

Description of the population to be studied

We aim to recruit 200 participants to the main study who have had a hypertensive pregnancy and who required anti-hypertensive medication at the time of discharge from hospital (as in the pilot study SNAPHT; REC 14/SC/1316) who have capacity to self-manage their blood pressure following discharge from hospital guided by physician-assistance remotely.

Safety and Benefits of the study

Data from large multi-centre randomised controlled trials have not suggested any significant risk associated with self-management of essential hypertension [27-29]. We aim to minimise any potential risks due to poor blood pressure control by ensuring women have clear advice regarding when to seek urgent medical help. This will be reinforced by messages from the tele-monitoring service when they submit a blood pressure reading, which is outside the target range. This will direct them where to seek medical attention depending on the severity of the reading. Participants will also be provided with a free telephone number for an advice line that will be answered by one of the specialist physicians who form part of the research team in normal working hours (9-5pm Monday-Friday). Outside of these hours, women will be asked to contact their usual NHS providers for any concerns/symptoms and to submit these via the app.   Women in the self-management group will also receive usual blood pressure monitoring and management by their community midwife and GP following their discharge from hospital. However, for safety it is important that participants in this group adhere to the home monitoring schedule. In the first 14 days after birth NICE recommends, that BP is monitored on at least alternate days. Therefore, in the first 2 weeks following discharge, if a participants fail to upload readings via the smart phone app after 24 hours they receive a notification to prompt and remind them. If at 36 hours a reading has still not been submitted the system will trigger the research team to contact participants to encourage them to take a reading, and to explore reasons why readings are not being done. Motivational SMS messages, developed with psychologist input and PPI feedback will also be used. From 14

days after delivery until treatment is discontinued, failure to report a blood pressure reading for 5 days will trigger the research team to contact a participant and if they refuse to engage, they will be withdrawn. If a participant withdraws / is withdrawn from the study, we will contact their GP/midwife to inform them, in order to ensure that any on-going care they require is reinstated.

In addition, it was also pleasing from the safety viewpoint that there were no SAEs within SNAP-HT [4] and the pending qualitative analysis was exceedingly positive in favour of empowering this group of women through self-management.

In terms of benefit, not only do we hope to demonstrate reproducibility of improved blood-pressure control in the intervention arm but furthermore, current national and international guidance for the post-partum care of pre-eclampsia and gestational hypertension lacks robust, high-quality evidence to guide practice and we hope to provide data that helps improve this aspect of care. Around 30% of eclamptic fits and over half of strokes associated with severe pre-eclampsia occur post-partum [3] due to poor home BP control and SNAP HT [4] demonstrated the feasibility of home blood pressure monitoring and drug titration up to 6 months post-partum. We also hope that the blood pressure improvement achieved will result in beneficial remodelling of the cardiovascular and cerebrovascular systems of women who had experienced a hypertensive pregnancy and mitigate their long-term cardiovascular risk, but even if this is not evident, the data will significantly develop our understanding of the implications of post-partum blood pressure through the use of sensitive MRI and vascular measures in the post-partum period.
The purpose of the sub study is to provide a reference population of women not affected by hypertensive disease. In the sub study, healthy postnatal women will undergo measurements of specific characteristics of blood cells and circulating factors involved in inflammation and endothelial dysfunction. This population will validate how blood cells and circulating factors vary naturally and may be affected by external factors such as mode of delivery.

6. Objectives and outcome measures

	Objectives	Outcome Measures	Timepoint(s)
Primary	To compare postpartum diastolic BP in the intervention arm to the control arm.	24 hour average diastolic BP measured by assessed by SPACELAB 90217 24hr Ambulatory blood pressure monitoring (ABPM)	6-9 months postpartum
Secondary	To compare the effect of the intervention on cardiovascular, cerebrovascular and vascular phenotypes	**BP based**<br>a) 24 hr average systolic blood pressure assessed by SPACELAB 90217 24hr ABPM<br>b) Mean diurnal diastolic blood pressure assessed by SPACELAB 90217 ABPM<br>c) Mean diurnal systolic blood pressure assessed by SPACELAB 90217 ABPM<br>d) Mean nocturnal diastolic blood pressure assessed by SPACELAB 90217 24hr ABPM<br>e) Mean nocturnal systolic blood pressure assessed by SPACELAB 90217 24hr ABPM<br>f) Mean bedside diastolic blood pressure measured during study visit (mean of 2+3)	Week 6 and 6-9 months for the 24 hr ABPM<br><br>Baseline, week 1, week 6 and 6-9 months for the bedside blood pressures

		g) Mean bedside systolic blood pressure measured during study visit (mean of 2+3)<br><br>**Cardiac MRI**<br>h) Left ventricular (LV) mass indexed to end-diastolic volume and body surface area (BSA)<br>i) LV EDV indexed to BSA<br>j) LV wall thickness – septum, posterior and RWT<br>k) LA volume indexed to BSA<br>Right ventricular (RV) mass indexed to end-diastolic volume and body surface area (MRI)<br>m) RV EDV indexed to BSA<br>n) RA volume indexed to BSA<br>o) LV ejection fraction (EF) & RV EF<br>p) LV and RV stroke volumes indexed to BSA<br>q) Myocardial fibrosis<br>r) ECV (extra-cellular volume)<br><br>**Echo**<br>s) LV Diastolic function: E/E' average, E/A ratio, E deceleration time<br>t) Global longitudinal strain (GLS)<br>u) LV systolic function (EF by Biplane Simpson's)<br>v) LA volume by Biplanar assessment<br><br>**Vascular**:<br>w) Pulse wave velocity<br>x) Augmentation index<br>y) Aortic BP<br>z) Aortic distensibility (MRI)<br><br>**Cerebrovascular**<br>aa) Total white matter hyperintensity volume<br>bb) Cerebral blood flow<br>cc) Mean vessel thickness of the middle and posterior cerebral arteries and internal carotid artery<br><br>**Retinal**<br>dd) the corrected central retinal arteriolar equivalent<br>ee) the corrected central retinal venular equivalent	For Cardiac MRI at 6-12 months post-partum<br><br>At baseline and at 6-12 months postpartum for Echo outcome measures<br><br>PWV, Aortic BP and AI at baseline and at 6-12 months Aortic distensibility at 612 months<br><br>6 -12 months post-partum for all Brain MRI measures.<br><br>6-12 months postpartum for all retinal measures

		corrected central retinal arteriolar equivalent/corrected central retinal venular equivalent ratio.	
Tertiary:		**Exercise Echo**<br>Exercise ejection fraction (echo) at 50% of peak workload during a bicycle cardio-pulmonary exercise test (CPET)<br>Exercise LA volume at 50% peak workload	6-12 months postpartum
		**CPET**<br>VO2 at VT1	6-12 months postpartum
	To explore in-vitro vascular function in a sub-study of 20 women	Assessment of endothelial cell function and circulating biomarker levels associated with vascular angiogenesis and inflammation in normotensive and hypertensive women to determine if BP improvement can affect vascular function	From baseline to 6-12 months postpartum
	To explore presence/absence of kidney injury and fibro-inflammatory status	T1 mapping of the kidneys	6-12 months postpartum
	Quality of life assessment	EQ-5D-5L health questionnaire results	Baseline, week 1, week 6 and 6-12 months postpartum
	Participant experience: assessment of individual experience following intervention	Qualitative semi-structured interviews in subset of individuals	6-12 months postpartum
	Number of readmissions in intervention vs control arm	Readmission number in each arm	0-12 months postpartum
	Side-effect impact	Number and frequency of side-effects reported (intervention via the app and control during follow up calls/SMS)	0-12 months postpartum

Intervention(s)	The intervention will consist of physician-optimised self-management of post-partum BP. Women will follow a ‘smartphone’ app-based algorithm for medication titration, which will provide individualised dose titration advice. This is overseen and any change is approved by physicians who review the uploaded readings and respond to tele-monitored abnormal readings in a timely fashion.
Comparator	The control arm will be managed as per usual NHS-led care with assessment by their own health care professionals and adjustment of medications as required. The BP of this group will be monitored and recorded at the same time-points and in the same manner as the intervention arm as with all other secondary outcome measures.

7. Study design

Design

The trial is a single centre, Prospective Randomised Open Blinded End-point (PROBE) study. Women who develop hypertensive disorders of pregnancy, which require on-going treatment for blood pressure after birth at the time of discharge, will be randomised to one of two treatment arms: usual care, or self-management of blood pressure. Women will be recruited from the John Radcliffe Hospital and this study will investigate the effectiveness of post-partum physician assisted self-management of blood pressure vs standard care over the first 6 (up to 12) months post-partum.

On March 17th 2020, the COVID-19 pandemic meant that all research across Oxford University and OUH NHS Foundation Trust had to be halted. Minor amendment 2.0 allowed us to continue follow up of those women already recruited but as of 06/05/2020 and Minor amendment allowed the POP-HT RCT[1] to restart during the COVID-19 pandemic. The investigating team believe it fulfils ‘exception 3’ of the OUH NHS Foundation Trust guidance for research during COVID-19 as the OUH TMA agreed and gave permission for the trial to restart in June 2020. Based on our experience during the follow up of the 18 participants already recruited, it is believed that ongoing recruitment into this trial will allow women in both the intervention and control arm to receive ‘better’ clinical care at a time of huge disruption to normal care. We also believe that this trial will allow the trust to better adhere to the updated Royal College of Obstetrics and Gynaecology (RCOG) guidance on the care of women during COVID-19.

A few key changes are required to the baseline visit to reduce the risk of COVID-19 transmission for both researchers and the study participants. Appendix F contains a full explanation and rationale for restarting the trial, which has been approved by the RDM Head of Department and submitted to OUH NHS Foundation trust R&D for their approval.

Main trial setting and participant profile

We aimed to recruit 200 participants with 1:1 randomisation. The intervention arm will comprise telemonitored home blood pressure monitoring (including periods of home 24hr ABPM) coupled with physician-assisted self-management. The control arm will receive ‘standard’ levels of NHS care from their GP and midwives and health visitors. All participants will be recruited from the Oxford Women’s Centre at the John Radcliffe Hospital, which sees approximately 25 patients per month with hypertensive pregnancies (demonstrated in a local audit). As a result of COVID-19 an updated power calculation was performed, to assess the possible dilutionary affect that the RCOG guidance on home BP monitoring during the 1st week post discharge could have on the primary trial outcome. Based on this it was decided by the Trial Steering Committee to increase the sample size to 220.

Main study visit and measures

A flow chart of the proposed study visits is seen in Appendix A.

The expected duration of participant involvement will be 6 (up to 12) months from enrolment to study completion and participants will be asked to attend four study visits after their pre-screening and enrolment: baseline, at 1 week, at 6 weeks and at 6-12 months. At times when face to face appointments are restricted due to regulations related to the COVID-19 pandemic, the week 1 and week 6 visits can be done remotely where

needed to avoid putting participants at undue risk. The primary outcome will be studied over a narrower time frame of 6-9 months as originally described at the studies outset, even during the COVID-19 pandemic, but a further amendment was approved to allow all BP based primary and secondary outcomes to be done remotely if needed. The other secondary outcomes can then be done over a wider timeframe of 6-12 months to allow participants to safely attend their study visit at the hospital (see table A1 of supplementary material). An optional sub-study involving administration of Gadolinium contrast will be offered as an additional component to the MRI during the final study visit in those women who are not breast feeding. This is part of exploratory work that may feed into a larger future trial.

Although this study only involved the four visits described above (outlined in appendix A), further contact may be planned to take place over the next 10 years to allow longitudinal follow up of this cohort's blood pressure. Details of any additional follow-up visits will be defined closer to the time, REC approval will be sought for any further amendments to allow additional visits, and additional consent will be sought from the participants to ensure they are happy to continue taking part.

The study team has used the experiences of patients participating in prior self-management studies to assist with this study design and methodology. This included consulting participants from other related studies done by this group, as well as consulting with some of the investigators from these studies. The participant information sheet, additional information sheet and intervention arm information sheet haves also been reviewed by several PPI members who have, or have had, raised blood pressure in pregnancy and it was duly amended in light of their comments.

Blood validation sub-study

We also aimed to recruit 20 normotensive participants as a reference population for endothelial function. These participants will be recruited from the Oxford Women's Centre at the John Radcliffe Hospital. The expected duration of participant involvement will be 6 (up to 12) months from enrolment to study completion and will involve 2 study visits following pre-screening and enrolment. The visits will be identical and include anthropometry, blood pressure measurements and a blood test.

8. Participant identification

Main trial participants

All participants will be females of childbearing age 18 years or over.

Inclusion in the trial will require a clinician confirmed diagnosis of either gestational hypertension or preeclampsia defined by NICE NG 133 [14] which requires anti-hypertensive medication to control.

Gestational hypertension diagnosis requires a BP >140mmHg systolic or > 90mnHg diastolic on >2 occasions after 20 weeks of this pregnancy.

Pre-eclampsia diagnosis will be defined as per NICE NG 133 [14]:

- New onset of hypertension (>140 mmHg systolic or over 90 mmHg diastolic) after 20 weeks of pregnancy and the coexistence of 1 or more of the following new onset conditions:
- Proteinuria (spot urine protein/creatinine over 30 mg/mmol [0.3 mg/mg] or over 300 mg/day or at least 1 g/L ['2 +'] on dipstick testing) or;
- Other maternal organ dysfunction:
    - Renal insufficiency (creatinine 90 umol/L or more, 1.02 mg/dL); o Liver involvement (elevated transaminases [ALT or AST over 40 IU/L] with or without right upper quadrant or epigastric abdominal pain);
    - Neurological complications such as eclampsia, altered mental status, blindness, stroke, clonus, severe headaches or persistent visual scotomata;
    - Haematological complications such as thrombocytopenia (platelet count below 150,000 cells/µL), disseminated intravascular coagulation (DIC) or haemolysis;

- Utero-placental dysfunction such as fetal growth restriction, abnormal umbilical artery Doppler waveform analysis, or stillbirth.

Participants fulfilling the above diagnoses will be screened against the inclusion and exclusion criteria below and enrolled if suitable.

Inclusion criteria

- Participant is willing and able to give informed consent for participation in the trial.
- Female, aged 18 years or above.
- Clinician confirmed diagnosis of either gestational hypertension or pre-eclampsia defined by NICE (as above).
- Requiring anti-hypertensive medication at the point of discharge from secondary care.
- Participant has clinically acceptable laboratory results and clinical course post-partum with no other adverse complicating factor requiring prolonged admission post-partum that would make participation unfeasible as judged by the CI. Examples would include stroke sequalae, ongoing DIC etc.
- In the Investigator's opinion, is able and willing to comply with all trial requirements including ownership of a 'Smart-phone/Tablet' and willing to use the smart-phone app if randomised to that arm.
- Sufficient competence in English Language to follow the app instructions and partake in the study, as judged by the CI.

Exclusion Criteria (the participant may not enter the trial if ANY of the following apply):

- Significant renal or hepatic impairment that would affect safe medication titration and adjustment as part of the trial, as deemed by the Investigator.
- Scheduled elective surgery (excluding caesarean sections) or other procedures requiring general anaesthesia during the trial.
- Participant with life expectancy of less than 6 months.
- Any other significant disease or disorder, which, in the opinion of the Investigator, may either, put the participants at risk because of participation in the trial, or may influence the result of the trial, or the participant's ability to participate in the trial.
- Participants who have participated in another research trial involving an investigational product in the past 12 weeks.
- An absolute contra-indication to MRI (as per MRI safety questionnaire) precludes the women from having an MRI but they can still participate in the remainder of the study
- Women with pre-existing hypertension will be excluded, as this is a separate pathology that would affect the efficacy of the study intervention and affect the primary and secondary outcomes of the study.

Additional exclusion criteria specific to the Gadolinium sub-study are: •
Breast feeding (at time of CMR scan),

- eGFR <30 ml/minute.

Blood validation sub-study

A sub- cohort of normotensive postnatal women will be recruited locally from the postnatal ward in the Oxford Women's Centre at the John Radcliffe Hospital. 20 normotensive participants will be recruited directly to the sub-study and will not be expected to participate in the main POP-HT study. 20/200 of the main trial participants will also have an additional blood test during their baseline visit as part of the sub study (see section 9.5 for further detail).

Inclusion Criteria for POP-HT blood validation sub-study

- Participant is willing and able to give informed consent for participation in the trial.
- Female, aged 18 years or above.
- Normotensive (BP 140/90) throughout antenatal and postnatal period

Exclusion Criteria for POP-HT blood validation sub-study

- Hypertensive disorder of pregnancy
- Use of beta blockers such as atenolol or equivalent
- BMI>35
- Evidence of cardiomyopathy, inherited cardiac conduction abnormalities, congenital heart disease or significant chronic disease relevant to cardiovascular status • Folic acid or folate supplementation in the third trimester

9. Protocol procedures

Study procedures will initially take place in the Women's centre of the John Radcliffe hospital in Oxford. The procedures for each study visit and the estimated time each will take are listed in appendices B and D.

The baseline study visit (V1) will be within the maternity care areas. Visits 2 and 3, at weeks 1 and 6 respectively, will take place at home, if preferred, or in CCRF at the John Radcliffe hospital, Oxford, depending on participant choice to make things easier for participants at a busy time in their lives. The final study visit at 6 (up to 9 months) will take place in CCRF and OCMR at the John Radcliffe hospital, Oxford. During all home visits, a female chaperone will be made available and wherever possible all echo scanning will be done by female researchers. All visits can be interrupted as needed for mothers to attend to their baby/babies. This option may not be available during exceptional circumstances, including the COVID-19 pandemic. See appendix F for more information.

There are essential and desirable procedures forming part of each study visit (see figure 2) so if time does become an issue, particularly during the longer final study visit, the visit can be shortened by not performing some of the desirable procedures. Although we would like all procedures performed in all visits, some of the secondary outcomes are exploratory and will be used to plan larger validation studies in future and hence the reason they are classed as 'desirable'. The final study visit will conclude with the fitting of a 24 hr blood pressure monitor and week-long activity monitor (wrist worn accelerometer) which participants will then post back in pre-paid and self-addressed envelopes to CCRF.

Recruitment

For the main study:

All trial participants will be recruited locally from the Oxford Women's Centre in the John Radcliffe Hospital, Oxford. Screening of notes and an eligibility check can take place in the antenatal period from twenty weeks gestation, when the diagnosis of pre-eclampsia/gestational hypertension can be made as per the NICE NG133[14] definition, up-to the time of discharge post-natally whilst they are receiving clinical care in the Oxford Women's Centre. Screening will be done by the clinical care team looking after the patient.

Following identification of a potentially eligible patient by the clinical team, a member of the clinical team will provide potential participants with an invitation letter, a participant invitation sheet (PIS) and the additional study information sheet, the latter of which details more about the procedures involved in the longer, final study visit. To avoid the use of paper during the COVID-19 pandemic these will be provided to the participant by the clinical team on a sterile tablet during this period of time and e-mailed to the participant also.

The clinical team will then follow up by obtaining verbal consent to inform the research team of the potential participant's interest. If verbal consent is given, the research team will then be notified. If this is antenatally, the research team will make contact after at least 24 hours. If this is postnatally, the research team will make contact after>12 hours. When the research team meet the participants, the women will be able to ask questions and talk through any queries with the study team before enrolment is considered. If they remain happy to take part, their medical notes will be screened again by a member of the research team to ensure they are definitely

eligible to participate. Antenatally, if a woman fulfils all of the eligibility criteria, the research team will check when the participant will next be visiting for clinical assessment and will aim to obtain written informed consent at that next visit. This will allow at least 24 hours for participants who are antenatal to consider the PIS and invitation letter prior to being consented.

In exceptional circumstances (including during the COVID-19 pandemic) participants may be given a reduced amount of time to consider participating in the study. Due to expedited discharge processes during the COVID-19 a reduced amount of time (at least an hour) will be given to consider the documentation whilst the pandemic is ongoing. Only a single member of the research team will be admitted to the maternity care areas, wearing level 1 personal protective equipment (PPE). More details of this can be found in Appendix F.

Posters and flyers will also be displayed in the Women's centre with the research team's contact details to allow screening for eligibility and provision of PIS, invitation letter and additional information sheet if participant's contact us in this way.

Whilst the study will aim to first approach women antenatally, it may be that they can only be enrolled postnatally in some cases e.g. if they present with pre-eclampsia as an emergency and have to have an emergency delivery, which is not uncommon. In these cases, again the clinical team will provide the aforementioned study documents and obtain verbal consent to refer the women to the research team. Due to the time limitation on their inpatient stay, a reduced period will be given for consideration of the study documents. In this case, a minimum of 12 hours will be given before approaching the potential participant to consider enrolment and consent into the study by the research team. This is to maximise potential enrolment to the study. It will be made clear to the potential participant that they are able to withdraw at any time from the study without giving any reason for withdrawal and without this affecting their clinical care.

<u>For the blood validation sub-study:</u>

Screening for eligibility will be performed by the clinical care team looking after the patient. Following identification of a potentially eligible patient by the clinical team, a member of the clinical team will provide potential participants with an invitation letter and a participant invitation sheet (PIS).

Records screening and eligibility assessment

Records screening may occur as early as 20 weeks' gestation once a hypertensive disorder of pregnancy has been diagnosed. Obstetric records will be screened by the NHS clinical care team to assess eligibility before the potential participant is considered for referral to the research team for possible recruitment.

If the participant has been screened and enrolled antenatally, the research team will re-screen postnatally to check that they still fulfil the eligibility criteria for the study i.e. That there have been no serious medical complications for the mother or the new-born in the intervening period that precludes their participation and that they still require anti-hypertensive medication postnatally. The team do not intend to deliberately exclude women based on any medical complications but wish to be sensitive to the needs of the participant and avoid overburdening them. It is also vital that women recruited antenatally are given a very clear opportunity to opt out at the postnatal stage in case they have changed their mind and hence it is planned to re-confirm their original consent at this stage.

Furthermore, patients who have previously not met the inclusion criteria initially may be rescreened later in pregnancy or in the postnatal period should their status change, e.g. if they have only just started on anti-hypertensive medication in the post-natal period. Women with raised blood pressure after birth typically stay in hospital for an average of 2-3 days after birth which should provide adequate opportunity for notes screening of newly identified eligible women, consent at >12 hours later (time reduced during COVID-19 as per protocol amendment –see table A1 of supplementary material) and then a baseline visit for those recruited postnatally. For those recruited antenatally, it will allow time for re-confirmation of eligibility and verbal re-confirmation of consent followed by the baseline visit. Protocol waivers will not be permitted.

Informed consent

The participant must personally sign and date the latest approved version of the Informed Consent form(s) before any trial specific procedures are performed. Please see Appendix F for details of how consent will be

modified during the COVID-19 pandemic to reduce risk of the paper acting as a vector for transmission of Coronavirus.

Written and verbal versions of the Participant Information and Informed Consent form(s) will be presented to the participants detailing no less than: the exact nature of the trial; what it will involve for the participant; the implications and constraints of the protocol; the known side effects and any risks involved in taking part. It will be clearly stated that the participant is free to withdraw from the trial at any time for any reason without prejudice to future care, without affecting their legal rights and with no obligation to give the reason for withdrawal.

Consent will be obtained at the earliest appropriate opportunity after provision of the PIS and invitation letter and discussion with the research team to enable any queries to be addressed. As explained in section 9.1, participants identified postnatally may be enrolled as early as 12 hours after provision of the PIS and invitation letter, but no earlier than this. These participants will have the same opportunity to ask questions. Only then after 12 hours, will the study team re-visit the participant to enrol them and obtain informed consent. For those recruited antenatally they will have the usual 24 hours minimum for consideration of the PIS and invitation letter before being asked to enrol.

Written Informed Consent will then be obtained by means of participant dated signature and dated signature of the person who presented and obtained the Informed Consent. This study will not involve children, vulnerable adults or adults who lack capacity. The person who obtained the consent must be suitably qualified and experienced, and have been authorised to do so by the Chief Investigator. A copy of the signed Informed Consent will be given to the participant. The original signed form will be placed in the medical notes and a further copy will be retained at the trial site and one given to the participant.

Additional informed consent will be sought at the final study visit/visit 4 for those participants eligible and willing to enter the gadolinium sub-study.

Randomisation

Randomisation will be performed as soon as possible following delivery of the baby/babies, after enrolment and consent, and after the baseline visit. The process of randomisation will involve secure webbased randomisation software (embedded within CASTOR ®) on a 1:1 basis and minimisation will be based on the following two factors:

- Primary factor agreed on as a surrogate marker of disease severity: Gestational age at the time of presentation with pre-eclampsia/gestational hypertension
- Receipt of prescription of ACE inhibitor (Enalapril) at time of randomisation.

Randomisation will be carried out (the generation and concealment of the random sequence) by CASTOR® EDC® proprietary web-based randomisation software. Prior to COVID-19 this was done by a separate member of the CCRF research group, to those investigators performing the baseline visit itself and a un-blinded research assistant will implement this (inform participants) and subsequently instruct on the use of the intervention for those randomised to that arm. However, since the onset of the COVID-19 pandemic: whilst restrictions on visitors remain in place for maternity areas, the randomisation will be done electronically via CASTOR® as before but the same individual doing the visit will inform participants of the arm they are allocated to and instruct them appropriately i.e. the trial changed from blinded to open-label (see blinding section below).

Prior to COVID, the clinical research manager/research assistant, involved in generating and implementing the randomisation sequence respectively, was not involved in primary or secondary outcome datacollection or analysis at any point. The clinical research manager was also responsible for allocation concealment. Allocation will be concealed using CASTOR EDC® electronic database. After restarting the trial post COVID, allocation was concealed using CASTOR EDC® from those performing the data analysis.

Both pre and post-COVID Study clinicians giving advice on the telephone help-line based on readings submitted to the app as part of the intervention will only see a number (e.g. POP-HT 004) and initials and there will be no face-to-face communication. To ensure adequate clinical decision-making a limited amount of

relevant medical history will be entered onto the POP-HT web-based system for clinicians logging on e.g. Pre-eclampsia onset at 32 weeks. EMCS. Haemoglobin 72 at discharge following postpartum haemorrhage, Creatinine 72. No other history of note. To minimise any potential risks from poor blood pressure control women will receive clear verbal advice and written guidance (in the form of intervention arm information sheet), and the same advice will be available on the app, regarding when to seek urgent medical help. This will be reinforced by messages from the tele-monitoring service when participants submit an out-of-range blood pressure reading.

Blinding and code-breaking

Due to the nature of the intervention, participants cannot be blinded. To reduce potential bias in the investigating team, the study team members performing the baseline and subsequent visits were planned to be blinded to the randomisation and this took place prior to the COVID-19 pandemic with randomisation and allocation occurring as outlined in the above section. However, as a result of COVID-19 there was a change in the OUH trust visitor policy and this prompted a change to the randomisation process, as detailed above, so it became impossible to maintain blinding. The study thus became a PROBE trial. However to maintain the trial's fidelity, as per discussion in the TSC/DMEC the outcome data will be analysed by members of the study team blinded to randomisation with results being verified by a second research team member.

Assessments

A flow chart of the proposed study visits is seen on the page below (figure 2) with subsequent explanation of each of the components in each visits in the text that follows. During the baseline visit, the blinded members of the research team will perform all procedures for participants in both arms i.e. the intervention and the control

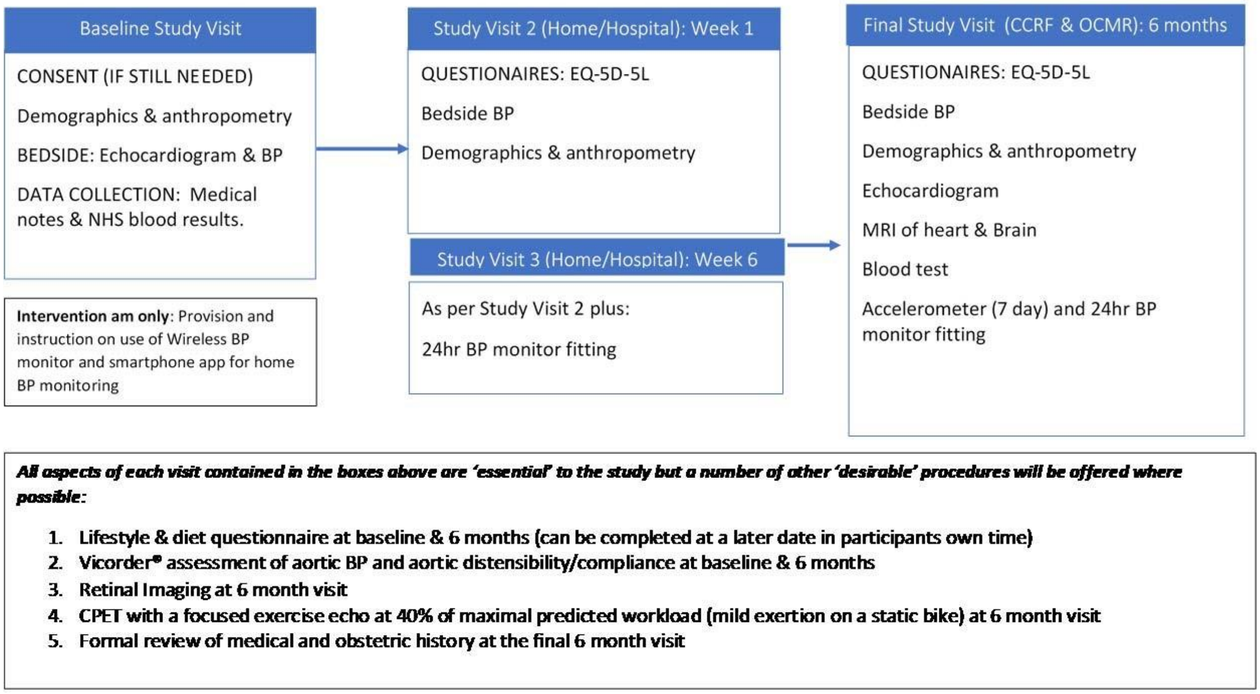

arm. The provision of the study intervention is for the intervention arm only. However, a blood pressure monitor will be provided to the control arm during the COVID-19 pandemic, as explained in more below.

Figure 2: Flow Chart of Proposed Study visits

As the diagram above illustrates, there will be ‘essential’ components in each study visit but as the study team is aware they will be doing the research on women with new-born babies, so some components have been classed as ‘desirable’ to allow for shortening of the study visits if required in some cases, in line with feedback from our PPI sessions. Although we would like all procedures performed in all cases in an ideal world, some of the secondary outcomes are exploratory and will be used to plan larger validation studies in future; hence the reason they are classed as ‘desirable’. Each of the assessments can be interrupted and restarted as needed in a flexible manner should the need to attend the baby/babies arise during the visit.

During the COVID-19 pandemic, the baseline visit was adjusted (as described in Appendix F). This was implemented to reduce the length of time of direct patient contact to just the echo, and to make the Vicorder® test optional. All contact will be done in appropriate PPE for which Dr Kitt is a trainer as well as being well practiced from clinical work during the pandemic.

Baseline visit (week 0):

Demographics and anthropometry:
Assessment will include recording of the antenatal booking height, weight and BMI (obtained from notes) and the mid-left arm circumference.

Bed-side Blood pressure measurement:
Participants will have their blood pressure checked after 5 minutes’ rest using the automated mode of a validated sphygmomanometer. Three blood pressure readings will be taken at intervals of 1 minute. The first reading will be discarded and not used toward the mean blood pressure calculated for the visit. As previously done in SNAP-HT [4], measurements will be done on the left arm using automated DINAMapV100® blood pressure monitors, ensuring the appropriate cuff size for the arm circumference. The measurement technique advised by the British Heart Foundation and NICE NG133 will be strictly followed i.e. participant will be sat/laying, advised not to talk at the time of measurement and, the arm will be supported and elevated to the height of the heart, with the legs uncrossed.

Echocardiogram (cardiac ultrasound) scan:
Cardiac ultrasound imaging will be performed by a trained sonographer to evaluate cardiac structure and function. Resting transthoracic echocardiography will be performed in the left lateral decubitus position using a commercially available Philips CX50, Philips EPIQ 7C, Philips IE33 or equivalent cardiology ultrasound machine. British Society of Echocardiography guidelines will be followed for collection of a standard clinical imaging dataset

Collate data from medical notes and review blood results:
This part of the study visit will not require participant involvement/participation. A study team member will review the medical notes (paper and electronic) to document the following information: Gestation at diagnosis, gestation at delivery, the numbers of days of antenatal blood pressure treatment, the mode of delivery, the number and doses of anti-hypertensive drugs used post-natally, whether ACE inhibitors are prescribe or not at the time of enrolment, the baby (babies) weight(s) and, the presence of IUGR (defined as AC<40%). The team will also record the day 1 postnatal blood results, the urine PCR at its peak and the sFLT/PIGF ratio is measured, all medications prescribed at the time of enrolment and, any other medical and obstetric history of note.

Quality of Life questionnaire (EQ-5D-5L):
Participants will be provided with an EQ-5D-5L questionnaire via e-mail and a trained study investigator will run through the structured questionnaire during the visit with the participant. If required, there will be an option to complete the questionnaires later

Desirable: Vicorder® (Vascular Measures and Central Blood Pressures):
Resting measures of vascular stiffness including pulse wave velocity and central blood pressure will be collected using a non-invasive device (Vicorder®).

Desirable: Lifestyle and diet questionnaire:
Study visit participants will be asked to complete a study questionnaire. The questionnaire combines validated questions piloted or used in previous studies. Information will be collected on factors that affect blood pressure including: smoking frequency, alcohol and salt intake, exercise and family history. This is being done via e-mail to avoid all paper use during COVID-19, and participants can complete this at a later date in their own time.

Desirable: Blood test (10 minutes):
A venous blood sample (approximately 25mls) will be taken at rest and include samples for analysis of biomarkers associated with inflammation, angiogenesis and endothelial activation as well as endothelial colony forming cells (ECFCs) in 20/200 partitiicipants who enter the endothelical cell sub-study. Blood tests will be taken, where possible, at the same time as clinically indicated venepuncture.

Intervention provision: Automated blood pressure cuff provided and POP-HT app installed (those randomised to intervention arm only):
At the end of the baseline visit, those individuals that randomised to the intervention arm will be issued an automated blood pressure cuff (OMRON EVOLV®), validated in pregnancy [30]. They will also download the POP-HT smart-phone app and be taught how to use the cuff and app. The participant will then have the remainder of their stay in hospital to practice and will be contacted remotely via tablet if there are any problems. This is to ensure all parties are confident and competent prior to discharge home at which point the intervention will start. A telephone number (9-5pm Monday-Friday) and e-mail address will be provided for any technical problems.

Control arm during COVID-19:
During the COVID-19 pandemic, section 3.1.5 of the RCOG Guidance for maternal medicine in the evolving coronavirus (COVID-19) pandemic, published in July 2020 recommends 'self-monitoring 2-3 times in the first week after discharge for women who have had a hypertensive pregnancy. Therefore, those women allocated to the control arm, who are unable to obtain a monitor from the Oxford Women's centre/NHS service, will be provided with a validated BP home-monitoring device by the trial team to ensure they can adhere to this RCOG guidance during week 1. These monitors will be provided to enable the control arm participants to monitor their own blood pressure and in turn liaise with their own GP/midwife to adjust their management based on their readings. The study team will not be offering remote management to the control arm or providing them with an app, or interpretation of the readings and management decisions are to be taken by their own GPs/clinicians. These monitors will also be used to allow remote BP measurements during the study 'visits' at week 1 and 6. They will be returned via the post to the POP-HT team after the 6 weeks visit and duly sterilised.

Subsequent visits

During Visits 2 and 3, the research team will perform all procedures for participants in both arms.

Visits 2 and 3:

At visits 2 and 3 at weeks 1 and week 6 (+/- 5 days) post-discharge there will measure blood pressure three times as per the baseline visits, update the demographics and anthropometry, and complete an ED-5D-5L questionnaire. At the end of visit 3, the team will also fit a 24hr blood pressure monitor. These two visits will take place at weeks 1 and 6 post-discharge respectively. They will be offered as a visit to our research facility (CCRF) or a home visit to minimise the trial burden at this busy period in these women's lives. During all home visits, a female chaperone will be made available. We will allow a 5-day window of flexibility for the week 6 visit but cannot allow any longer than 5 days as it may affect the validity of the data. In extenuating circumstances such as COVID-19 all aspects of Visits 2 and 3 will be conducted remotely via video (and/or phone call).

Demographic and anthropometry:
Height, weight, mid-left arm and waist and hip circumference will be performed by the research team for those visits done in person. If done remotely, weight will be obtained using a set of home scales and midleft arm circumference will be measured from the left arm at rest. The measurement will be done by the participant/family member under direct video supervision/guidance to ensure consistency. Waist and hip will not be recorded for remote visits at week 6.

Blood pressure measurement:
Assessment will be performed as outline above for baseline visit. During COVID-19 the readings will be done under direct video supervision using the same BHF gold-standard approach described above. 3 blood pressure measurements will be obtained at 1-minute intervals. The values will be shown on screen and then recorded by the study team.

Quality of Life questionnaire (EQ-5D-5L):
Participants will be provided with an EQ-5D-5L questionnaire via e-mail and a trained study investigator will run through the structured questionnaire during the visit with the participant. If required, there will be an option to complete the questionnaires later.

Visit 3 only: 24hr ambulatory blood pressure monitor (to be worn for 24-hour period)

24-hour ambulatory blood pressure monitoring will be initiated at the end of the study visit using validated, calibrated, automated oscillometric, ambulatory devices (SPACELABS® 90217 or equivalent). Correct cuff size will be chosen based on arm circumference recorded at week 1 and 6. Subjects will be instructed to remain still during measurements. Measurements will be automatically taken every 30 minutes during daytime and then hourly nocturnally from 22:00 PM to 7:00AM. Subjects will complete a diary documenting hours asleep and awake and any exertion that could inflate a reading artificially. A member of the study team will fit the ambulatory blood pressure monitor for those visits done ‘in person’ but, during COVID-19 the 24hr cuff, diary and monitor will be posted to participants and the fitting will be performed via video call by trained, experienced study investigators. An adequate cuff fit will be checked by performing one manual reading, which is then excluded from data analysis. The participants will then be asked to post the equipment back to CCRF in a pre-paid, pre-addressed envelope where adequate sterilisation and quarantine of the equipment will be performed prior to data upload. ABP data will be verified by a trained study investigator.

Visit 4 (6 months (up to 12 months) post-partum

Participants will be invited to a final study visit at CCRF and/or OCMR. This is the longest and most comprehensive visit but once again, there are essential and desirable components, which means the visit, can be shortened if required. A female chaperone will be offered if desired and where possible all echocardiography will be performed by a female sonographer. In extenuating circumstances, such as COVID-19 national lockdowns, where participants do not wish to attend in person, or where our facilities is redeployed as a COVID escalation areas; the 24hr BP monitoring and the procedures below can be conducted remotely via video call. Otherwise, these will be performed in person as described for the baseline visit.

Demographics and anthropometry:
Assessment will be performed as outlined above

Blood pressure measurement:
Assessment will be performed as outlined above

Quality of Life questionnaire (EQ-5D-5L):
This will be e-mailed to the participant as outlined above.

Fitting of a home blood pressure monitor:
Assessment will be performed as outlined above for visit 3

Fitting of an activity monitor:
For remote video calls, the accelerometer will be pre-programmed based on participant reported height, weight and hand dominance and then posted to the participant and they will be shown how to fit it via video call. This consists of an activity monitor placed inside a rubber wristband. It is shock and waterproof so participants can shower/swim with it on. Wrist-worn accelerometers have high compliance and reliability and are validated measures of physical activity. Participants will be asked to wear the accelerometer for 7 days, 24 hours per day and a 7 day interval will be pre-programmed to allow for postage. A stamped addressed envelope will be provided to return the device after use. Participants will be asked to perform whatever activities they would

normally do, whilst wearing the accelerometer, to try and accurately reflect their normal levels of daily activity. For visits done as normal, the accelerometer will be programmed during the visit, based on their height and weight recorded as part of the study visit and, fitted to their non-dominant wrist.

Review of medical and obstetric history and any medication side effects:
The study investigator will record any side effects to current/previous medication including blood pressure medication as well as record any medical problems they have developed since discharge from hospital. This information will be obtained from the participant and corroborated with the medical notes as required. If the participant does not want to be involved in this aspect due to time constraints, the medical notes alone will be used to obtain this information. In cases where the above measures are performed remotely the following procedures, which cannot be done at remotely will be scheduled as soon as possible after, and within the 12month time window defined in this protocol, when the participant feels safe to come to a hospital site again. For all other participants, all procedures will be done during a single study visit.

Echocardiogram cardiac ultrasound scan:
Assessment will be performed as outlined above for baseline visit.

Vicorder® (Vascular Measures and Central Blood Pressure):
Assessment will be performed as outlined above for the baseline visit. The procedure itself will be done during the MRI scan in order that the central/aortic pressure obtained by the Vicorder can be correlated with the aortic MRI cine images we acquire.

Retinal imaging:
Retinal photography of the right eye (3 single shot images centred on the optic disc) will be completed using a digital camera and imaging software following an established protocol. Imaging is non-invasive with no requirement for topical drops and does not require removal of contact lenses. These images are deidentified (known only by the study ID) and will be stored on our secure high compliance server for up to 10 years.

MRI:
A 3Tesla (3T) Siemens PRISMA scanner will be used to quantify brain structure and volume, followed by cardiac structure and function, cardiac mapping, measurement of aortic distensibility; and T1 maps of the kidneys.

Gadolinium contrast (optional):
Gadolinium will be offered as an additional optional component to the MRI to those women who are not breast feeding, as part of exploratory work that may feed into a larger future trial. A call will be made to clarify this 2-3 weeks prior to this visit and the additional PIS explains the Gadolinium procedure and risks/benefits in more detail (as detailed in section 17.4). Separate informed consent will be obtained for those women who wish to participate in this aspect of the study prior to the MRI being performed. Before any gadolinium is administered, the last creatinine value taken prior to discharge post-partum (up to 9 months prior) will be reviewed and if normal, a repeat will not be needed. If not available or if elevated from that time or clinical judgement deems it necessary, a repeat finger prick creatinine will be done. If the reading demonstrates an eGFR<60mls/min, the scan will not take place and the procedure for incidental abnormal clinical findings will be followed.

Blood:
A venous blood sample (approximately 25mls) will be taken at rest and include samples for a) whole blood, plasma and serum lipid and inflammatory marker analysis and b) analysis of biochemistry and metabolism and, if women consent to late gadolinium imaging as part of their Cardiac MRI scan, the blood sample will be obtained during cannula insertion in OCMR and an additional vial of blood will be collected to measure serum haematocrit.

Cardiopulmonary Exercise Testing with exercise echo (30 minutes): Desirable
Cardiac function and oxygen requirements in response to an incremental increase in workload will be measured via a cardiopulmonary exercise test (CPET). The exercise protocol is a validated incremental protocol with established use in clinical and research practice. The exercise protocol is currently utilised in ongoing ethically approved studies conducted by the Division of Cardiovascular Medicine, and is performed on a stationary bike.

The test commences with resting measures of spirometry. Participants will then exercise with an incrementally increasing workload (increasing resistance to pedal against) up to 4060% of their estimated peak exercise capacity. During the test, heart rate will be recorded using ECG monitoring, blood pressure will be measured at intervals and participants will report effort using the Borg exertion scale and the oxygen consumption and CO2 production will be measured using a calibrated mask. It is a non-invasive, painless technique with no associated risks. An additional brief (2-3 minute) focused echo will be performed at 40% of their maximal predicted exercise intensity (whilst on the bike) to enable measurement of exercise ejection fraction. A female sonographer will be provided wherever possible.

Follow-up of participant medical records:
Participants will be asked to consent to be followed- for up to 10 years. The research team in Oxford will ask for information from NHS Digital. They will send the participants' name, date of birth, NHS number and postcode to NHS Digital (or other central NHS bodies) who can link this information to centrally held records. The team may also want to assess the blood pressure of the participants again in the future (up to 10 years from enrolment) as part of a further study visit but this will be submitted as an amendment/extension to this study prior to the study terminating. Participants are not compelled to take part in any extension study by participating in this initial part of this study.

Sub- study visits: circulating biomarker validation and evaluation

An additional cohort of participants (n=20 normotensive participants) will be invited to the blood validation and evaluation study only. 20/200 of the hypertensive pregnancy patients from the main trial will also have a blood test taken at baseline as part of their main study visit, for which additional written, informed consent will be sought. For the normotensive participants, the following study procedures and visits listed below remain separate to the main study. Study procedures will be the same during both visits.
The baseline visit will be carried out on the postnatal ward, in the Women's Centre, prior to discharge. Normotensive participants will be invited back for a 2nd visit in CCRF/OCMR in the John Radcliffe Hospital between 6-12 months post-partum, and for those 20/200 in the main trial, the repeat blood test for the sub-study will be performed during the main V4/final visit when they have the other routine blood test performed.

<u>Procedures for all sub-study patients are:</u>

Baseline:

Demographics and anthropometry (10 minutes):
Assessment will include height (obtained from notes), measuring post-natal weight and BMI. Demographic details will be recorded and cross-referenced to the notes.

Bed-side Blood pressure measurement (10 minutes):
Participants will have their blood pressure checked after 5 minutes' rest using the automated mode of a validated sphygmomanometer. Three blood pressure readings will be taken at intervals of 1 minute. The first reading will be discarded and not used toward the mean blood pressure calculated for the visit. The measurements will be done on the left arm using automated DINAMapV100® blood pressure monitors. The measurement technique advised by the British Heart Foundation will be strictly followed as in the original study i.e. participant will be sat, advised not to talk at the time of measurement and, the arm will be supported and elevated to the height of the heart with the legs uncrossed.

Blood test (10 minutes):
A venous blood sample (approximately 25mls) will be taken at rest and include samples for analysis of biomarkers associated with inflammation, angiogenesis and endothelial activation as well as endothelial colony forming cells (ECFCs) Blood tests will be taken, where possible, at the same time as clinically indicated venepuncture.

Visit 2:
Participants will be invited back, between 6-12 months postnatally. Study procedures will be as per listed at baseline. This study visit will take place in CCRF.

Sample handling for trial purposes

Blood samples will be centrifuged and the whole blood plasma and serum will be separated and stored. All samples will be retained in a secure environment for future analysis and will be stored in a de-identified format at the University of Oxford under the custodianship of the Division of Cardiovascular Medicine. The samples will be stored for at least 10 years and may be used in future ethically approved studies as our understanding of blood vessel function grows. A copy of the consent will be retained from participants who consented for their samples to be used in future research. All other human materials will be disposed of at the end of the study in accordance with the Human Tissue Authority Code of Practice.

Early Discontinuation/Withdrawal of Participants

During the course of the trial, a participant may choose to withdraw early from the trial treatment at any time. This may happen for a number of reasons, including but not limited to:

- The occurrence of what the participant perceives as an intolerable AE (if this is the reason the participant must undergo an end of trial assessment and be given appropriate care under medical supervision until symptoms cease, or the condition becomes stable)
- Inability to comply with trial procedures
- Participant decision

Participants may choose to stop treatment and/or study assessments but may remain on study follow-up. Participants may also withdraw their consent, meaning that they wish to withdraw from the study completely. Participants may have the following two options for withdrawal and these will be explained to them should the situation arise:

- Participants can withdraw from the study but permit data and samples obtained up until the point of withdrawal to be retained for use in the study analysis. No further data or samples would be collected after withdrawal.
- Participants can withdraw completely from the study and withdraw the data and samples collected up until the point of withdrawal. The data and samples already collected would not be used in the final study analysis except where analysis of their data or samples has already been integrated into interim results and this will be explained in the participant information sheet.

Data collected from participants who wish to withdraw consent from the study will be included in the analyses up to the point of withdrawal of consent unless explicitly stated by the participant that this is against their wish. The participant will not contribute further data to the study and the CI will be informed. Participants do not need to provide any reason for withdrawal; should they freely offer a reason it will be recorded in the CRF. The research team may recruit additional participants to replace participants who have withdrawn.
In addition, the Investigator may discontinue a participant from the trial treatment at any time if the Investigator considers it necessary for any reason including, but not limited to:

- Pregnancy
- Ineligibility (either arising during the trial or retrospectively having been overlooked at screening)
- Significant protocol deviation
- Significant non-compliance with treatment regimen or trial requirements
- An adverse event which requires discontinuation of the trial or results in inability to continue to comply with trial procedures

If the participant is withdrawn due to an adverse event, the Investigator will arrange for follow-up visits or telephone calls until the adverse event has resolved or stabilised. If a participant is withdrawn from treatment due to a further pregnancy the pregnancy will be followed-up to outcome. See the Safety Reporting section below.

Definition of end of trial

The end of trial is when the 10-year follow-up has been completed or when the last sample has been analysed, whichever is later.

10. Study intervention

See accompanying document

11. Safety reporting

Adverse Event Definitions

Serious Adverse Event (SAE)	A serious adverse event is any untoward medical occurrence that:<br>• Results in death<br>• Is life-threatening<br>• Requires inpatient hospitalisation or prolongation of existing hospitalisation • Results in persistent or significant disability/incapacity<br>• Consists of a congenital anomaly or birth defect.<br>Other 'important medical events' may also be considered a serious adverse event when, based upon appropriate medical judgement, the event may jeopardise the participant and may require medical or surgical intervention to prevent one of the outcomes listed above.<br>NOTE the term "life-threatening" in the definition of "serious" refers to an event in which the participant was at risk of death at the time of the event; it does not refer to an event, which hypothetically might have caused death if it were more severe.

Note: to avoid confusion or misunderstanding of the difference between the terms "serious" and "severe", the following note of clarification is provided: "Severe" is often used to describe intensity of a specific event, which may be of relatively minor medical significance. "Seriousness" is the regulatory definition supplied above.

Procedures for Reporting Adverse Events

A serious adverse event (SAE) occurring to a participant should be reported to the REC that gave a favourable opinion of the study where in the opinion of the Chief Investigator the event was 'related' (resulted from administration of any of the research procedures) and 'unexpected' in relation to those procedures. Reports of related and unexpected SAEs should be submitted within 15 working days of the Chief Investigator becoming aware of the event, using the HRA report of serious adverse event form (see HRA website).

The severity of events will be assessed on the following scale: 1 = mild, 2 = moderate, 3 = severe.

Non-serious AEs considered related to the trial intervention as judged by a medically qualified investigator or the Sponsor will be followed up once the event is considered stable. It will be left to the Investigator's clinical judgment to decide whether or not an AE is of sufficient severity to require the participant's removal from the trial. A participant may also voluntarily withdraw from the trial due to what he or she perceives as an intolerable AE. If either of these occurs, the participant must undergo an end of trial assessment and be given appropriate care under medical supervision until symptoms cease, or the condition becomes stable.

Events exempt from immediate reporting as SAEs

There are a number of expected admissions/consultations with healthcare providers that will be expected take place as part of the natural history of pre-eclampsia and gestational hypertension during the trial period. These will be classed as 'Foreseeable Events' exempt from reporting as SAEs and include:

- Severe hypertension;
- Maternal morbidity: TIA; blindness; inotropic support; pulmonary oedema; respiratory failure; SpO2 <90%; myocardial ischaemia or infarction; hepatic dysfunction, hepatic haematoma or rupture; acute kidney injury; or transfusion;
- Post-partum haemorrhage;
- Lower genital tract bleeding;
- Sepsis;
- Admission to hospital for pre-eclampsia, monitoring of hypertension, or symptoms of low blood pressure

- Pre-planned hospitalisation;
- Diagnostic and therapeutic procedures;
- Worsening pruritis;
- A pre-existing maternal condition (such as renal disease), unless it causes increased clinical concern;
- Admission for psychiatric or social reasons;
- Retained placenta;
- Extended hospital stay of the mother due to the need to keep her baby in hospital;
- Neonatal care unit admission for indications unrelated to pregnancy hypertension, such as neonatal hyperbilirubinaemia or unanticipated care for a fetal anomaly; or
- Fetal congenital anomaly

This list is not exhaustive and therefore any other 'minor medical significance symptom' as judged by the CI/PI, which does not require inpatient hospitalisation or prolongation of existing hospitalisation or result in persistent or significant disability/incapacity, is not life-threatening and, does not result in death will not be classed as an adverse event not an SAE.

12. Statistics

Statistical Analysis Plan (SAP)

The statistical aspects of the study are summarised here with details fully described in a statistical analysis plan included in a separate section of this supplementary material.

Description of Statistical Methods

The analysis will be carried out on the basis of intention-to-treat (ITT). This is, after randomisation, participants will be analysed according to their allocated intervention group irrespective of what treatment they actually receive. Patient demographic characteristics and other baseline information will be summarised by treatment group. Numbers (with percentages) for binary and categorical variables and mean (standard deviation), or median (interquartile or full range) for continuous variables will be presented. The analysis of the primary outcome will be assessed using a mixed effects model with baseline value, minimisation factors used in the randomisation process, randomised group and time will be fitted as fixed effects with a random intercept for each participant. Results will be presented as adjusted mean difference in change in mean ambulatory diastolic blood pressure between randomised groups at 6 months with 95% confidence intervals (CI) and associated two-sided p value. Secondary blood pressure outcomes will be analysed using the same method. Other secondary outcomes will be analysed using analysis of covariance (ANCOVA) to establish a co-variant model to examine the effect of blood pressure control in the postpartum period on cardiac structure and function, vascular function and cerebro-vascular structure and function. If the model assumptions are not met and evidence of departure from normality is observed, transformations of the data will be employed or non-parametric tests will be carried out.

Descriptive statistics (mean, standard deviation, standard error, range, etc.) will also be calculated for each outcome for each group. Differences in the primary and secondary outcomes will be compared between intervention and control groups.

Mean changes in blood pressures will be compared across the population and correlated with cardiovascular endpoints including cardiac structure and function reported from cardiac MRI and echocardiogram. Demographic and physiological characteristics of the participants will be added to regression models as covariates to explore the determinants of change in blood pressure comparing intervention and control groups.

Sample size determination

Power calculations to determine adequate sample sizes for this trial are summarised below:
Primary outcome measure: 24-hour average diastolic blood pressure (mmHg) at 6-9 months post-partum as assessed by SPACELAB 90217 24hr Ambulatory blood pressure monitor

Sample size calculation: The detection of BP differences between the 2 arms of this trial is based on the mean diastolic blood pressure difference detected in the pilot SNAP-HT study at 6 months. The mean BP difference detected between the intervention and control arm at the 6 month time-point was -4.5mmHg. We have used a more conservative standard deviation (SD) of 10mmHg in each arm (in SNAP-HT the SD was 8.2mmHg in the intervention arm and 9.8 mmHg in the standard care arm) and 10mmHg SD is in keeping with pooled SDs for ambulatory diastolic blood pressure readings from other studies. To detect a treatment effect on diastolic blood pressure of -4.5mmHg, powered to 80% at p=0.05 requires a total sample size of 158 and with 1:1 randomisation this would require 79 in each arm. We have adjusted our power calculations to determine the final sample size, to allow for up to 20% loss to follow up/withdrawal based on prior experience. Thus, we aim to recruit 100 to the intervention and 100 to the control arm.

Secondary outcome hypothesis: Improved blood pressure control in the post-partum period (0-9 months) in POP-HT will result in improved cardiac, vascular and cerebrovascular phenotypes at 6-12 months postpartum

Secondary outcome power calculations:

- Cardiac structure*:* Studies using echocardiography by our collaborators have compared BP and LV mass in pre-eclampsia patients and control patients, at 1-year post-partum [19, 31][19, 31][19, 31][19, 31][19, 31][19, 31]. They found that a difference in BP at 1 year of 10mmHg in diastolic BP corresponded to significant differences in LV mass. SNAP-HT appeared to achieve a 50% reduction of anticipated BP difference seen between pre-eclamptic and normotensives at 1 year by 6 months i.e. ~5mmHg. If it is assumed that the structural/phenotypic benefit results from the BP benefit, as we are hypothesising, then we must power to detect 50% of the phenotypic difference.
  In previous work by our group it has been demonstrated significant differences in LV mass/EDV (g/ml) in a similar age and predominantly female population with similar mean diastolic BP differences between groups to that seen in SNAP-HT. The LV mass/EDV (g/ml) in the group with high normal blood pressure was 1.54g/ml vs. 1.22 g/ml in those with optimal blood pressure with a standard deviation of 0.33 and 0.27 respectively at P<0.001. Based on these assumptions, to observe a treatment effect of 0.16 (50% of the difference between 1.54g/ml and 1.22g/ml) on LV mass/EDV, requires 67 in the intervention arm and 67 in the control arm (132 total). This is calculated using the larger SD of 0.33 referenced above at a power of >80% to detect a difference between the groups at p=0.05. This number should take into account for the greater dropout rate we may see for the MRI outcomes.

- Brain White matter integrity*:* Work by our group, on pre-eclamptic pregnancy, showed an increased burden of temporal lobe white matter lesion volume 5-10 years after a pre-eclamptic pregnancy (23.2±13 µl) vs matched individuals who had a normotensive pregnancy (10.9 ± 11.5 µl) at p<0.05.If we again assume we can detect a 50% of the phenotypic benefit with our intervention as outlined above, we would anticipate a 50% reduction in the burden of white matter lesions i.e. 6.15 µl (50% of 23.2 – 10.9 µl) in the intervention arm. With 71 in the intervention group and 71 in the control group (142 total), this will provide >80% power at p=0.05, even using the more conservative SD of 13ul to detect a 50% improvement in white matter lesion volume
  between the intervention and the control group. This number should take into account for the greater dropout rate we may see for the MRI outcomes.

- Aortic compliance: Several studies assessing the impact of blood pressure on aortic compliance have shown that even modest reductions in systolic/diastolic blood pressure increase aortic distensibility/compliance. One such study had a mean difference in systolic blood pressure of 4.6mmHg between the 2 drug treatment arms at 52 weeks, akin to the same mean difference in SNAP-HT at 6 months, albeit this was diastolic not systolic, although other studies have suggested diastolic BP may be even more important in influencing aortic compliance. In this study with a mean 4.6mmHg difference in systolic BP there was a treatment difference of 0.12 [(95% CI −0.35, 0.60), *P* = 0.60 in aortic compliance. Based on these assumptions, to observe a treatment effect from our intervention, with 100 in the intervention and 100 in the control arm we will be more than powered at >90% to detect a difference at P=0.05 in POP-HT.

- Exercise ejection fraction: Huckstep et al from our group compared resting and exercise ejection fractions for young adults with high normal BP vs. a normotensive cohort. The cohort was very well matched demographically to our planned study cohort, albeit it included both males and females. Resting ejection fraction (by Biplane Simpson's) was similar between groups but at 40%60% of peak exercise intensity, the higher blood pressure group had a lower exercise ejection fraction than the normotensive cohort (73.9±3.25 vs. 80.0±4.54%, $p<0.001$) and in keeping with this, a smaller increase in ejection fraction when going from baseline to 40% exercise intensity (10.4±5.92 vs. 19.0±6.90%, $p<0.001$). Assuming the ~5mmHg mean BP improvement achieved in SNAP-HT again translates to a 50% phenotypic benefit, when assessing exercise ejection fraction we anticipate a 4.3% improvement in exercise stress ejection fraction in the intervention arm vs. the control arm (4.3% is 50% of the difference i.e. 50% of 10.4-19%). With 43 participants in the intervention arm and 43 in the control arm (86 total) we will be powered at >80% to detect such a difference.

.

Analysis populations

The participants that will be included in the analysis will be all of those randomised. All data will be included in the analysis as far as possible to allow full ITT analysis, though there will inevitably be the problem of missing data due to withdrawal, loss to follow-up or non-completion of questionnaire data.

Decision points

There will be no formal interim analysis. The results once analysed will be reviewed by the research team, the Trial Steering Committee (TSC) and Data and Safety Monitoring Committee (DSMC) and the PI/CI and other collaborators.

The level of statistical significance

Level of significance will be tested as a 5% two-sided significant level.

Procedure for accounting for missing, unused, and spurious data

Missing data: Missing data will be reported with reasons given where available and the missing data pattern will be examined. We will explore the mechanism of missing data, though the mixed effects model implicitly accounts for data missing at random. The need for a sensitivity analysis taking into account missing data using multiple imputation will be considered. Spurious data will be assessed using standard editing criteria.

Procedures for reporting any deviation(s) from the original statistical plan

The final statistical plan will be agreed prior to final data lock and prior to any analyses taking place. Any deviation thereafter will be reported in the final trial report.

13. Data management

The data management aspects of the study are summarised here with further details fully described in the Data Management Plan.

Source data

Source documents are where data are first recorded, and from which participants' CRF data are obtained. These include, but are not limited to, paper and electronic hospital records (from which medical history and previous and concurrent medication may be summarised into the CRF), clinical charts and laboratory and pharmacy records. CRF entries will be considered source data if the CRF is the site of the original recording (e.g. there is no other written or electronic record of data). All documents will be stored safely in confidential conditions. On all trial-specific documents, other than the signed consent, the participant will be referred to by the trial participant number/code, not by name.

Access to Data

Direct access will be granted to authorised representatives from the Sponsor, host institution and the regulatory authorities to permit trial-related monitoring, audits and inspections.

Data Recording and Record Keeping

The study will comply with the General Data Protection Regulation (GDPR) and Data Protection Act 2018. The University of Oxford, as sponsor will act as data controller for the study.

Electronic documents that contain participants' personal identifying information such as the code-break document will be stored on the secure University of Oxford High Compliance system, a service for Clinical Trials Units (CTUs) and Medical Division Departments that securely accesses applications and stores sensitive data. This remote data capture system is fully compliant with ICH-GCP requirements. Personal data on paper documents such as informed consent forms will be stored securely in lockable cabinets with restricted access by authorised personnel only. The Chief/Principal investigator is responsible for keeping these documents encrypted where possible and to make sure that such documents are kept securely to ensure that in case of an emergency, participants can be identified and contacted. All source documents will have all identifiers removed and replaced by only the study ID number as soon as possible.

Study data will be recorded in a pseudo-anonymised manner and processed electronically where applicable and participants would not be identifiable from this. All participants will be identified by a code number (Study ID number) on case report forms and any electronic databases i.e. they will be de-identified. During the study, the hard copy data will be stored securely and at the end of the study, this will be archived in a secure archive location with restricted access.

Personal data (such as contact details and information that could identify a participant, except from those who have consented for future approach) will be destroyed as soon as it is practical to do so and no later than 12 months after the end of the study. Retinal images will be de-identified (known only by the study ID) and will be stored on our secure high compliance server for up to 10 years. The personal identifiers (name of participant) contained in consent forms and the code break document will be stored or accessed for up to 10 years after study end, after which time the custodian will agree a date for destruction and it will be destroyed confidentially.

Electronic data will be held on secure network drives/hard disks/servers on password-protected computers. Backup copies of files will be made regularly and stored on a different secure server/external hard drive. These back up locations will be subject to the same security principles as the primary locations. When datasets are complete, the primary copy will remain at the study site where it will be transferred onto optical media, e.g. DVD/external drives and undergoes archiving for hard copy data. Any copies leaving the study site will be de-identified.

All de-identified study data will in turn be entered onto a password protected, electronic cloud based software kept in England, called 'Castor EDC'. Castor EDC is an electronic data capture and management system that permits secure multi-site access. The server is based in the UK and it complies with relevant laws to ensure that the data is held securely. The participants will be identified by a unique study specific participant ID in this database also and will not be identifiable from this.

Information held and maintained by NHS Digital/ Office for National Statistics (ONS) may be used to provide information about health status and the NHS number, date of birth and postcode may be shared securely to obtain such information and allow contact for blood pressure, and other relevant measurements over the next 10 years.

The POP-HT software interfaces facing the participants (web-application, mobile app and SMS system) will display their first and last name and their phone number alongside the clinical data required to manage their post-partum hypertension through each system. This was shown in PPI meetings for this study and intervention testing for POP-HT and for BUMP (NCT03334149) to help establish trust with the software (i.e. they confirm more easily the data related to each of them). All of this information is deleted from the system within 12 months as per the other personal data discussed in the section above.

Researchers, obstetricians and midwives authorised to login into the patient management web-application will also see this data but will only see initials, study ID and contact numbers, not full names.

Beyond these interfaces, all patient data is associated with either their unique study ID, or with a random number generated by the system called a UUID. For example, any e-mails generated to the study team will only use the study ID to identify the participants. On the other hand, the web-application usability data (i.e. number of page-views, number of clicks, and city-based location information, derived from the IP address used to access the interface) that is tracked using vendors such as google analytics, will use UUID (the random number generated on first log-in specific to that participant) to track these data. The mobile app and web-application data used to manage the patient condition is held on the secure server in the OUHNHS intranet, only accessible via a secure log-in portal (i.e. the web-application) hosted in a different OUH-NHS server to that which is open to the internet. All data is communicated using encrypted channels: data communication from/to the web-application and mobile app will use HTTPS (SSL/TLS encryption protocols); the security of SMSs sent to the patients is managed by the SMS service company (i.e. Esendex), and the UK-based mobile operator provider of each patient.

Only those researchers within CCRF directly involved in the study will be provided with a log-in, as well as the CI/PI, the obstetricians involved in clinical care and the technical support team from OIBME. As participants will only be identified by initials, study ID and contact number it will not affect the blinding of the research team as all of these participants are in the intervention arm and it will not be realistically possible to link back to a particular participant without accessing the high compliance database where such information will be stored. This would only be done in the event of unblinding being required. If a researcher is accidentally un-blinded, they will not partake in subsequent downstream data analysis.

Users with administration roles in the web-application are able to export a pseudo-anonymised version of the trial data (i.e. initials and phone number are removed) through the web-application. All the data stored in the OUH-NHS intranet server are backed-up daily in the same server. Only data from the last 3 days are kept. The backups are further encrypted using an AES encryption protocol.

14. Quality assurance procedures

Risk assessment

The trial will be conducted in accordance with the current approved protocol, GCP, relevant regulations and standard operating procedures. A risk assessment and monitoring plan are not being prepared before the study opens, as it is a low risk intervention.

Monitoring

Direct access will be granted to authorised representatives from the Sponsor within the appropriate department and host institution for monitoring and/or audit of the study to ensure compliance with regulations. Following written standard operating procedures, the monitoring visits will verify that the clinical trial is conducted and data are generated, documented and reported in compliance with the protocol, GCP and the applicable regulatory requirements.

Trial Steering Committee

A trial steering committee (TSC) will convene prior to the study starting and half-yearly thereafter to review and address key aspects of the study including the following:

- Recruitment
- Safety/adverse event
- Withdrawals
- Data management
- Statistical analysis plan

The TSC will also function as a data safety and monitoring committee (DSMC) for this particular study and there will be a smaller trial management committee as outlined in the study synopsis, which will focus more on the week-to-week running of the trial and will be on a more regular basis.

15. Protocol deviations

A trial related deviation is a departure from the ethically approved trial protocol or other trial document or process (e.g. consent process) or from Good Clinical Practice (GCP) or any applicable regulatory requirements. Any deviations from the protocol will be documented in a protocol deviation form and filed in the trial master file.

A standard operating procedure is in place within CTRG in the University of Oxford for identifying noncompliances, escalation to the central team and assessment of whether a non-compliance /deviation may be a potential Serious Breach.

16. Serious breaches

The Medicines for Human Use (Clinical Trials) Regulations contain a requirement for the notification of "serious breaches" to the MHRA within 7 days of the Sponsor becoming aware of the breach. A serious breach is defined as “A breach of GCP or the trial protocol which is likely to affect to a significant degree:

- The safety or physical or mental integrity of the subjects of the trial; or
- The scientific value of the trial”.

In the event that a serious breach is suspected, the Sponsor must be contacted within 1 working day. In collaboration with the CI, the serious breach will be reviewed by the Sponsor and, if appropriate, the Sponsor will report it to the REC committee, Regulatory authority and the relevant NHS host organisation within seven calendar days.

17. Ethical and regulatory considerations

Declaration of Helsinki

The Investigator will ensure that this trial is conducted in accordance with the principles of the Declaration of Helsinki.

Guidelines for Good Clinical Practice

The Investigator will ensure that this trial is conducted in accordance with relevant regulations and with Good Clinical Practice.

Approvals

Following Sponsor approval, the protocol, informed consent form, participant information sheet, participant invitation letters and study advertising materials will be submitted to an appropriate Research Ethics Committee (REC), HRA, and MHRA for written approval. The Investigator will submit and, where necessary, obtain approval from the above parties for all substantial amendments to the original approved documents.

Other Ethical Considerations

Study visits

See Appendix F for the additional considerations required during the COVID-19 pandemic.

The clinical team will contact the research team via ‘Whatsapp’ ® messaging in which no ‘patientidentifiers such as name, date of birth or address’ will be mentioned, although this form of communication does have secure end-to-end encryption e.g. clinical team will send message stating ‘eligible patient on MAU in bed 5. Will provide details when you arrive on ward.’

All of the initial procedures in the baseline visit will be done at the bedside to minimise the burden of study participation and to allow mum and baby/babies to remain together and feed as and when is needed. The provision of home visits for week 1 and 6 is also to minimise the burden on the participant.

The final study visit, can take up to a maximum of 4 hours at a busy time in the participants' lives. This has been considered in several PPI meetings. The consensus of agreement was to minimise the burden on the mum and child/children. It was thus agreed that the team would contact the participant several weeks prior to the final visit and:

- Participants will be advised to try and make arrangements for the child not to attend the visit e.g. determine whether plans can be made to stay with the father or other family members.
- Where this is not possible, help with child-care can be offered by research midwives with the relevant DBS approval and safe-guarding training, and places for breast-feeding, a nappy changing area, and a box of child's entertainment will be provided. A double-bedded bay will be booked for this visit so there is enough space for feeding and playing.
- We can also offer split MRI visits in some cases (as long as the MRI does not extend beyond 9 months from enrolment).
- We have also made a distinction between essential procedures (which must take place) and some of the more exploratory secondary desirable procedures (which could be skipped if it necessary to shorten the visit) as per the diagram in section 9.

24 hour Ambulatory Blood Pressure and week-long activity Monitoring

Wearing an ambulatory blood pressure monitor has no risks and causes no pain. It is used routinely in hospital outpatients to assess blood pressure variation over a 24-hour period. The regular inflation and deflation of the cuff at regular time points may be initially unsettling to the participant however, it is unlikely that it will disturb their sleep, particularly as the cuffs provided as comfier than standard NHS monitors, the monitors can be programmed to be silent, and the frequency of readings is reduced (compared to the NHS) to minimise the burden. These details are explained in the PIS and participants will be shown how to use the device and provided with the relevant device information sheet. A trained member of the study team will also show participants how to re-attach it as it is routinely removed for a bath or shower and, they will be asked to remove it for any period of intense cardiovascular activity e.g. competitive sport, where the readings would be not representative of their normal ambulatory blood pressure. They can also remove it for short periods during the 24hr period where it may be an inconvenience. The weeklong activity monitor (accelerometer) is no different to wearing a wrist-watch and carries no risk and causes no pain.

Unexpected health related findings

It is possible that the study investigations could uncover unexpected disease findings. For any abnormalities confirmed, the CCRF departmental SOP (approved by University of Oxford) will be followed. A designated clinical specialist will discuss the implications with the participant and, with the consent of the participant, may arrange for further investigations as necessary. Participants will be aware from the PIS that research studies are not for diagnostic purposes, and therefore are not a substitute for a clinical appointment. Investigators will gain permission from the participant to contact their general practitioner (GP) directly so that the GP can then arrange appropriate management, or will refer the participant to a specialist hypertension clinic.

Sensitive issues

When addressing the exclusion criteria e.g., 'a significant new co-morbidity that makes enrolment unsafe or impractical' the study team are aware that this may be a sensitive issue and cause distress. The questions will thus be phrased as compassionately as possible and team members will be briefed in advance on how to broach this aspect of the study visit.

Language barriers

Those without acceptable English language skills will be excluded as it would be hard to safely ensure they are able to comply with self-management as all the instructions, reminders and contact with the research team will be done in English.

Home visits

In line with the University's lone working policy [https://www.admin.ox.ac.uk/safety/policy-statements/s508/#d.en.21010] whenever visiting participants in their home, researchers will inform a staff member at research site where they are going to be. The researcher will telephone that person when they arrive

at the home. If the researcher has any concerns about the situation, then he/she will leave immediately. Ideally, no research will be undertaken in a participant's home unless two people can conduct the visit together, although it is appreciated that this is not always possible. If there is thought to be a significant risk associated with a particular visit, a specific risk assessment (expanding on the points above) will be made and recorded.

Appropriate safeguards are in place for 'safe-guarding' which follow the University of Oxford's safeguarding code of practice. This was devised in response to the Safeguarding act of 2015, 'Working Together to Safeguard Children 2015'. The full policy is detailed at:
http://www.admin.ox.ac.uk/personnel/cops/safeguarding/safeguardingcop/

The University Council approved this policy on 22 June 2015 to take effect from 1 October 2015. In line with this, all staff working on the study Extension are familiar with the Universities' child protection policy and procedures for dealing with issues of concern or abuse (full policy outlined in the link above). The study investigators working on the study have also undertaken the Oxfordshire Safeguarding Children Board's 'introduction to safeguarding' e-learning training, in line with the University's recommendations as part of their policy. They all have appropriate certification of satisfactory completion of this training and the PI will delegate any safe guarding matters to a nominated study team member. The nominated safeguarding contact for the study is responsible for ensuring that concerns and allegations are escalated to the appropriate University Safeguarding Officer immediately as outlined further in this hyperlink (http://www.admin.ox.ac.uk/personnel/cops/safeguarding/safecontact/).

Phlebotomy (and Cannulation for those in the gadolinium sub-study)

Risk to Participants: Common risks associated with phlebotomy and cannulation are pain during the procedure and bruising (with associated pain afterwards). The worry associated with taking blood may cause some participants to feel unwell or faint before, during or after the procedure. The risk associated with this will be reduced by having an adequately equipped facility for performing the procedure and having a staff member trained in basic life support. Although phlebotomy and cannulation is a very safe procedure, it does create a puncture wound on the skin, which may very rarely lead to infection around the puncture site. The risk of this will be minimised by ensuring strict hygiene during the procedure. In the event that a participant reports symptoms of an infection (local redness, swelling, pain or discharge of pus) they should be referred to their GP or to A+E urgently.

Risk to Researchers/Other Staff: Taking blood cares a risk of needle stick injury to the phlebotomist, which in turn carries a risk of exposure to blood borne infections. This risk will be minimised by a) ensuring staff are adequately trained, b) ensuring staff have been vaccinated against, and show immunity to Hepatitis B and c) having a local policy for needle stick injury which describes the process of being assessed for and receiving post exposure prophylaxis.

Echocardiogram cardiac ultrasound scans

Although echocardiography carries no risk of physical harm to the participant, there is a risk to personal discomfort or embarrassment to the participant. This is minimised by providing gowns or scrubs to maintain modesty and dignity at all times. A female sonographer will be provided wherever possible.

MRI scans

MRI is a safe and non-invasive technique with no known risk when appropriately supervised. It does not involve ionising radiation (X-rays). Potential participants with ferromagnetic objects in their bodies or with implanted devices which can be damaged by the magnet will be excluded by carefully screening all subjects for ferromagnetic objects, metal implants and other metal (e.g. shrapnel injury) every time prior to entering the scanner environment. The University of Oxford Centre for Magnetic Resonance Research (OCMR) is fully equipped for resuscitation (including defibrillation) in the unlikely event of a medical emergency during scanning and doctors performing and/or supervising the scans are trained in Advanced Life Support. While most people do not experience discomfort in a MRI environment, the enclosed space of the scanner can potentially feel uncomfortable. Discomfort from lying still for a long period will be minimised with comfortable padding and positioning. People with a history of claustrophobia would be excluded from participation in the study. Participants will be given a chance to see the scanner before the study starts. Whilst in the scanner, participants are able to use the alarm button or can squeeze a bulb placed in their hands if they

wish to communicate with the operator or to interrupt the scanning at any stage of the scanning process. As the MRI scanner is noisy, participants would be fitted with ear-plugs and/or acoustically shielded headphones to minimize the noise and aid communication between participants and investigators. Participants will be provided with OCMR scrubs/gowns for the CMR scans that preserve their modesty while remaining loose in the scanner to avoid potential burns from synthetic clothing. To help maintain participant dignity they will be asked to leave their underwear on, so long as it has no metal parts (e.g. zips, bra clasps or studs). Participants will be asked to change in a changing room near to the scanner into OCMR approved clothing and will be given a locker to securely store their belongings. If they are unable to change into the MRI safe clothing themselves a member of staff will be on hand to offer assistance.

In the unlikely event of seeing any structural abnormalities on a scan, a clinical specialist will check the scan and with the inclusion of the kidneys and the brain in the MRI scan, a nominated consultant radiologist has agreed to review any images where there is concern; and issue a formal report to the patient's GP. If the specialist feels that the abnormality was medically important, they will discuss the implications with the participant and arrange for further investigations as necessary. Participants will not be informed unless the doctor considers the finding has clear implications for their current or future health. It is important to note that scans are not carried out for diagnostic purposes, and therefore the scans are not a substitute for a clinical appointment. Rather, the scans are intended for research purposes only.

Gadolinium sub-study

Currently, gadolinium-based contrast agents are widely used in MRI scans to answer important medical questions, and for advancing scientific discovery and improving clinical care. Gadolinium contrast is widely used for clinical indications in CMR and is safe to use. Occasionally (< 1 in 1000), it may cause a mild headache, rash and very rarely a more severe allergic reaction. These severe reactions generally respond very well to standard emergency drug treatment, similar to that given for other severe allergic reactions. However, in people with reduced kidney function, it can lead to a rare condition called nephrogenic systemic fibrosis (NSF); hence, as per departmental SOPs based on Royal College of Radiologists guidelines, (2007) only research participants with estimated glomerular filtration rate (eGFR) >30ml/minutes can be given gadolinium. For this study, all potential participants with eGFR <30ml/minutes will not be recruited. Participants' consent will be sought to obtain a pre–scan blood test to check kidney function if there is no laboratory blood result for creatinine within the last 6 months prior to consent, or if investigators make a clinical judgement that a new creatinine result is needed. As explained to the participant in the PIL, no gadolinium will be given before this result is available. It is known that small amounts of gadolinium deposits can remain in the body, including the skin, bones and the brain. There has been no scientific evidence to-date that these gadolinium deposits are harmful or lead to adverse health effects, although this is an active area of research. If new information relevant to a participant's health becomes available regarding the use of these contrast agents, a study investigator will discuss this with the participant to allow them to decide if they wish to continue in the study.

Cardiopulmonary Exercise Testing

Cardiopulmonary exercise testing is very safe and is even used in people with heart failure. However, as with all forms of hard exercise, very occasionally some people have significant changes in their heart rate and rhythm that requires medical attention. Although the risk of this happening is small, the test is carried out in a room equipped with emergency monitoring, emergency medications and resuscitation equipment. Medical personnel will be available throughout the testing. Participants' heart rate and electrocardiography (ECG) are monitored throughout the exercise testing and the exercise test will be stopped if there are any concerns. The study procedures including the cardiopulmonary exercise testing are explained in the Participant Information sheet and will be made clear to the participants as part of the informed consent process. If there are any concerns regarding a participant's safety during the exercise testing they will be asked to stop and safety to continue in the trial will be reviewed by trained and qualified personnel. Doctors who are Advanced Life Support trained will be available during CPET testing and the site is fully equipped for resuscitation (including defibrillation) in the unlikely event of a medical emergency during the test.

Reporting

The CI shall submit once a year throughout the clinical trial, or on request, an Annual Progress Report to the REC, HRA (where required), host organisation, funder (where required) and Sponsor. In addition, an End of Trial notification and final report will be submitted to the MHRA, the REC, host organisation and Sponsor.

Participant confidentiality

The study will comply with the General Data Protection Regulation (GDPR) and Data Protection Act 2018, which require data to be de-identified as soon as it is practical to do so. The processing of the personal data of participants will be minimised by making use of a unique participant study number only on all study documents and any electronic database(s), with the exception of the CRF, where participant initials may be added. All documents will be stored securely and only accessible by study staff and authorised personnel. The study staff will safeguard the privacy of participants' personal data.

Expenses and benefits

Reasonable travel expenses for any visits will be reimbursed on production of receipts, or a mileage allowance provided as appropriate. The participants will also be provided with a £30 gift voucher as a 'thank-you' for the time given up, after the final study visit has been completed and the 24hr blood pressure monitor and 7 day activity monitor have been returned to CCRF. The app notifications and usage are free when in WIFI zone but if using 3G/4G they may be charged depending on their network-provider. If this is the case, then any cost incurred will be reimbursed to the participant. Participants in the sub- study will receive a £30 gift voucher as a 'thank you' for the time given up.

18. Finance and insurance

Funding

The research is being financed by a British Heart Foundation Clinical Research Training Fellowship (BHF Grant number FS/19/7/34148).

Insurance

The University has a specialist insurance policy in place, which would operate in the event of any participant suffering harm because of their involvement in the research (Newline Underwriting Management Ltd, at Lloyd's of London). NHS indemnity operates in respect of the clinical treatment that is provided.

Contractual arrangements

Appropriate contractual arrangements will be put in place with all third parties and collaborators

19. Publication policy

The Investigators will be involved in reviewing drafts of the manuscripts, abstracts, press releases and any other publications arising from the study. Authors will acknowledge that the study was funded by the British Heart Foundation Clinical Research Training Fellowship (BHF Grant number FS/19/7/34148). Authorship will be determined in accordance with the ICMJE guidelines and other contributors will be acknowledged. The summarised results will be published in a scientific journal/s and summarised on the CCRF website for participants to read. Should participants wish to have a copy of any papers published, they merely need to contact the study team, using the contact details provided on their PIS, and the team would be happy to provide one.

20. Development of a new product/ process or the generation of intellectual property

Ownership of IP generated by employees of the University vests in the University. The protection and exploitation of any new IP is managed by the University's technology transfer office, Oxford University Innovations.

21. Archiving

Personal data (such as contact details and information, which could identify a participant) will be destroyed as soon as it is practical to do so and no later than 12 months after the end of the study. The personal identifiers (name of participant) contained in consent forms and the code break document will be stored or accessed for up to 10 years after study end, after which time the custodian will agree a date for destruction and it will be destroyed confidentially. As part of our commitment to maximise patient/service user involvement in research, participants will give consent (optional) for their contact details to be retained. This will be kept securely and independently of the study records. This will allow investigators to contact participants about future ethically approved research.

22. Appendices

Appendix A: Trial flow chart

Records Screening
Notes will be screened in relevant maternity care areas for participants with pre-eclampsia and gestational hypertension

Eligibility check

These stages will be performed by members of the clinical care team and if participant eligible research team will be contacted

Consent and Enrolment (n= 200)
100 to intervention 100 to control to allow for 20% withdrawal/loss

Research team will reconfirm eligibility and consent the participant. If recruited antenatally consent will also be re-confirmed post-natally

Randomisation after delivery of baby/babies

N= 100 to intervention arm and taught to use Blue-tooth BP monitor and smartphone app prior to discharge (by an un-blinded separate member of team)

N= 100 randomised to control arm who will be followed up post discharge as per usual care as well as the 4 study visits all participants receive

Study Visit 1 (Baseline) On the ward

- Demographics and Anthropometry & Questionnaires
- Manual blood pressure measurement
- Bed-side Trans-Thoracic Echo
- Collate data from medical notes and NHS blood results
- Blood test
- Intervention only: Automated BP cuff supplied & POP-HT app and physician assisted medication adjustment. Control arm may be provided BP cuff if NHS one not available in line with RCOG guidance during COVID-19

Desirable but not essential:

- Vicorder®

Study Visits 2 and 3 (at weeks 1 and 6 post-partum) Home/CCRF

- Demographics and Anthropometry
- Manual blood pressure measurement
- EQ-5D-5L quality of life questionnaire
- Fit 24hr BP monitor (*V3 only*) - *OPTIONAL*

AS PER APPENDIX E THE COVID-19 PANDEMIC NECESSITATES THE SWITCH TO REMOTE V2 AND V3, STEPS TO MINIMISE CONTACT DURING V1/BASELINE VISIT AND EXTENDING V4 FROM 6-9 for 24HR BP and 6-12 MONTHS for other secondary and tertiary outcome measures

Study Visit 4 (6+/-3 months) CCRF and OCMR

- Demographics and Anthropometry
- Manual blood pressure measurement
- Trans-thoracic Echo
- EQ-5D-5L quality of life questionnaire
- MRI heart and brain **and kidney**
- Blood test
- Fit 24hr BP monitor
- Fit week-long activity monitor

Desirable but not essential

- Vicorder®
- Completion of Lifestyle and dietary questionnaire
- Retinal imaging
- CPET and Exercise Echo
- Formal review of medical and obstetric history and medication(s)

Data analysis

Data analysis, Statistical interpretation and drafting of publications

Appendix B: Schedule of procedures (main study)

**Procedures**	**Visit 0: Consent**		**Visit 1: Baseline**		**Visit 2**		**Visit 3**		**Visit 4**	
	Intervention arm	Control arm	Intervention arm	Control arm	Intervention arm	Control arm	Intervention arm	Control arm	Intervention arm	Control arm
**Eligibility assessment**	X	X								
**Informed consent**	X	X								
**BP measurement**			X	X	X	X	X	X	X	X
**Demographics & anthropometry**			X	X	X	X	X	X	X	X
**Echocardiogram**			X	X						
**Data collection: medical notes and NHS blood results**			X	X						
**Lifestyle & Diet questionnaire**			X	X					X	X
**Vicorder ®(vascular assessment)**			X	X					X	X
**<u>Intervention:</u> Automated BP cuff provision and Smartphone app installation**			X	During pandemic cuff may be provided						
**Home BP selfmonitoring and physician assisted medication**					X		X		X	

**adjustment post hospital discharge**										
**EQ-5D-5L questionnaire**					X	X	X	X	X	X
**Fitting 24hr blood pressure monitor**							X	X	X	X
**Trans-thoracic echocardiogram**									X	X
**MRI of heart and brain <u>and kidney</u>**									X	X
**Blood test**			X	X					X	X
**Fit accelerometer**									X	X
**Retinal imaging**									X	X
**Review of medical and obstetric history**									X	X
**CPET with exercise echo at 40% workload**									X	X

Appendix C: POP-HT Validation sub study

**Procedures**	**Visit 1**		**Visit 2**	
**Eligibility assessment**	X	X		
**Informed consent**	X	X		
**BP measurement**	X	X	X	X
**Demographics & anthropometry**	X	X	X	X
**Blood test**	X	X	X	X

Appendix D: Estimated duration of each study procedure (main study)

**Study visit 1: Baseline**	**Measure**	**Time (maximum 75-100 minutes with participant)**
	Consent (if not already obtained previously)<br>Review and confirmation of consent if already obtained previously	30<br>5
	Demographics & Anthropometry	10
	Bed-side blood pressure measurement	10
	Bedside Vicorder® (vascular measure) (desirable)	10
	Bedside trans-thoracic echo	15
	Lifestyle and diet questionnaire (desirable and can be completed at a later date)	25
	Collate data from medical notes and NHS blood results (participant not required for this part)	20
	For those randomised to the intervention arm: Automated BP cuff provision and Smartphone app installation by a separate un-blinded team member (once the team performing baseline visit have left the ward)	30
	Blood Sampling/Venepuncture	10
**Study visits 2 and 3 at week 1 and 6 postpartum respectively**	**Measure**	**Time (~30 minutes per visit)**
	Demographics and Anthropometry including reconfirmation of consent	10
	Blood pressure measurement	10
	EQ-5D-5L quality of life questionnaire	5
	Fit 24 hour BP (visit 3 only)	5
**Study visit 4: 6 +/- 3 months**	**Measure**	**Time (maximum 4 hours)**
	Demographics and Anthropometry	10
	Manual blood pressure measurement	10
	Vicorder® (vascular measures) (desirable)	10
	Trans-thoracic Echo	15
	Retinal imaging (desirable)	10
	CPET with Exercise Echo (desirable)	30
	MRI (with blood sampling post scan if consented to late gadolinium and ECV techniques) and MRI safety questionnaire	60
	Fit 24hr BP and activity monitor	10
	Lifestyle and diet questionnaire (desirable)	25
	EQ-5D-5L questionnaire	5
	Review of medical and obstetric history and any medication side effects (desirable)	15

	Blood sampling/Venepuncture	10

Appendix E: Amendment history

Amendment No.	Protocol Version No.	Date issued	Author(s) of changes	Details of Changes made
01 (minor)	N/A	21/01/2020	J Kitt	Correction to wording of consent form clauses<br>Correction of version listed in flyer footer<br>IRAS form updated to list PI for OUH site
02 (minor)	V2.0	19/03/2020	P Kemp	Add option for Visit 2 (Weeks 1) and Visit 3 (Week 6) to be conducted remotely by video call.<br><br>Add the option of sending a sterile OMRON EVOLV BP monitor to control arm participants prior to Visit 3 (Week 6).<br><br>Make the "fitting of a 24-hr home blood pressure monitor" procedure optional for Visit 3 and adding the option of conducting this at a later time point.
03 (minor)	V3.0	22/04/2020	J Kitt and P Kemp	Further changes to visits due to the COVID-19 pandemic:<br>1. Add provision of BP monitor to control arm;<br>2. Consent process to be modified to reduce risk of transmissions of COVID-19;<br>3. Extend the time point for Visit 4 to 6-12 months from 6-9 for outcome measures except the primary outcome and BP based secondary outcomes;<br>4. Change the randomisation and blinding process as only one member of staff (wearing PPE where necessary) can be present at Visit 1;<br>5. Minimise participant contact during the baseline visit via use of tablets/iPads for reviewing the PIS/flyer;<br>6. Vicorder is now an optional measurement during the baseline visit.
04 (substantial)	V4.0	06/10/2020	A Frost and P Kemp	Add optional blood test to baseline visit in both control and intervention hypertensive group for 20 participants and include a sub population of 20 normotensive postnatal women for a newly added blood-validation sub study.
05 (minor)	V5.0	05/01/2021	J Kitt and P Kemp	Add option for Visit 4/Final visit to be performed remotely by video call for the primary study outcome and BP based secondary outcome measures

06 (substantial)	V6.0	23/03/2021	J Kitt	1. Addition of an extra 2 minute sequence during the MRI scan to also evaluate the kidneys (T1 maps)<br>2. Changing the study from a single, blinded RCT to a PROBE design as a result of COVID-19<br>3. Removal of laser speckle procedure (because it was never carried out on any participants due to COVID-19) as investigator unable to travel to UK<br>4. Rephrasing of primary objective in line with SAP and published protocol paper with reordering and rephrasing of secondary and tertiary objectives in line with SAP and protocol paper<br>5. Rephrasing of the wording for those patients have the blood test as part of the endothelial cell sub-study in amendment 4 to make it less ambiguous

Appendix F: Rationale and amendment proposal for restarting POP-HT during COVID-19

The following explanation summarises our reasons to allow the POP-HT RCT to restart during the COVID-19 pandemic, as an exception to Stage 3 of the OUH NHS Foundation Trust guidance. We currently have an exception to Stage 3 for follow up of women already recruited based on the importance of provision of clinical care to these women. Based on our experience during the follow up we believe ongoing recruitment into this trial will allow women in both the intervention and control arm to receive 'optimal' clinical care during the current disruption of normal care. This will ensure consistency with adherence to the updated Royal College of Obstetrics and Gynaecology (RCOG) guidance[32] on the care of women during the COVID-19 pandemic within the OUH NHS Trust. Trials of self-monitoring and self-management are safe and effective in other clinical scenarios [33].

<u>Background</u>

Hypertensive pregnancy and risk of complications post-partum - Women who have a pregnancy complicated by preeclampsia/gestational hypertension have a high risk of complications and fluctuations in blood pressure control during the post-partum period that require monitoring. Specific guidance to manage this risk is described by the Royal College of Obstetrics and Gynaecology (RCOG). However, following such guidance is problematic during a period when access to normal midwife and GP services is reduced, triggering the amendment to their guidance described in point 3 below.

Experience from POP-HT Trial - Based on the first 18 patients recruited to this randomised trial, prior to the COVID-19 pandemic halting recruitment, 10 were randomised to the control arm and 8 to the intervention arm. During the study follow up, 3 of the 10 controls (30%) were identified with elevated readings requiring a change in management, with one being identified with clinical signs (raised JVP and oedema) that required emergency management. In the intervention arm only 1 of 8 (12.5%) required a brief readmission for blood tests and could then be managed with medication up-titration via tele-monitoring and a further 2 of 8 (25%) were managed safely via the proprietary POP-HT app avoiding re-admission.

RCOG guidance during COVID-19: In recognition of the problems with routine clinical review the RCOG have amended their guidance[32] to recommend home BP monitoring (self-monitoring) 2 to 3 times per week for women discharged after a hypertensive pregnancy. Take up of this guidance has been sporadic because of the change in practice and requirement for additional equipment (blood pressure monitors) this advice necessitates.

Rationale for restart of POP-HT

Provision of guideline clinical care in control and intervention arm during pandemic - POP-HT provides access to ongoing post-partum care for women after an episode of preeclampsia. This is either consistent with current RCOG guidance on usual care in the absence of 'normal' midwife and GP care with provision of blood pressure monitors (control arm) or an enhanced level of clinical care with additional remote, physician optimised selfmanagement (intervention arm).

Reduced need for hospital attendance during pandemic through participation in trial – Current early data, and previous work within a pilot study, indicates the intervention arm have a lower incidence of hospital attendance reducing risk for contracting COVID-19 for both mother and child in this arm. This requires validation in the full study but would be a secondary benefit for some participants within the trial, if consistent. In addition, review assessments that form part of the research study protocol for both control and intervention arms allows clinical assessments to identify onset of problems or reassure safety to remain at home. These assessments replicate some of the 'normal' GP and midwife care that is currently difficult to deliver.

Safety of proposal to re-start

Remote study visits already established - To manage follow up at the start of the pandemic the study was amended to allow entirely remote follow up visits for week 1 and week 6, equivalent to visits 2 and 3 (nonsubstantial amendment 2.0 25/03/2020). Over the past 6 weeks trialling of remote follow up has been undertaken and shown to be effective and feasible. No participants have required face-face contact while review of medication, use of the POP-HT app and remote blood pressure measurement (both clinic and ambulatory 24hr blood pressure monitoring) has been achieved for all participants.

Proposed amendment of baseline visit – No new baseline visits have yet been performed during the COVID-19 pandemic but we have re-designed the recruitment, consent and enrolment process to minimises direct patient contact and risk of virus transmission:

- Provision of documents: PIS, flyer and additional information sheet will all be provided by the clinical team to the participant on a Tablet/ipad® (sterilised with CLINELL® wipes). The participants can then review them in this format (and a copy will be e-mailed to them for their records once consent has been obtained and they are enrolled).
- Consent: Consent forms will be placed in wipe down wallets, which will be handed to the participant for signing wearing sterile gloves. Once signed the form will be photocopied whilst wearing gloves. The copy will then be placed back into a sterile wallet for the participant and the original will be placed in a second wallet. Both will be wiped down with CLINELL® wipes and the research teams's copy will be kept securely in a wipe-down file/ring-binder in 'quarantine' before moving them to CCRF after 48hours.
- The Vicorder® is now an optional measurement
- The Echo and Vicorder® will now be performed by a single investigator (Dr Jamie Kitt) at the bedside. PPE will be used where necessary in line with hospital policy. Adequate training in donning and doffing of PPE has been undertaken by Dr Kitt via OXSTAR who delivered training to OUH NHS Staff (Train the Trainer Course). This will not affect OUH trust protocols for female chaperones, which can still be provided if needed by existing clinical staff on the ward.

Summary

The reduced level of routine care during the COVID-19 pandemic introduces significant variation into the clinical care of women who have had preeclampsia or gestational hypertension who are at risk of complications after discharge from secondary care. We believe that restarting recruitment into POP-HT will provide, at the minimum the recommended standard of clinical care advocated by the updated RCOG guidance[32] within the control arm, as well as the planned trial of more enhanced care within the intervention arm. The trial has already been adapted, with tests of feasibility, to allow remote follow up and careful consideration has been given to the effective and safe restart of baseline visits.

23. References

1. Ananth, C.V., K.M. Keyes, and R.J. Wapner, *Pre-eclampsia rates in the United States, 1980-2010: age-period-cohort analysis.* BMJ, 2013. **347**: p. f6564.
2. Podymow, T. and P. August, *Postpartum course of gestational hypertension and preeclampsia.* Hypertens Pregnancy, 2010. **29**(3): p. 294-300.
3. Walters, B.N. and T. Walters, *Hypertension in the puerperium.* Lancet, 1987. **2**(8554): p. 330.
4. Cairns, A.E., et al., *Self-Management of Postnatal Hypertension: The SNAP-HT Trial.* Hypertension (Dallas, Tex, 2018. **: 1979). 72**(2): p. 425-432.
5. Lazdam, M., et al., PP032. Unique features of long-term cardiovascular phenotype in young women with early-onset pre-eclampsia. Pregnancy Hypertens, 2012. **2**(3): p. 259-60.
6. Behrens, I., et al., Risk of post-pregnancy hypertension in women with a history of hypertensive disorders of pregnancy: nationwide cohort study. BMJ, 2017. **358**: p. j3078.
7. McDonald, S.D., et al., Cardiovascular sequelae of preeclampsia/eclampsia: a systematic review and meta-analyses. Am Heart J, 2008. **156**(5): p. 918-30.
8. Ray, J.G., et al., Cardiovascular health after maternal placental syndromes (CHAMPS): populationbased retrospective cohort study. Lancet, 2005. **366**(9499): p. 1797-803.
9. Bushnell, C., et al., Guidelines for the prevention of stroke in women: a statement for healthcare professionals from the American Heart Association/American Stroke Association. Stroke, 2014. **45**(5): p. 154588.
10. European Society of Cardiology, et al., ESC Guidelines on the management of cardiovascular diseases during pregnancy: the Task Force on the Management of Cardiovascular Diseases during Pregnancy of the European Society of Cardiology (ESC). Eur Heart J, 2011. **32**(24): p. 3147-97.
11. Law, M.R., J.K. Morris, and N.J. Wald, Use of blood pressure lowering drugs in the prevention of cardiovascular disease: meta-analysis of 147 randomised trials in the context of expectations from prospective epidemiological studies. BMJ, 2009. **338**: p. b1665.
12. NICE CG110., *Hypertension in pregnancy: the management of hypertensive disorders during pregnancy.* 2010: Published by the Royal College of Obstetricians and Gynaecologists.
13. National Institute for Health and Care Excellence (NICE), CLINICAL GUIDELINE CG 107: Hypertension in pregnancy: diagnosis and management. NICE GUIDELINES, PUBLISHED 2010, UPDATED 2011 and draft update publish February 2019. 2011, NICE: NICE.
14. National Institute for Health and Care Excellence (NICE), *Hypertension in Pregnancy: diagnosis and management*, NICE, Editor. 2019: https://www.nice.org.uk/guidance/ng133.
15. Webb, A.J., et al., Response of day-to-day home blood pressure variability by antihypertensive drug class after transient ischemic attack or nondisabling stroke. Stroke, 2014. **45**(10): p. 2967-73.
16. Webb, A.J.S., et al., Prognostic Significance of Blood Pressure Variability on Beat-to-Beat Monitoring After Transient Ischemic Attack and Stroke. Stroke, 2018. **49**(1): p. 62-67.
17. Siepmann, T., et al., Long-term cerebral white and gray matter changes after preeclampsia. Neurology, 2017. **88**(13): p. 1256-1264.
18. Boardman, H., et al., Comprehensive multi-modality assessment of regional and global arterial structure and function in adults born preterm. Hypertens Res, 2016. **39**(1): p. 39-45.
19. Melchiorre, K., et al., Maternal cardiac dysfunction and remodeling in women with preeclampsia at term. Hypertension, 2011. **57**(1): p. 85-93.
20. Melchiorre, K. and B. Thilaganathan, *Maternal cardiac function in preeclampsia.* Curr Opin Obstet Gynecol, 2011. **23**(6): p. 440-7.
21. Ghossein-Doha, C., et al., Age-related alterations in cardiac geometry in formerly preeclamptic women and healthy parous controls: an explorative study. Reprod Sci, 2013. **20**(1): p. 39-44.
22. Ghossein-Doha, C., et al., Hypertension after preeclampsia is preceded by changes in cardiac structure and function. Hypertension, 2013. **62**(2): p. 382-90.
23. Ghossein-Doha, C., et al., Maternal cardiac adaptation to subsequent pregnancy in formerly preeclamptic women according to recurrence of pre-eclampsia. Ultrasound Obstet Gynecol, 2016. **47**(1): p. 96-103. 24. Lazdam, M., et al., Prevention of vascular dysfunction after preeclampsia: a potential long-term outcome measure and an emerging goal for treatment. J Pregnancy, 2012. **2012**: p. 704146.
25. Lazdam, M., et al., Elevated blood pressure in offspring born premature to hypertensive pregnancy: is endothelial dysfunction the underlying vascular mechanism? Hypertension, 2010. **56**(1): p. 159-65.

26. Gyselaers, W. and B. Thilaganathan, *Preeclampsia: a gestational cardiorenal syndrome.* J Physiol, 2019. **597**(18): p. 4695-4714.
27. McManus, R.J., et al., Effect of self-monitoring and medication self-titration on systolic blood pressure in hypertensive patients at high risk of cardiovascular disease: the TASMIN-SR randomized clinical trial.
JAMA, 2014. **312**(8): p. 799-808.
28. McManus, R.J., et al., Efficacy of self-monitored blood pressure, with or without telemonitoring, for titration of antihypertensive medication (TASMINH4): an unmasked randomised controlled trial. Lancet, 2018. **391**(10124): p. 949-959.
29. McManus, R.J., et al., Telemonitoring and self-management in the control of hypertension (TASMINH2): a randomised controlled trial. Lancet, 2010. **376**(9736): p. 163-72.
30. Topouchian, J., et al., Clinical accuracy of the Omron M3 Comfort((R)) and the Omron Evolv((R)) for self-blood pressure measurements in pregnancy and pre-eclampsia - validation according to the Universal Standard Protocol. Vasc Health Risk Manag, 2018. **14**: p. 189-197.
31. Melchiorre, K., et al., Preeclampsia is associated with persistent postpartum cardiovascular impairment. Hypertension, 2011. **58**(4): p. 709-15.

Statistical analysis plan: Physician Optimised Post-partum Hypertension Treatment (POP-HT) trial

Date and Version No: Version 1.0 22/6/2021

		**Name**	**Title**	**Signature**	**Date**
Written by		Dr Jamie Kitt	BHF Clinical Research Training Fellow	Jamie Kitt	23.06.2021
Reviewed by		Dr Jill Mollison	Senior Trial Statistician		28.06.2021
Approved by		Prof Paul Leeson	PI of POP-HT		23.06.2021
Approved by		Prof Richard McManus	Co-investigator and Supervisor		
Approved by		Dr Adam Lewandowski	Co-investigator and Supervisor		06/07/2021

Version History

Version:	Version Date:	Changes:
1.0	22/06/2021	Original

1. Abbreviations

AE	Adverse event
ABPM	Ambulatory Blood pressure monitoring
CCRF	Cardiovascular Clinical Research Facility
CPET	Cardiopulmonary exercise test
CRF	Case Report Form
CTRG	Clinical Trials and Research Governance
DMC/DMSC	Data Monitoring Committee / Data Monitoring and Safety Committee
ECV	Extra-cellular volume
ESC	European Society of Cardiology
GCP	Good Clinical Practice
GLS	Global Longitudinal Strain
GP	General Practitioner
HRA	Health Research Authority
ICF	Informed Consent Form
IUGR	Intra-uterine growth restriction
LA	Left atrium
LV	Left ventricle
MHRA	Medicines and Healthcare products Regulatory Agency
NICE	National Institute for Health and Care Excellence (NICE)
OCMR	Oxford Centre for Cardiovascular Magnetic Resonance Imaging
OIBME	Oxford Institute for Biomedical Engineering
PROBE	Prospectively Randomised Open Blinded End-point study
NHS	National Health Service
NIHR	National Institute for Health Research
RES	Research Ethics Service
PI	Principal Investigator
PIS	Participant/ Patient Information Sheet

PPE	Personal Protective Equipment
PW	Pulse wave
REC	Research Ethics Committee
SAE	Serious Adverse Event
SOP	Standard Operating Procedure
VAS	Visual Analogue Scale

2. Introduction

Preface

Chief Investigator: Prof Paul Leeson
Supervisors: Prof Richard McManus, Prof Paul Leeson, Dr Adam Lewandowski
Supervising Trial Statistician (s): Dr Jill Mollison and Dr Milensu Shanyide
Study title: Physician Optimised Post-partum Hypertension Treatment trial
Short title: POP-HT TRIAL
Ethics reference: 19/LO/1901

Purpose and scope of the plan

This document details the proposed presentation and analysis for the main paper (s) reporting results of the POP-HT trial. The results reported in these papers should follow the strategy set out here. Subsequent analyses of a more explanatory nature will not be bound by this strategy, although they are expected to follow the broad principles laid down here. The principles are not intended to curtail exploratory analyses (for e.g. to decide cut off points for categorisation of continuous variables), nor to prohibit accepted practices e.g. data transformation prior to analysis, but they are intended to establish the rules that will be followed, as closely as possible, when analysing and reporting the trial.

The analysis strategy will be available on request when the principal papers are submitted for publication in a journal. Suggestions for the subsequent analyses by journal editors or referees, will be considered carefully, and carried out as far as possible in line with the principles of this analysis strategy, if reported, the source of the suggestion will be acknowledged.

This version of the Statistical Analysis Plan was written based on protocol version 6.0 23/03/2021.

Objectives

The trial is a single centre, Prospective Randomised Open Blinded End-point (PROBE) study. Women who develop hypertensive disorders of pregnancy, which require on-going treatment for blood pressure after birth at the time of discharge, will be randomised to one of two treatment arms: usual care, or self-management of blood pressure. Women will be recruited from the John Radcliffe Hospital and this study will investigate the effectiveness of post-partum physician assisted self-management of blood pressure vs standard care over the first 6 (up to 9) months post-partum, although the time point for analysis of the secondary outcome measures was extended up-to 12 months post-partum as a result of the COVID19 pandemic as part of a protocol amendment (see table A1).

	Objectives	Outcome Measures	Timepoint(s)
Primary	To compare diastolic BP in the intervention arm to the control arm post-partum.	24 hour average diastolic BP measured by SPACELAB 90217 24hr Ambulatory blood pressure monitoring (ABPM)	Primary timepoint at 6 months

Secondary	To compare the effect of the intervention on cardiovascular, cerebrovascular and vascular phenotypes	BP based<br>a) 24 hr average systolic blood pressure assessed by SPACELAB 90217 24hr ABPM<br>b) Mean diurnal diastolic blood pressure assessed by SPACELAB 90217 ABPM<br>c) Mean diurnal systolic blood pressure assessed by SPACELAB 90217 ABPM<br>d) Mean nocturnal diastolic blood pressure assessed by SPACELAB 90217 24hr ABPM<br>e) Mean nocturnal systolic blood pressure assessed by SPACELAB 90217 24hr ABPM<br>f) Mean bedside diastolic blood pressure measured during study visit (mean of 2+3)<br>g) Mean bedside systolic blood pressure measured during study visit (mean of 2+3)<br><br>Cardiac MR<br>h) Left ventricular (LV) mass indexed to end-diastolic volume and body surface area (BSA)<br>i) LV EDV indexed to BSA<br>j) LV wall thickness (MRI) – septum, posterior and RWT<br>k) LA volume indexed to BSA<br>l) Right ventricular (RV) mass indexed to end-diastolic volume and body surface area (MRI)<br>m) RV EDV indexed to BSA<br>n) RA volume indexed to BSA<br>o) LV ejection fraction (EF) & RV EF<br>p) LV and RV stroke volumes indexed to BSA<br>q) Myocardial fibrosis (for those in gadolinium sub-study)<br>r) ECV (for those in gadolinium substudy))<br><br>Echo<br>s) LV Diastolic function: E/E' average, E/A ratio, E deceleration time.<br>t) Global longitudinal strain (GLS)<br>u) LV wall thickness (Septal, posterior and RWT)<br>v) LV systolic function (EF by Biplane Simpsons)<br>w) LA volume by Biplanar assessment<br><br>Vascular:<br>x) Pulse wave velocity	Weeks 6 and 6months for the 24 hr ABPM<br><br>Baseline, week 1, 6 and 6months for the bedside blood pressures<br><br>For Cardiac MR at 6 months<br><br>At baseline and at 6 months postpartum for Echo outcome measures<br><br>PWV, Aortic BP and AI at baseline and at

		y) Augmentation index<br>z) Aortic BP aa) Aortic distensibility (MRI)<br><br><u>Cerebrovascular MR</u><br>bb) Total white matter hyperintensity volume<br>cc) Cerebral blood flow dd) Mean vessel thickness of the middle and posterior cerebral arteries, and internal carotid artery<br><br><u>Retinal</u><br>ee) the corrected central retinal arteriolar equivalent<br>ff) the corrected central retinal venular equivalent<br>gg) the corrected central retinal arteriolar equivalent/corrected central retinal venular equivalent ratio.<br><br><u>Exercise Echo</u><br>Exercise ejection fraction (echo) at 50% of predicted peak workload during a bicycle cardio-pulmonary exercise test (CPET) (prediction based on age and resting heart rate)<br>Exercise LA volume at 50% predicted peak workload<br><br><u>CPET</u><br>VO2 at VT1	612 months (aortic stiffness)<br>Aortic compliance (on MRI) at 6-12 months<br><br>6 months postpartum for these MRI measures.<br><br>6 months postpartum for all retinal measures<br><br>6 months postpartum<br><br>6 months postpartum
Tertiary:	In vitro vascular function in the sub-study of 20 women*<br><br>To explore presence/absence of kidney injury and fibroinflammatory status<br><br>Quality of life assessment<br><br>Participant experience: assessment of individual experience following intervention<br>Number of readmissions	Assessment of biomarker levels associated with endothelial dysfunction in 20 normotensive and 20 hypertensive women to determine if BP improvement can affect vascular function*<br><br>T1 mapping of the kidneys to look at cortico-medullary differentiation<br><br>EQ-5D-5L health questionnaire : VAS score + index value<br><br>Qualitative semi-structured interviews as well as assessment of acceptability and feasibility within the intervention arm	Baseline and 6month postpartum<br><br>6 months postpartum<br><br>Baseline, week 1, week 6 and 6 months postpartum<br><br>6 months postpartum

<table>
<tr><td></td><td>in intervention vs control arm

Side-effect impact</td><td>Readmission number in each arm

Number and frequency of side-effects reported (intervention via the app and control during follow up calls/SMS)</td><td>12 months postpartum

12 months postpartum</td></tr>
<tr><td>Intervention(s)</td><td colspan="3">The intervention will consist of physician-optimised self-management of post-partum BP. Women will follow a ‘smartphone’ app based algorithm for medication-titration, which will provide individualised dose titration advice. This is overseen and any change is approved by physicians who can review the uploaded readings and respond to telemonitored abnormal readings in a timely fashion.</td></tr>
<tr><td>Comparator</td><td colspan="3">The control arm will be managed as per usual NHS led care with assessment by their own health care professionals and adjustment of their medications as is needed. The BP of this group will be monitored and recorded at the same time-points and in the same manner as the intervention arm as will all other secondary outcome measures.</td></tr>
</table>

Blood validation sub-study

We aim to recruit 20 normotensive participants as a reference population for endothelial function to compare with the endothelial function of 20 of the 200 hypertensive participants recruited into the mainstudy. The purpose of the sub study is to provide a reference population of women not affected by hypertensive disease. In the sub study, healthy postnatal women will undergo measurements of specific characteristics of blood cells and circulating factors involved in inflammation and endothelial dysfunction. This population will validate how blood cells and circulating factors vary naturally and may be affected by external factors such as mode of delivery.

3. Trial design

The trial is a single centre, Prospective Randomised Open Blinded End-point (PROBE) study. Women who develop hypertensive disorders of pregnancy, which require on-going treatment for blood pressure after birth at the time of discharge, will be randomised to one of two treatment arms: usual care, or self-management of blood pressure. Women will be recruited from the John Radcliffe Hospital and this study will investigate the effectiveness of post-partum, physician assisted self-management of blood pressure vs standard NHS care in the post-partum period.

Main trial setting and participant profile

We aim to recruit 200 participants of which we will randomise 100 to the intervention arm and 100 to the control arm. The intervention arm will comprise tele-monitored home blood pressure monitoring (including periods of home 24hr ABPM) coupled with physician-assisted self-management. The control arm will receive ‘standard’ levels of NHS care from their GP and midwives and health visitors. All participants will be recruited from the Oxford Women’s Centre at the John Radcliffe Hospital, which sees approximately 25 patients per month with hypertensive pregnancies.

Main study visit and measures

The expected duration of participant involvement will be 6 (up to 12) months from enrolment to study completion and participants will be asked to attend four study visits after their pre-screening and enrolment: baseline, at 1 week, at 6 weeks and at 6 months. At times when face to face appointments are restricted due to regulations related to the COVID-19 pandemic, the week 1 and week 6 visits can be done remotely where needed to avoid putting participants at undue risk. The primary outcome will be studied over the narrower time frame of 6-9 months as originally described at the study outset, even during the COVID-19 pandemic, but a further amendment (see table A1 of supplementary material) allows all BP based primary and secondary outcomes to be done remotely, if needed. The other secondary outcomes can be done over a wider timeframe of 6-12 months to allow participants to safely attend their study visit at the hospital.

4. Definition and derivation of outcome measures

Primary outcome

The primary outcome for this study is the 24-hour overall average diastolic blood pressure (mmHg) at the final visit (V4), assessed by SPACELAB 90217® 24hr Ambulatory blood pressure monitor adjusted for clinic (i.e. bedside) baseline diastolic BP at the time on enrolment.

Derivation: When performing a 24hr BP monitor, cuff size is checked before fitting the monitor on the mid-left arm and an appropriate cuff size is fitted, in a standardised manner. BP Data is automatically recorded at 30 minute intervals between 7am and 10pm and at 60 minute intervals from 10pm to 7am.
Diurnal readings are defined as those between 0700-2200 and nocturnal from 2200-0700 for consistency. The monitor is fitted during the V4 at 6 months post-partum.

All monitors are programmed to be 'silent' as well as to not show participants the value of the readings to minimise any white coat/alerting response. Participants complete a standardised diary sheet to record sleep and awake times and any periods of exercise that could confound readings. Data are uploaded from the monitor to SPACELABS proprietary SENTINEL ® software, which produces a standardised report of 24 hour overall diastolic and systolic readings with standard deviations (SD), daytime/awake diastolic and systolic readings with SDs and nocturnal diastolic and systolic readings with SDs. Any manual readings (which can be obtained by pushing the blue button on the 24 ABPM device) accidentally, or when the device is first fitted to confirm adequate placement, are excluded from the analysis and these are automatically flagged and removed by the software. Any readings corresponding as 'exercise' or as a 'daytime nap', as exemplified on the sample diary sheet included at the end of the SAP, during the period of recording are also excluded.

NICE guideline (NG136)[34] states that at least 2 measurements per hour are taken during the person's usual waking hours and the average value of at least 14 measurements are to be taken during the person's usual waking hours for a monitoring period to be valid. In cases where this is not achieved, women will be asked to repeat the monitor as soon as possible and within the stipulate time frames laid out in the protocol (i.e. for V4 the time period is 6-9 months post-discharge). In cases where repeat monitors are needed, a blinded investigator will then analyse the data from both sets of 24hr monitoring periods to obtain: 24 hour overall diastolic and systolic readings with SDs, daytime/awake diastolic and systolic readings with SDs deviations, and nocturnal diastolic and systolic readings with SDs. If unable to obtain a repeat set of readings a blinded adjudication committee will be decided to determine whether to include/exclude the first set of readings and if so, whether diurnal, nocturnal, all or none. In such cases included, the data will be starred and referenced to explain the number of participants with incomplete 24hr BP data.

24hr BP Data is exported from SENTINEL® to CASTOR®, an electronic CRF system, by an independent investigator, and from there it can be exported for analysis by the study statistician, who is masked.

Secondary outcomes

Statistical analysis of the secondary outcomes will be performed blind to treatment allocation. Imaging variables, because of their known operator variability will be subject to an inter-operator variability check in 10-20% of cases i.e. the analysis of 10-20% will be repeated by a 2nd independent, member of the team at

random, and data will be presented on inter-user variability e.g. For echo assessment of LV function, LA volume and MRI assessment of LV and RV volumes.

The time frames stipulated in the protocol for the 24hr BP based secondary outcomes are as follows: for V3 the data will be collected at 6 weeks (+/- 5 days post discharge) and for V4, data will be collected at 69 months post-partum.

<u>24hr BP monitor based secondary outcomes:</u>

- 24 hr average systolic blood pressure assessed by SPACELAB 90217 24hr Ambulatory blood pressure monitoring at the V3 and V4
- Mean diurnal diastolic blood pressure assessed by SPACELAB 90217 24hr Ambulatory blood pressure monitoring at the V3 and V4
- Mean diurnal systolic blood pressure assessed by SPACELAB 90217 24hr Ambulatory blood pressure monitoring at the V3 and V4
- Mean nocturnal diastolic blood pressure assessed by SPACELAB 90217 24hr Ambulatory blood pressure monitoring at the V3 and V4
- Mean nocturnal systolic blood pressure assessed by SPACELAB 90217 24hr Ambulatory blood pressure monitoring at the V3 and V4

N.B. Please refer to the Derivation section for the primary outcome, for what constitutes a diurnal and nocturnal readings on a 24hr BP monitor and for further details on their derivation.

<u>Bedside BP based secondary outcomes:</u>

For the bedside BP outcomes listed below, the three blood pressure readings will be obtained from the left arm as described in the protocol paper during each study visit i.e. a participants will have their blood pressure checked using the automated mode of a validated, calibrated sphygmomanometer. Three blood pressure readings will be taken at intervals of 1 minute. The first reading will be discarded and the 2nd and 3rd reading used to calculate the mean blood pressure. The measurement technique advised by the British Heart Foundation will be strictly followed i.e. BP will be measured after 5 minutes' rest with the participant sat/laying at 45 degrees. They will be asked not to talk and, the arm will be supported and cuff positioned at the height of the heart, with their legs uncrossed. V2 takes placed at 7 +/- 5 days, V3 at week 6 +/-5 days following discharge and V4 at 6-9 months post-partum.

- Mean bedside diastolic blood pressure, measured during the study visits (mean of readings 2+3) at V2, V3 and V4
- Mean bedside systolic blood pressure measured during study visit (mean of readings 2+3) at V2, V3 and V4.

If a participant does not complete the study visit e.g. they miss their week 1 follow up then no data will be included for that participant for that timepoint. Mean data for each time-point will only be reported where complete data is available e.g. All 220 participants recruited have blood pressure data from their baseline visit, but if 20 patients are lost to follow up/withdrawn by V4 then only 200/220 will be reported and this will be highlighted in the relevant table. All BP data is entered in real-time during each study visit into CASTOR edc®. Data-cleaning and export will be done by a blinded study investigator.

Additional analysis on the BP secondary outcome measures:
Potential confounding factors for the blood pressure values are collected during the period of enrolment from baseline to the time of V4 completion. These include questionnaire based self-reported salt intake (low/moderate or high), alcohol intake (number of units), smoking history, BMI and objectively recorded activity levels (measured by a validated wrist-worn accelerometer). These will be described per randomised group and differences between groups will be estimated using a mean difference and 95% confidence intervals.

<u>Other secondary outcome measures:</u>

For the remainder of the secondary outcomes, due to the impact of COVID, protocol amendments (see table A1 of supplementary material) were submitted, and approved, to extend the time-frame for the outcomes listed below to 6-12 months post-partum. Where possible all data is acquired in the same visit but if patients are unwilling to attend the hospital during national lockdowns or due to COVID concerns, the primary and BP based secondary outcomes will be done remotely or as a home visit; the secondary outcomes below are collected at a later date at the hospital within the 6-12 month window post-partum.

Cardiac MRI based:

- Left ventricular (LV) mass indexed to end-diastolic volume and body surface area (LVMI/EDV/BSA; g/ml/m2) during the V4,
- LV end-diastolic volume (EDV) indexed to body surface area (LV EDV/BSA; ml/m^2); during the V4
- LV wall thickness indexed to BSA – septum, posterior and RWT (mm/m^2); during the V4
- Left atrial (LA) volume indexed to BSA (ml/m^2); during the V4
- Right ventricular (RV) mass indexed to end-diastolic volume and body surface area (RVMI/EDV/BSA; g/ml/m2); during the V4
- RV end-diastolic volume (EDV) indexed to body surface area (RV EDV/BSA; ml/m^2); during the V4
- Right atrial (RA) volume indexed to BSA (ml/m^2); during the V4
- LV ejection fraction (EF) and RV EF (absolute percentage (%)); during the V4 • LV and RV stroke volumes indexed to BSA (ml/m^2); during the V4

All measurements will be performed using CIRCLE® CVI42 proprietary software and downstream analysis of the MRI images will be done by a fully blinded investigator. Height (cm) and weight (kg) is obtained using the same calibrated scales immediately prior to the MRI (when wearing same departmental scrubs and no shoes) in order to calculate body surface areas (BSA) by Mostellar equation.

An optional sub-study involving administration of Gadolinium contrast will be offered as an additional component to the MRI, in those women who are not breast feeding. This is part of exploratory work that may feed into a larger future trial and outcomes related to this sub-study include:

- Myocardial fibrosis – this will be described as the overall number of participants in each group with any degree of myocardial fibrosis and also the number with each pattern of fibrosis e.g. midwall band, patchy enhancement of the RV/LV junction, epicardial.
- ECV (extra-cellular volume) will be derived with regions of interest drawn pre- (A) and postcontrast (B) agent with the equation: myocardial ECV = (1 − Haematocrit) × ($\Delta R1_{myocardium}/\Delta R1_{blood}$), where R1 = 1/T1. The post-contrast map will be obtained 15 min post-Gadovist® contrast bolus. The Haematocrit is obtained immediately prior to the MRI scan during cannulation.

Echo measures:
All echo images are obtained as part of a standard BSE echo dataset. The V1 (baseline visit) is done on the postnatal ward prior to discharge as described in the protocol paper.
The following measures of systolic and diastolic function will be assessed:

- E/E’ average; at the baseline visit (V1) and at the final visit (V4)
- E/A ratio; at the baseline visit (V1) and at the final visit (V4)
- E deceleration time; at the baseline visit (V1) and at the final visit (V4)
- LV systolic function (ejection fraction by Biplane Simpson’s method); at the baseline visit (V1) and at the final visit (V4)

Echo will also be used to assess:

- Left atrial (LA) biplanar volume; at the baseline visit (V1) and at the final visit (V4)
- Septal wall, posterior wall and relative wall thickness indexed to BSA (mm/m^2); at the baseline visit (V1) and at the final visit (V4)

- Global longitudinal strain (GLS); at the baseline visit (V1) and at the final visit (V4)

All echo-based image analysis will be performed using Philipps ISCV® and TOMTEC® proprietary software and analysed in line with British Society of Echo (BSE) guidelines. All image analysis will be done on fully de-identified images so that no allocation is known at the time of analysis.

Vascular measures:

- Pulse wave velocity (m/s); at the baseline visit (V1) and at the final visit (V4)
- Augmentation index (%); at the baseline visit (V1) and at the final visit (V4)
- Aortic BP (mmHg); at the baseline visit (V1) and at the final visit (V4)
- Aortic distensibility (MRI) at the single time-point of 6-12 months post-partum

During the final visit (V4) aortic BP, PWV, AI measures are obtained whilst the patient remains in the MRI scanner in the same time, and position, that the aortic cine images are obtained by the cardiac MRI scanner.

Cerebrovascular measures:

- Total white matter hyper-intensity volume indexed to BSA (um/m^2) during the final study visit (V4)
- Cerebral blood flow (ml/s) during the final study visit (V4)
- Mean vessel thickness of the middle and posterior cerebral arteries and internal carotid artery (mm) during the final study visit (V4)

Retinal measures:

- The corrected central retinal arteriolar equivalent during the final study visit (V4)
- The corrected central retinal venular equivalent during the final study visit (V4)
- Corrected central retinal arteriolar equivalent/corrected central retinal venular equivalent ratio during the final study visit (V4)

Exercise echo measures:

- Exercise ejection fraction (echo) at 50% of predicted peak workload (based on their age and resting heart rate). This will be monitored during a bicycle cardio-pulmonary exercise test (CPET) and the baseline EF (%), exercise EF (%) and percentage changed will be presented as a mean and standard deviation. This measure is done at a single time-point during the final study visit (V4).
- Exercise LA volume will be measured at 50% of their predicted peak workload based on their age and resting heart rate. This will be monitored during a bicycle cardio-pulmonary exercise test (CPET) and the baseline LA volume (ml/m^2), exercise LA volume (ml/m^2) and the percentage changed will be presented as a mean and standard deviation . This measure is done at a single time-point during the final study visit (V4).

Cardiopulmonary exercise test measures:

- The VO2 at VT1 (litres/minute) be measured (during a continuously monitored bicycle CPET starting at 20W and increasing by 15W/minute in a RAMP protocol). The VO2 (l/min) will be presented as a mean and standard deviation in each group.

Tertiary outcomes

- EQ-5D VAS score and index value will be presented as a mean and standard deviation in each allocation (intervention and control), for each study visit, at each timepoint from baseline to the final V4.
- Readmission number in each arm; will be reported as an absolute number per each allocation.
- Number of side-effects reported (intervention reporting via the app; and control during follow up calls/SMS/medical records), will be reported as number of side effects per person and/ or the n (%) of participants with at least 1 side effect.

There are a number of exploratory tertiary outcomes including:

- T1 mapping of the kidneys to look at cortico-medullary differentiation of a sub-set of individuals as part of a protocol amendment. T1 values will be reported as a mean and standard deviation for each group.
- The blood validation sub-study. The outcome measure for this sub-study will be biomarker levels associated with endothelial dysfunction, which will be reported at baseline and at 6-12 months as a mean and standard deviation for each arm (hypertensive vs normotensive).

Target population Inclusion criteria

- Participant is willing and able to give informed consent for participation in the trial.
- Female, aged 18 years or above.
- Clinician confirmed diagnosis of either gestational hypertension or pre-eclampsia defined by NICE NG 133[34]
- Requiring anti-hypertensive medication at the point of discharge from secondary care.
- Participant has clinically acceptable laboratory results and clinical course post-partum with no other adverse complicating factor requiring prolonged admission post-partum that would make participation unfeasible as judged by the CI. Examples would include stroke sequalae, ongoing DIC, the baby/babies requiring prolonged NICU/SCBU admission of >1 week.
- In the Investigator's opinion, is able and willing to comply with all trial requirements including ownership of a 'Smart-phone/Tablet' and willing to use the smart-phone app if randomised to that arm.
- Sufficient competence in English Language to follow the app instructions and partake in the study, as judged by the CI

Exclusion criteria

The participant may not enter the study if ANY of the following apply:

- Significant renal or hepatic impairment that would affect safe medication titration and adjustment as part of the trial, as deemed by the Investigator.
- Participant with life expectancy of less than 6 months.
- Any other significant disease or disorder, which, in the opinion of the Investigator, may either, put the participants at risk because of participation in the trial, or may influence the result of the trial, or the participant's ability to participate in the trial.
- Participants who have participated in another research trial involving an investigational product in the past 12 weeks.
- Women with pre-existing hypertension (essential hypertension) will be excluded, as this is a separate pathology that would affect the efficacy of the study intervention and affect the primary and secondary outcomes of the study.

An absolute contra-indication to MRI (as per MRI safety questionnaire) precludes them partaking in the MRI but not in the remainder of the study

Additional exclusion criteria specific to the Gadolinium sub-study are:

- Breast feeding, • eGFR <30ml/minute.

Sample size

Power calculations to determine adequate sample sizes for this trial are summarised below:

Primary outcome measure: 24-hour average diastolic blood pressure (mmHg) at 6-9 months post-partum as assessed by SPACELAB 90217 24hr Ambulatory blood pressure monitor

Sample size calculation: The detection of BP differences between the 2 arms of this trial is based on the mean diastolic blood pressure difference detected in the pilot SNAP-HT study at 6 months. The mean BP difference detected between the intervention and control arm at the 6 month time-point was -4.5mmHg. We have used a more conservative standard deviation (SD) of 10mmHg in each arm (in SNAP-HT the SD was 8.2mmHg in the intervention arm and 9.8 mmHg in the standard care arm[4]) and 10mmHg SD is in keeping with pooled SDs for ambulatory diastolic blood pressure readings from other studies. To detect a treatment effect on diastolic blood pressure of -4.5mmHg, powered to 80% at p=0.05 requires a total sample size of 158 and with 1:1 randomisation this would require 79 in each arm. We adjusted our power calculations to determine the final sample size, to allow for up to 20% loss to follow up/withdrawal based on prior experience. Thus, we aim to recruit 100 to the intervention and 100 to the control arm.

During COVID-19 a Royal College of Obstetricians and Gynaecology (RCOG) guideline[32] was issued that recommended a home BP monitor be given to all women for the first week after discharge. As a result of the potential dilution of the effect size that self-monitoring in the control group could have, we recalculated our sample size. A systematic review [35] concluded that self-monitoring in the control could lead to a potential 0.42mmHg dilution of the impact of self-management on diastolic BP at 6 months, measured using 24hr ABPM. Therefore, assuming the same SDs of 10mmHg in each arm as in our original submission to the BHF, we subtracted 0.42mmHg from the 4.5mmHg between group difference we had originally powered on. To remain powered at > 80% and we would require 95 in each group (190 total) and hence we planned to over-recruit to 220 (rather than the original 200) to allow for a larger withdrawal/loss to follow up rate in light of this.

Secondary outcome hypothesis: Improved blood pressure control in the post-partum period (0-9 months) in POP-HT will result in improved cardiac, vascular and cerebrovascular phenotypes during the V4.

Secondary outcome power calculations:

- Cardiac MRI: Studies using echocardiography by our collaborators have compared BP and LV mass in pre-eclampsia patients and control patients, at 1 year post-partum [19, 31][19, 31][19, 31][19, 31][19, 31][19, 31]. They found that a difference in BP at 1 year of 10mmHg in diastolic BP corresponded to significant differences in LV mass. SNAP-HT appeared to achieve a 50% reduction of anticipated BP difference seen between pre-eclamptic and normotensives at 1 year by 6 months i.e. ~5mmHg. If it is assumed that the structural/phenotypic benefit results from the BP benefit, as we are hypothesising, then we must power to detect 50% of the phenotypic difference. In previous work by our group we have demonstrated significant differences in LV mass/EDV g/ml) in a similar age and predominantly female population with similar mean diastolic BP differences between groups to that seen in SNAP-HT. The LV mass/EDV (g/ml) in the group with high normal blood pressure was 1.54g/ml vs. 1.22 g/ml in those with optimal blood pressure with a standard deviation of 0.33 and 0.27 respectively at P<0.001. Based on these assumptions, to observe a treatment effect of 0.16 (50% of the difference between 1.54g/ml and 1.22g/ml) on LV mass/EDV, requires 67 in the intervention arm and 67 in the control arm (132 total). This is calculated using the larger SD of 0.33 referenced above at a power of >80% to detect a difference between the groups at p=0.05. This number should take into account for the greater dropout rate we may see for the MRI outcomes.

- Cerebrovascular MRI *:* Work by our group [17], on pre-eclamptic pregnancy, showed an increased burden of temporal lobe white matter lesion volume 5-10 years after a pre-eclamptic pregnancy (23.2±13 μl) vs matched individuals who had a normotensive pregnancy (10.9 ± 11.5 μl) at p<0.05.If we again assume we can detect a 50% of the phenotypic benefit with our intervention as outlined above, we would anticipate a 50% reduction in the burden of white matter lesions i.e. 6.15 μl (50% of 23.2 – 10.9 μl) in the intervention arm. With 71 in the intervention group and 71 in the control group (142 total), this will provide >80% power at p=0.05, even using the more conservative SD of 13ul to detect a 50% improvement in white matter lesion volume between the intervention and the control group. This number should take into account for the greater dropout rate we may see for the MRI outcomes.

- Aortic compliance: Several studies assessing the impact of blood pressure on aortic compliance have shown that even modest reductions in systolic/diastolic blood pressure increase aortic distensibility/compliance[36]. One such study had a mean difference in systolic blood pressure of 4.6mmHg between the 2 drug treatment arms at 52 weeks, akin to the same mean difference in SNAP-HT at 6 months, albeit this was diastolic not systolic, although other studies have suggested diastolic BP may be even more important in influencing aortic compliance. In this study with a mean 4.6mmHg difference in systolic BP there was a treatment difference of 0.12 [36][(95% CI −0.35, 0.60), *P* = 0.60 in aortic compliance. Based on these assumptions, to observe a treatment effect from our intervention, with 100 in the intervention and 100 in the control arm we will be more than powered at >90% to detect a difference at P=0.05 in POP-HT.

- Exercise ejection fraction: Huckstep et al [37]from our group compared resting and exercise ejection fractions for young adults with high normal BP vs. a normotensive cohort. The cohort was very well matched demographically to our planned study cohort, albeit it included both males and females. Resting ejection fraction (by Biplane Simpson's) was similar between groups but at 40%60% of peak exercise intensity, the higher blood pressure group had a lower exercise ejection fraction than the normotensive cohort (73.9±3.25 vs. 80.0±4.54%, p<0.001) and in keeping with this, a smaller increase in ejection fraction when going from baseline to 40% exercise intensity (10.4±5.92 vs. 19.0±6.90%, p<0.001). Assuming the ~5mmHg mean BP improvement achieved in SNAP-HT again translates to a 50% phenotypic benefit, when assessing exercise ejection fraction we anticipate a 4.3% improvement in exercise stress ejection fraction in the intervention arm vs. the control arm (4.3% is 50% of the difference i.e. 50% of 10.4-19%). With 43 participants in the intervention arm and 43 in the control arm (86 total) we will be powered at >80% to detect such a difference at p=0.05, which will take account of the lower number likely to undertake the CPET at the final study visit.

5. Analysis – General considerations

Descriptive statistics

Continuous variables will be reported as means with standard deviations (or medians with Interquartile ranges if skewed). Categorical variables will be reported as counts and percentages.

Measurements will include 24-hour Ambulatory blood pressure measurements (mean diurnal, mean nocturnal and overall 24-hour average readings for both diastolic and systolic blood pressure) as well as the 'clinic/bed-side' BP measurements made during the study visits will be estimated using a mean difference and 95% confidence interval with a P-value.

Characteristics of participants

At baseline each group will be described with regards the following characteristics:

- Age
- Parity
- Ethnicity
- Socioeconomic status (index of multiple deprivation)
- BMI
- Smoking history
- Family history of gestational hypertension or pre-eclampsia
- Prior history of gestational hypertension or pre-eclampsia
- Diagnosis – gestational hypertension or pre-eclampsia
- Gestation at diagnosis of gestational hypertension or pre-eclampsia (weeks +days)
- Duration of antenatal antihypertensive treatment
- Gestation at delivery
- Percentage with IUGR defined as less than (<) 40th centile abdominal circumference on the fetal growth scan
- Amount of anti-hypertensive treatment at time of randomisation (WHO defined daily dose)

- Clinic systolic and diastolic BP at booking/antenatal visit (mmHg)
- Clinic systolic and diastolic BP at the baseline visit (mean of readings 2 + 3; mmHg)

Frequencies and percentages will be reported for categorical variables and for continuous variables, the mean and standard deviation will be reported if normally distributed, and the median and interquartile range if not. No formal statistical testing will be applied to test for any difference between randomised groups with respect to the above baseline characteristics.

For salt intake, this will be classified as (low/moderate/high), based on self-reported answers to the British Heart Foundation validated diet questionnaire [38] contained within the POP-HT Lifestyle and diet questionnaire. This data is collected after randomisation.

Levels of exercise will be reported based on self-reported questionnaire answers (number of minutes per week) and by objective accelerometer measurement of activity (sedentary/mildly active/moderately active/highly active) [39]. This data is also collected after randomisation.

Definition of population for analysis

The analysis will be carried out on the basis of intention-to-treat (ITT). This is, participants will be analysed according to their allocated randomisation (intervention vs control arm) irrespective of the treatment they actually receive. Participants with at least one post randomisation outcome will be included in the analysis population.

Analysis will be conducted on all patients randomised that fulfilled the inclusion and exclusion criteria apart the following two scenarios:

- The formal diagnosis of any 'secondary' cause of hypertension such as pheochromocytoma, Conn's syndrome, bilateral renal artery stenosis, aortic coarctation or other condition known to influence BP. This list is not exhaustive and any other co-morbidities that may be contributory to blood pressure elevation/reduction will be adjudicated by the PI/CI who will remain blinded to randomisation
- The participant withdraws from the trial and withdraws consent retrospectively for data already collected

Data Monitoring Committee and Interim Analyses

A trial steering committee (TSC) will convene prior to the study starting and half-yearly thereafter to review and address key aspects of the study including the following:

- Recruitment
- Safety/adverse event
- Withdrawals
- Data management
- Statistical analysis plan

The TSC will also function as a data safety and monitoring committee (DSMC) for this study.

6. Primary analysis

Primary outcome

A linear mixed model will be applied to compare the groups with respect to the primary outcome. The model will include baseline bedside (i.e. clinic) diastolic BP (mean of the 2nd and 3rd bedside diastolic blood pressures, randomised group and minimisation factors (gestational age at the time of presentation with pre-eclampsia/gestational hypertension (continuous) and prescription of ACE inhibitor at randomisation) as fixed effects. Participant will be included as a random effect.

For all participants included in the primary outcome analysis, the mean 24-hour average diastolic blood pressure will be reported by randomised group. Adjusted mean differences between randomised groups with

95% confidence interval and p value will be estimated from the model for the following comparison: self-management (intervention) versus usual care (control) at 6 months.

Missing data

The rate of missing data is highly relevant and an important factor affecting the final data analysis. The frequency (with percentage) of losses to follow-up (defaulters and withdrawals) over the study will be reported by randomised group and compared between the groups.

Any deaths and their causes will be reported separately.

The availability of the outcome data for the primary and secondary outcomes (blood pressure measurements only) will be summarised by the two randomised groups. The mixed effects model implicitly accounts for data missing at random, however the data missing mechanism will be explored. A logistic regression model will explore any association between baseline characteristics and availability of the primary outcome. Any changes to the assumptions made in the primary analysis i.e. data missing at random, will be considered in a sensitivity analysis as described below.

Where 24hr BP data is not available or valid (as described in section 4.1), the participant will have missing data for the primary outcome at that time point. A further sensitivity analysis will include all participants with missing primary outcome data imputed using multiple imputation.

Handling outliers

A possible outlier is defined as a data-point greater than 3 standard deviations from the mean of its distribution in the variable at the time-point. For outliers that have not already been queried in data cleaning they will be queried for double-checking at this stage. Valid BP data is defined by NICE NG133[34] as explain in detail within section 4.

7. Secondary analysis

Secondary objectives

Secondary Outcomes have been grouped by variable type.

Continuous variables

Those secondary outcomes that represent a continuous variable will be reported as a mean and standard deviation in each group. The mean difference between groups at 6 months and 95% CI will be reported alongside a P value from the linear mixed model.

<u>Blood pressure based secondary outcomes:</u>

A linear mixed model will be applied to compare the groups with respect to BP outcomes. The model will be adjusted for baseline BP (bedside) and minimisation factors. Participant will be included as a random effect. The adjusted mean difference in BP between the groups, 95% CI for adjusted mean difference in BP and P value will be reported at each post randomisation time point. The unadjusted mean (SD) BP at each time point will also be reported.

Factors known to influence baseline blood pressure prior to enrolment e.g. age, parity, BMI at booking will be described as a number and percentage in each group in the baseline characteristics table.

<u>Non-blood pressure based secondary outcomes:</u>

This includes the cardiovascular and cerebrovascular MRI, echo, vascular, and retinal measures listed in section 4. Adjusted mean differences between randomised groups, 95% confidence interval and p values will be calculated for the following comparison: self-management (intervention) versus usual care (control). Given these secondary outcomes are almost all continuous variables, linear regression will be applied with

adjustment for minimisation factors. For any measures that do not satisfy the assumptions of linear regression a non-parametric test/regression will be carried out.

Handling missing data

The rate of missing data is highly relevant and an important factor affecting the final analysis. The rate of missing values will thus be summarised for each data point relevant the secondary outcomes as described in section 4 above.

Handling outliers

A possible outlier is defined as a data-point greater than 3 standard deviations from the mean of its distribution in the variable at the time-point. For outliers that have not already been queried in data cleaning they will be queried for double-checking at this stage.

8. Sensitivity analysis

We will perform sensitivity analysis on the primary outcome.

- Antenatal booking blood pressure recorded from the participants' week 12 booking visit to their midwives will be included as a covariate in the model. The reason we are performing a 2nd adjusted model is to assess the impact that antenatal values have on the intervention tested in this trial. Some groups (Hunt et el[40]) argue that pre-eclampsia is merely a progression/acceleration of higher antenatal blood pressure values, BMI and adiposity and we wish to explore this in POPHT with this 2nd model of adjustment. Data in the pilot SNAP-HT trial did not differ significantly when adjusted for antenatal booking blood pressure.
- If any baseline factors are associated with missingness then these variable(s) will be included in the mixed effect model, since the assumptions of the mixed model are missing at random which means that variables related to missingness should be included as covariates in the model.
- Participants with missing primary outcome measurements at 6 months will have 6-month outcome imputed using multiple imputation (MI), if necessary.

9. Tertiary/additional exploratory analysis

These include:

- EQ-VAS scores and the measure of utility score from the EQ-5D-5L questionnaires at baseline, week 1 and week 6 and 6 months. These scores will be presented as a mean and standard deviation in each group for each study visit. P values and a 95% confidence interval will be reported for the difference between the groups
- Readmission number in each arm (re-admissions included will be those related to blood pressure/complications of the hypertensive pregnancy over the duration of the entire study participation). These will be presented as an absolute number and percentage for each group of the study. No P value will be reported.
- Side-effects reported. The side-effects will be those captured via the app for those in the intervention or during follow up video calls/face-face visits/SMS (self-reported) or in the medical notes (electronic patient records). As the side effects are being recorded in different ways in the two arms of the trial, there could be some bias and more reporting of side effects in the intervention arm. We will therefore reporting the two sources of data separately i.e. the app and other sources. These will be presented as an absolute number and percentage for each group of the study. No P value will be reported.
- Biomarker levels associated with endothelial dysfunction, which will be reported at baseline and at 6-12 months as a mean and standard deviation for each arm (hypertensive vs normotensive) with a P value and a 95% confidence interval will be reported for the difference between the groups.

- Additional exploratory data is being collected on number of participants in each group with a positive COVID swab from time of enrolment to the V4 and on COVID vaccination statues (number and type of vaccine at the time of the V4).

10. Subgroup analysis and safety analysis

Subgroup analyses

These additional analyses will be performed on the primary outcome. An interaction term for randomised group x subgroup of interest will each be fitted in a linear mixed model, defined for the analysis of the primary outcome (section 6.1). We will report the P value for the interaction term for the subgroup of interest, subgroup specific treatment effects and 95% CI for each subgroup analysis.

- 1st vs 2nd/additional hypertensive pregnancy
- Index of multiple deprivation (IMD) score (split by median score for Oxfordshire vs those above and below the median within this study and/or using national quartiles)
- Ethnicity – White Caucasian vs. Non-White
- PET vs PIH

Safety analysis

Adverse events

Serious adverse events are not expected but any SAEs that occur during participation in the study will be reported as per GCP guidelines and the policy of the University of Oxford. Further detail, definitions of the relevant SAEs, and the policy for reporting them are contained within the protocol.

There are a number of expected admissions/consultations with healthcare providers that will be expected take place as part of the natural history of pre-eclampsia and gestational hypertension during the trial period. These will be classed as 'Foreseeable Events' exempt from reporting as SAEs and the list is contained with the protocol.

The number of the number of patients experiencing at least one adverse event as well as the number of events per group will be reported. All AEs are all reviewed periodically during the TSC and DSMC.

11. Validation

The primary outcome and the safety analysis will be independently analysed by a statistician from PCCTU and the secondary outcomes will be validated by the PC-CTU statistician who will re-run the code.

12. Changes to the protocol or previous versions of sap

This is the first version of the SAP but a number of amendments have been made to the protocol since the study began, as a result of the COVID-19 pandemic. These will not affect the SAP per se:

Amendment No.	Protocol Version No.	Date issued	Author(s) of changes	Details of Changes made

13. References

1. Ananth, C.V., K.M. Keyes, and R.J. Wapner, *Pre-eclampsia rates in the United States, 1980-2010: ageperiod-cohort analysis.* BMJ, 2013. **347**: p. f6564.
2. Podymow, T. and P. August, *Postpartum course of gestational hypertension and preeclampsia.* Hypertens Pregnancy, 2010. **29**(3): p. 294-300.
3. Walters, B.N. and T. Walters, *Hypertension in the puerperium.* Lancet, 1987. **2**(8554): p. 330.
4. Cairns, A.E., et al., *Self-Management of Postnatal Hypertension: The SNAP-HT Trial.* Hypertension (Dallas, Tex, 2018. **: 1979). 72**(2): p. 425-432.
5. Lazdam, M., et al., *PP032. Unique features of long-term cardiovascular phenotype in young women with early-onset pre-eclampsia.* Pregnancy Hypertens, 2012. **2**(3): p. 259-60.
6. Behrens, I., et al., *Risk of post-pregnancy hypertension in women with a history of hypertensive disorders of pregnancy: nationwide cohort study.* BMJ, 2017. **358**: p. j3078.
7. McDonald, S.D., et al., *Cardiovascular sequelae of preeclampsia/eclampsia: a systematic review and meta-analyses.* Am Heart J, 2008. **156**(5): p. 918-30.
8. Ray, J.G., et al., *Cardiovascular health after maternal placental syndromes (CHAMPS): populationbased retrospective cohort study.* Lancet, 2005. **366**(9499): p. 1797-803.
9. Bushnell, C., et al., *Guidelines for the prevention of stroke in women: a statement for healthcare professionals from the American Heart Association/American Stroke Association.* Stroke, 2014. **45**(5): p. 1545-88.
10. European Society of, G., et al., *ESC Guidelines on the management of cardiovascular diseases during pregnancy: the Task Force on the Management of Cardiovascular Diseases during Pregnancy of the European Society of Cardiology (ESC).* Eur Heart J, 2011. **32**(24): p. 3147-97.
11. Law, M.R., J.K. Morris, and N.J. Wald, *Use of blood pressure lowering drugs in the prevention of cardiovascular disease: meta-analysis of 147 randomised trials in the context of expectations from prospective epidemiological studies.* BMJ, 2009. **338**: p. b1665.
12. Health, N.C.C.f.W.s.a.C.s., *Hypertension in pregnancy: the management of hypertensive disorders during pregnancy.* 2010: Published by the Royal College of Obstetricians and Gynaecologists.
13. N.I.C.E, *N.I.F.C.E., CLINICAL GUIDELINE CG 107: Hypertension in pregnancy: diagnosis and management. NICE GUIDELINES, PUBLISHED 2010, UPDATED 2011 and draft update publish February 2019*. 2011, NICE: NICE.
14. Excellence, N.I.f.C., *Hypertension in Prengnacy: diagnosis and management*, NICE, Editor. 2019: https://www.nice.org.uk/guidance/ng133.
15. Webb, A.J., et al., *Response of day-to-day home blood pressure variability by antihypertensive drug class after transient ischemic attack or nondisabling stroke.* Stroke, 2014. **45**(10): p. 2967-73.
16. Webb, A.J.S., et al., *Prognostic Significance of Blood Pressure Variability on Beat-to-Beat Monitoring After Transient Ischemic Attack and Stroke.* Stroke, 2018. **49**(1): p. 62-67.
17. Siepmann, T., et al., *Long-term cerebral white and gray matter changes after preeclampsia.* Neurology, 2017. **88**(13): p. 1256-1264.
18. Boardman, H., et al., *Comprehensive multi-modality assessment of regional and global arterial structure and function in adults born preterm.* Hypertens Res, 2016. **39**(1): p. 39-45.
19. Melchiorre, K., et al., *Maternal cardiac dysfunction and remodeling in women with preeclampsia at term.* Hypertension, 2011. **57**(1): p. 85-93.
20. Melchiorre, K. and B. Thilaganathan, *Maternal cardiac function in preeclampsia.* Curr Opin Obstet Gynecol, 2011. **23**(6): p. 440-7.
21. Ghossein-Doha, C., et al., *Age-related alterations in cardiac geometry in formerly preeclamptic women and healthy parous controls: an explorative study.* Reprod Sci, 2013. **20**(1): p. 39-44.
22. Ghossein-Doha, C., et al., *Hypertension after preeclampsia is preceded by changes in cardiac structure and function.* Hypertension, 2013. **62**(2): p. 382-90.
23. Ghossein-Doha, C., et al., *Maternal cardiac adaptation to subsequent pregnancy in formerly preeclamptic women according to recurrence of pre-eclampsia.* Ultrasound Obstet Gynecol, 2016. **47**(1): p. 96-103.
24. Lazdam, M., et al., *Prevention of vascular dysfunction after preeclampsia: a potential long-term outcome measure and an emerging goal for treatment.* J Pregnancy, 2012. **2012**: p. 704146.

25. Lazdam, M., et al., *Elevated blood pressure in offspring born premature to hypertensive pregnancy: is endothelial dysfunction the underlying vascular mechanism?* Hypertension, 2010. **56**(1): p. 159-65.
26. Gyselaers, W. and B. Thilaganathan, *Preeclampsia: a gestational cardiorenal syndrome.* J Physiol, 2019. **597**(18): p. 4695-4714.
27. McManus, R.J., et al., *Effect of self-monitoring and medication self-titration on systolic blood pressure in hypertensive patients at high risk of cardiovascular disease: the TASMIN-SR randomized clinical trial.* JAMA, 2014. **312**(8): p. 799-808.
28. McManus, R.J., et al., *Efficacy of self-monitored blood pressure, with or without telemonitoring, for titration of antihypertensive medication (TASMINH4): an unmasked randomised controlled trial.* Lancet, 2018. **391**(10124): p. 949-959.
29. McManus, R.J., et al., *Telemonitoring and self-management in the control of hypertension (TASMINH2): a randomised controlled trial.* Lancet, 2010. **376**(9736): p. 163-72.
30. Topouchian, J., et al., *Clinical accuracy of the Omron M3 Comfort((R)) and the Omron Evolv((R)) for self-blood pressure measurements in pregnancy and pre-eclampsia - validation according to the Universal Standard Protocol.* Vasc Health Risk Manag, 2018. **14**: p. 189-197.
31. Melchiorre, K., et al., *Preeclampsia is associated with persistent postpartum cardiovascular impairment.* Hypertension, 2011. **58**(4): p. 709-15.
32. Gynaecologists, R.C.o.O., *Coronavirus (COVID-19) Infection in Pregnancy*, in *RCOG*. 2020. p. 67.
33. Kitt, J., et al., *New Approaches in Hypertension Management: a Review of Current and Developing Technologies and Their Potential Impact on Hypertension Care.* Curr Hypertens Rep, 2019. **21**(6): p. 44.
34. EXCELLENCE), N.N.I.F.C., *HYPERTENSION IN PREGNANCY: DIAGNOSIS AND MANAGEMENT*, in *NICE.ORG.UK*. 2019.
35. Sheppard, J.P., et al., *Self-monitoring of Blood Pressure in Patients With Hypertension-Related Multimorbidity: Systematic Review and Individual Patient Data Meta-analysis.* Am J Hypertens, 2020. **33**(3): p. 243-251.
36. Boardman, H., et al., *Variations in Cardiovascular Structure, Function, and Geometry in Midlife Associated With a History of Hypertensive Pregnancy.* Hypertension, 2020. **75**(6): p. 1542-1550.
37. Huckstep, O.J., et al., *Physiological Stress Elicits Impaired Left Ventricular Function in Preterm-Born Adults.* J Am Coll Cardiol, 2018. **71**(12): p. 1347-1356.
38. FOUNDATION, B.H. *How healthy is your diet? Questionnaire*. 2012; Available from: www.bhf.org.uk/healthatwork.
39. Ramakrishnan, R., et al., *Accelerometer measured physical activity and the incidence of cardiovascular disease: Evidence from the UK Biobank cohort study.* PLoS Med, 2021. **18**(1): p. e1003487.
40. Haug, E.B., et al., *Life Course Trajectories of Cardiovascular Risk Factors in Women With and Without Hypertensive Disorders in First Pregnancy: The HUNT Study in Norway.* J Am Heart Assoc, 2018. **7**(15): p. e009250.
