# Supplementary material for: Brain Volumes After Hypertensive Pregnancy and Postpartum Blood Pressure Management: A POP-HT Randomized Clinical Trial Imaging Substudy
Source: JAMA Neurol. 2026 Jan 5;83(2):137–44. doi: 10.1001/jamaneurol.2025.5145 (PMC12771390; doi:10.1001/jamaneurol.2025.5145)
Supplement: Supplement 2. — eTable 1. Characteristics of all 220 participants enrolled in POP-HT and participants in the brain MRI sub-study eTable 2. Brain volumes in the intervention and usual care arm eTable 3. Brain volumes of the pre-eclampsia and gestational hypertension groups in the usual care arm eTable 4. Putamen, accumbens, and pallidum volumes of the pre-eclampsia and gestational hypertension groups in the intervention arm [file jamaneurol-e255145-s002.pdf]

Supplemental Online Content

Lapidaire W, Kitt J, Krasner S, et al. Brain volumes after hypertensive pregnancy and postpartum blood pressure management: a POP-HT randomized clinical trial imaging substudy. *JAMA Neurol*. Published online January 5, 2026. doi:10.1001/jamaneurol.2025.5145

eTable 1. Characteristics of all 220 participants enrolled in POP-HT and participants in the brain MRI sub-study
eTable 2. Brain volumes in the intervention and usual care arm
eTable 3. Brain volumes of the pre-eclampsia and gestational hypertension groups in the usual care arm
eTable 4. Putamen, accumbens, and pallidum volumes of the pre-eclampsia and gestational hypertension groups in the intervention arm

This supplemental material has been provided by the authors to give readers additional information about their work.

© 2026 Lapidaire W et al. *JAMA Neurology*.

eTable 1. Characteristics of all 220 participants enrolled in POP-HT and participants in the brain MRI sub-study.

	**Original trial (n=220)**	**Brain MRI group (n=152)**
**Participant characteristics**		
Mean age, y (SD)	33.2 (5.0)	33.5 (5.1)
Mean booking BMI, kg/m² (SD)	28.4 (6.4)	28.8 (6.9)
Mean booking height, cm (SD)	165.2 (7.0)	165.0 (7.1)
Mean systolic blood pressure at first antenatal visit, mmHg (SD)	128.1 (13.5)	127.2 (13.4)
Mean diastolic blood pressure at first antenatal visit, mmHg (SD)	82.2 (10.8)	81.5 (11.1)
Mean duration antenatal antihypertensive treatment, No days (SD)	36 (63)	27 (69)
**Ethnicity, no (%)**		
Black	10 (8)	7 (5)
East Asian	3 (4)	2 (1)
Hispanic	9 (2)	4 (3)
South Asian	15 (8)	10 (7)
White	180 (82)	128 (84)
**Pregnancy characteristics**		
Pre-eclampsiac, No. (%)	133 (60)	96 (63)
Mean gestation at delivery, wks (SD)	38.3 (3.0)	38.1 (3.1)
Previous hypertensive pregnancy, No. (%)	40 (18)	32 (21)
Mean birthweight, kg (SD)	2.9 (0.9)	3.3 (0.6)
Neonatal unit admissions, No. (%)	62 (28)	43 (28)

© 2026 Lapidaire W et al. *JAMA Neurology*.

eTable 2. Brain volumes in the intervention and usual care arm

	Intervention (n=81)	Usual care (n=71)	Adjusted mean difference (95% CI)	P-value	Adjusted p-value
Grey matter volume	567.72 (45.45)	560.78 (42.28)	2.79 (-0.97,6.55)	0.14	0.29
White matter volume	533.94 (46.7)	518.53 (49.19)	**11.50 (2.04,20.96)**	**0.02**	0.17
Cerebrospinal fluid volume	242.83 (33.33)	246.2 (31.59)	-5.59 (-13.10,1.93)	0.14	0.29
Thalamus	15.62 (1.2)	15.37 (1.22)	0.17 (-0.11,0.44)	0.24	0.34
Hippocampus	7.44 (0.77)	7.38 (0.71)	0.01 (-0.19,0.22)	0.90	0.90
Caudate	7.2 (0.75)	7.21 (0.84)	-0.06 (-0.27,0.16)	0.60	0.75
Amygdala	2.28 (0.33)	2.28 (0.35)	-0.01 (-0.11,0.09)	0.84	0.899
Putamen	9.79 (0.86)	9.5 (0.95)	0.24 (-0.01,0.50)	0.06	0.207
Accumbens	0.96 (0.19)	0.91 (0.21)	0.04 (-0.02,0.10)	0.19	0.32
Pallidum	3.49 (0.29)	3.4 (0.31)	0.08 (0.00,0.16)	0.05	0.21

eTable 3. Brain volumes of the pre-eclampsia and gestational hypertension groups in the usual care arm

	Pre-eclampsia (n=45)	Gestational hypertension (n=26)	Adjusted mean difference (95% CI)	P value	Adjusted p value
Grey matter volume, cm^3	564.18 (44.94)	554.89 (37.33)	-2.40 (-7.76,2.97)	0.38	0.54
White matter volume, cm^3	517.59 (50.72)	520.16 (47.35)	-14.02 (-28.52,0.48)	0.06	0.15
Cerebrospinal fluid volume, cm^3	250.34 (33.86)	239.04 (26.3)	4.79 (-5.95,15.53)	0.38	0.54
Thalamus, cm^3	15.42 (1.22)	15.28 (1.24)	-0.10 (-0.53,0.34)	0.65	0.73
Hippocampus, cm^3	7.45 (0.67)	7.27 (0.76)	0.09 (-0.22,0.41)	0.56	0.69
Caudate, cm^3	7.18 (0.91)	7.26 (0.72)	-0.20 (-0.56,0.16)	0.28	0.54
Amygdala, cm^3	2.29 (0.35)	2.25 (0.34)	0.00 (-0.16,0.17)	0.96	0.96
Putamen, cm^3	**9.25 (0.98)**	**9.93 (0.75)**	**-0.83 (-1.20,-0.46)**	**<0.001**	**<0.001**
Accumbens, cm^3	**0.86 (0.22)**	**0.99 (0.16)**	**-0.15 (-0.24,-0.05)**	**0.003**	**0.02**
Pallidum, cm^3	**3.37 (0.32)**	**3.45 (0.29)**	**-0.13 (-0.26,-0.01)**	**0.04**	0.13

eTable 4. Putamen, accumbens, and pallidum volumes of the pre-eclampsia and gestational hypertension groups in the intervention arm

	Pre-eclampsia (n=51)	Gestational hypertension (n=30)	Adjusted mean difference (95% CI)	P value	Adjusted p value
Putamen, cm^3	9.81 (0.82)	9.74 (0.93)	0.13 (-0.21,0.48)	0.439	0.439
Accumbens, cm^3	0.99 (0.19)	0.90 (0.19)	**0.09 (0.01,0.18)**	**0.025**	0.075
Pallidum, cm^3	3.5 (0.29)	3.48 (0.3)	0.05 (-0.07,0.16)	0.427	0.439

© 2026 Lapidaire W et al. *JAMA Neurology*.
