# Supplementary material for: Brain Volumes After Hypertensive Pregnancy and Postpartum Blood Pressure Management: A POP-HT Randomized Clinical Trial Imaging Substudy
Source: JAMA Neurol. 2026 Jan 5;83(2):137–44. doi: 10.1001/jamaneurol.2025.5145 (PMC12771390; doi:10.1001/jamaneurol.2025.5145)
Supplement: Supplement 3. — Data sharing statement [file jamaneurol-e255145-s003.pdf]

Data Sharing Statement

Lapidaire. Brain Volumes After Hypertensive Pregnancy and Postpartum Blood Pressure Management. *JAMA Neurol*. Published January 05, 2026. doi:10.1001/jamaneurol.2025.5145

Data
Additional Information: https://clinicaltrials.gov/study/NCT04273854
Data available: Yes
Data types: Deidentified participant data
How to access data: The data that support the findings of this study are available from the chief investigator [PL] upon reasonable request subject to the approval of the Sponsor [University of Oxford] and the Trial Steering Committee.
When available: With publication

Supporting Documents
Document types: Statistical/analytic code
How to access documents: The trial statistical analysis plan will be published with the article
When available: With publication

Additional Information
Who can access the data: The data that support the findings of this study are available from the chief investigator [PL] upon reasonable request subject to the approval of the Sponsor [University of Oxford] and the Trial Steering Committee.
Types of analyses: Specified purpose
Mechanisms of data availability: The data that support the findings of this study are available from the chief investigator [PL] upon reasonable request subject to the approval of the Sponsor [University of Oxford] and the Trial Steering Committee.
